# Immunoglobulin replacement therapy in primary immunodeficiency disorders: Pragmatic review and evidence mapping

**DOI:** 10.1016/j.jacig.2026.100685

**Published:** 2026-03-19

**Authors:** Emma Carr, Rachael McCool, Lavinia Ferrante di Ruffano, Mick Arber, Katie Reddish, James Burns, Riahn Holcomb-Selbert, Manar Abdalgani, Jordan S. Orange

**Affiliations:** a York Health Economics Consortium, Enterprise House, Innovation Way, University of York, York, United Kingdom; b Quantics Biostatistics, Edinburgh, United Kingdom; c Department of Pediatrics, Columbia University Vagelos College of Physicians and Surgeons, Columbia University Irving Medical Center, New York, NY; d Children's Hospital of Philadelphia, Philadelphia, Pa

**Keywords:** Primary immunodeficiency disorder, scoping review, feasibility assessment, immunoglobulin replacement therapy

## Abstract

**Background:**

Immunoglobulin replacement therapy (IgRT) is used in the treatment of patients with primary immunodeficiency disorders (PIDDs). There are some references to differences between preparations in terms of characteristics and efficacy, though comparative evidence is limited.

**Objectives:**

We sought to identify and map the evidence-base of clinical outcomes and adverse events of IgRT studies used for the treatment of patients with PIDDs and determine any signals of difference in treatment effect between different immunoglobulin brands.

**Methods:**

A pragmatic literature search was conducted in February 2023 to identify studies assessing the efficacy of IgRT in PIDDs, and an assessment of the feasibility of indirect treatment comparisons (ITC) was carried out. Annualized outcome data were presented in visualization plots and possible outlier results identified through naive (unanchored, unadjusted) comparisons; results were considered outliers when no overlap in confidence interval was identified.

**Results:**

After single-reviewer screening, 103 studies were included; 70 prospective studies were prioritized for extraction. A feasibility assessment found that ITC was not possible. Few outlier results for any particular commercial IgRT brand were identified across the outcomes considered, and those that were identified may have been due to differences in study methods or intervention characteristics rather than differing efficacy.

**Conclusions:**

Limited evidence on the comparative efficacy of different IgRT brands was identified. Reporting in studies of IgRT for PIDDs was found to vary widely, such that ITC was not possible. We recommend improving reporting to enable such comparisons in the future, including suggestions on improving or standardizing reporting of patient and study characteristics, outcome definitions, and follow-up duration and measures of variance.

Primary immunodeficiency disorders (PIDDs), also referred to as inborn errors of immunity, are a group of over 500 disorders largely caused by genetic defects that impair immunity.^1^ The signs and symptoms of PIDDs depend on which parts of the immune system are affected, but they often include frequent, severe, and atypical infections.^2^ Extensive noninfectious complications including autoimmunity, autoinflammation, allergy, and malignancy are also well characterized.^2^^,^^3^ Importantly, repeated infection in PIDD patients can result in organ system complications such as chronic lung disease as well as reduced quality of life.^2^^,^^4^, ^5^, ^6^

Predominantly antibody deficiencies are a major subset of PIDD (including over 50 disorders in the latest International Union of Immunological Societies classification document^7^) in which the major clinically relevant immunodeficiency affects humoral immune function. Many patients with predominantly antibody deficiencies have a clinically relevant inability to effectively produce immunoglobulin^1^ and require regular infusions with immunoglobulin to replenish their low, absent, or ineffective IgG, referred to as immunoglobulin replacement therapy (IgRT).^8^ IgRT is administered intravenously (IVIG) or subcutaneously (SCIG). Both routes are highly effective at preventing serious bacterial infection (SBI) while also providing other beneficial outcomes.^9^

Immunoglobulin preparations for use as IgRT are produced by numerous manufacturers and all within detailed specific regulatory guidelines.^10^ All preparations are derived from pooled human plasma to allow for broad immunity, and all must achieve “noninferiority” to obtain licensing approval. Approved immunoglobulin preparations must therefore have achieved standards of both efficacy and constitution.^11^ That said, there have been occasional references to differences between preparations in terms of process and characteristics, as well as some even related to efficacy, tolerability, and outcome. Following this logic and expert consensus, the American Academy of Allergy, Asthma & Immunology’s guiding principles for IgRT consider immunoglobulin products to not be interchangeable.^9^ Existing meta-analyses have investigated whether IVIG or SCIG preparation types are more effective, and have shown mixed results.^12^^,^^13^ To date, there have been only a few attempts to directly compare individual immunoglobulin preparations against each other.^14^, ^15^, ^16^ Although the standards indicate that products are not interchangeable, there is scant evidence substantiating any differentiation between preparations and attributes of the preparations with regard to efficacy and outcome.

In an effort to determine if there might be any comparative signals in the existing studies of IgRT, a pragmatic review was conducted to identify and interrogate published evidence for commercially available brands of IVIG and SCIG preparations. Specific attention was given to their reported outcomes in the interests of comparing the relative efficacy and tolerability between them if possible.

The objectives of this project were: (1) to identify and map the evidence base of clinical outcomes and adverse events (AEs) of IgRT studies used for the treatment of patients with PIDDs; (2) to review and report in aggregate on the reported clinical outcomes and AEs of different commercially produced brands of IgRT and explore possibilities for indirect treatment comparisons (ITCs); and (3) to determine if there were any signals of difference between clinical studies of immunoglobulin and potential trends.

## Methods

### Identifying studies

Searches of Medline (OvidSP), Cochrane Library, and the HTA (Health Technology Assessment) Database were conducted in February 2023 to identify eligible studies. Search strategy design and search resource selection reflected the pragmatic review context and did not include gray literature (see Table E1 and Fig E1 in the Online Repository available at www.jaci-global.org). Systematic reviews (published 2019 to 2023) were also checked for any eligible studies that may have been missed by the database searches.

Eligible studies (criteria in Table I) evaluated patients of any age with a diagnosis of PIDD (not further specified), predominantly antibody deficiency (not further specified), or any of 4 common specific PIDD types, who received IgRT using any brand-identifiable IVIG or SCIG preparation administered at a mean dose of ≥400 mg/kg per month. Studies published before 2000 were excluded to ensure that included studies assessed efficacy using the US Food and Drug Administration (FDA) Blood Products Advisory Committee requirement to demonstrate noninferior efficacy, using an annualized SBI rate of ≤1 (at the .01 level of significance).^17^

**Table I:** Eligibility criteria

Characteristic	Inclusion criteria	Exclusion criteria
Population	Studies of patients of any age with a diagnosis of PIDD (not further specified), primary antibody deficiency (not further specified), hypogammaglobulinemia associated with a primary cause, or one of the following specified PIDDs: • CVID. • Agammaglobulinemia. • Hypogammaglobulinemia. • SAD. Studies of patients with other specified PIDDs (not listed above) were eligible if ≥80% of study population fell within the 4 categories listed above.	Studies of patients with specified PIDDs not listed in inclusion criteria, unless at least 80% of study patients have a diagnosis of 1 of 4 specified PIDDs. Studies with mixed PIDD and non-PIDD populations were not eligible unless eligible PIDD patient population constituted at least 80% of total number of patients.
Interventions	Any named IVIG or SCIG preparation administered at a dose of ≥400 mg/kg per month. The brand name of the preparation must be identifiable.	Intramuscular immunoglobulin preparations. Studies of mean doses <400 mg/kg per month. Studies in which brand name is not identifiable or cannot be inferred (eg, by reporting a corporate sponsor).
Comparators	Any named IVIG or SCIG preparation. Placebo. No comparator (in the case of single-arm study designs).	None.
Outcomes	Studies reporting any clinical effect or safety outcome, including: • IgG levels. • Quality-of-life measurements (eg, SF-12, SF-36). • Acute SBI rate (including pneumonia). • Overall clinical infection rate. • Infection rate of any kind. • Hospital admissions/length of hospitalization. • Physician visits due to illness or infection. • Days on antibiotics (prophylactic or therapeutic). • Days missed from school/work. • Overall AEs. • Serious AEs. • AEs of any kind. • Withdrawals due to AEs. • Transition to alternative product due to intolerance or preference.	None.
Study design	Studies analyzing ≥10 eligible patients using one of the following study designs: • RCTs. • Comparative observational studies (prospective or retrospective). • Single-arm studies (observational or interventional).	Studies with <10 participants. Case reports. Reviews of primary evidence (relevant systematic reviews published since 2018 were checked for eligible primary studies).
	Studies published from 2000 onward. English-language studies only.	Study published before 2000. Study published as preprint only. Conference abstracts. Editorials or news items. Non-English language.

*CVID,* Common variable immunodeficiency; *SAD,* specific antibody deficiency; *SF-12,* 12-Item Short Form Health Survey; *SF-36,* 36-Item Short Form Health Survey.

One experienced reviewer (L.F.R., E.C.) screened search results at title and abstract then full-text review, with verification of decisions for 10% of screened records by a second reviewer (R.M., L.F.R.). Any disagreements were resolved through discussion.

Data extraction was carried out in two stages. All identified studies were mapped to better understand the available study designs and outcomes. Prospective studies were then prioritized for full extraction and synthesis. One reviewer extracted data for each of the eligible studies, with a second reviewer checking all data points. Discrepancies were resolved by a third reviewer. Risk of bias assessment was conducted by an adapted version of a previously reported quality assurance tool.^18^ A data extraction sheet drafted in Excel was piloted on 5 studies before full data extraction.

The following elements were extracted from all eligible studies: bibliographic details, study characteristics, patient baseline characteristics, intervention details, details of statistical analyses, and eligible outcomes. The protocol was registered in the Open Science Framework as A7h4d. A complete list of data extraction elements is provided in Appendix 1 in the Online Repository available at www.jaci-global.org.

### Synthesis

A feasibility assessment was conducted to determine the appropriateness of performing ITCs of different IgRT brands by means of network meta-analysis. Statistical pooling was not considered to be appropriate because of the considerable between-study heterogeneity in patient groups and study design, and the scarcity of comparable outcome data for similar interventions. Key outcome data are therefore summarized in tables and visualized in plots.

Studies were considered together whether they involved fixed-length or variable-length follow-up across subjects. For studies where the length of follow-up varied across subjects, the outcome was standardized to the annual rate by a Poisson model. The 95% confidence intervals (CIs) were calculated by the ‘exact’ method.

For the single continuous outcome (immunoglobulin trough level), calculated CIs for the mean value were normal based, generated assuming normality of the mean’s sampling distribution. CIs are provided for illustrative purposes only.

For the single binary outcomes with equal follow-up across subjects (AE occurrence), we calculated 95% CIs for the overall proportion by the Clopper-Pearson method.

To be included in the visualization plots, studies had to report either (1) the mean rate with a 2-sided 95% CI (or a 1-sided [upper] 99% CI for SBI); or (2) both the total number of events and total patient-years of follow-up (or mean and standard deviation in the case of immunoglobulin trough level) to allow for these quantities to be calculated. For the SBI plot, the 1-sided intervals were plotted as 2-sided intervals with the lower limit at the minimum possible value, 0. In cases where there was a reported CI and a calculated interval was possible for a study outcome, the least precise (ie, widest) CI was used.

Possible outlier results were identified through naive (unanchored, unadjusted) comparisons. CIs for each result (as reported or calculated where sufficient data were reported) were checked for overlap between studies. Results were considered outliers when no overlap in CI was identified, and the point estimate was at an extreme of the range of estimates observed across all included studies.

It is important to highlight that these naive comparisons are based on summary-level data where treatments cannot be connected by a common comparator, and thus we provide them with the caveat that they do not account for heterogenicity across studies (which is likely to be considerable as a result of varied reporting of population characteristics and the fact that most evidence consists of nonrandomized single-arm studies) and therefore should be interpreted with extreme caution.

For binary outcomes and rates measured over specific lengths of time, studies were grouped according to length of follow-up, and only studies with similar lengths of follow-up were selected for presentation together.

## Results

### Identification of studies

Database searches identified 1,767 records. A further 5 records were identified from other sources such as systematic review reference and errata checking. After deduplication, 1,692 records were screened by title and abstract; then 363 records underwent full-text review, after which 103 studies (reported in 138 records) were included. Of these, 70 prospective studies (reported in 101 records) underwent full data extraction and were included in the feasibility assessment.

**Fig 1:**
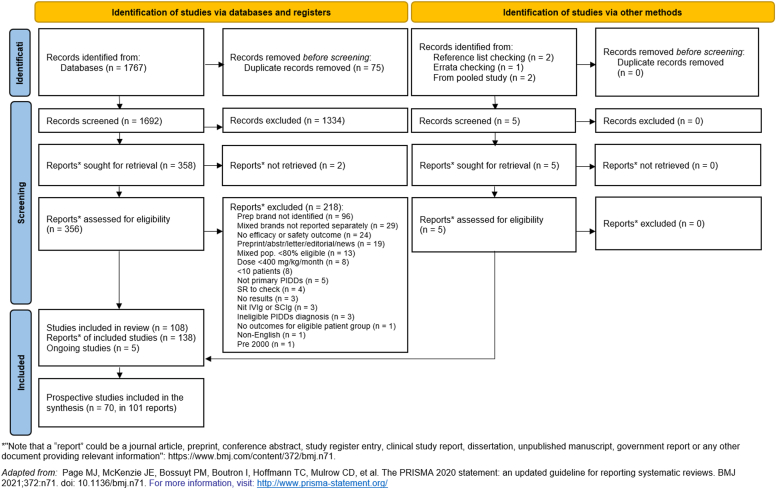
PRISMA (Preferred Reporting Items for Systematic Reviews and Meta-analysis) study selection diagram.

The full record selection process is presented in (Fig 1). The included studies are presented in Table E2 in the Online Repository available at www.jaci-global.org; the list of excluded studies is available on request.

### Overview of included studies

Of the 70 included prospective studies, 21 provided comparative data for ≥2 different interventions, formulations, or administration routes. This included 9 between-person comparisons (different populations in 2 treatment arms) including 3 randomized controlled trials (RCTs),^14^^,^^19^^,^^20^ 5 crossover RCTs,^21^, ^22^, ^23^, ^24^, ^25^ 1 prospective cohort study,^26^ and 12 within-person comparisons (before-and-after studies in which the same population received different interventions/formulations);^15^^,^^16^^,^^27^, ^28^, ^29^, ^30^, ^31^, ^32^, ^33^, ^34^, ^35^, ^36^ 49 studies provided single-arm data. Detailed study characteristics are presented in Table E2.

Some patient characteristics varied between studies while others were inconsistently reported. Areas of heterogeneity were noted in relation to most characteristics including age, PIDD diagnosis, previous immunoglobulin treatment, and preexisting illness. Detailed patient characteristics for each study are presented in Table E3 in the Online Repository available at www.jaci-global.org.

Intervention details, including dosage, were in many places incompletely reported or not included at all. The most notable areas of heterogeneity within each brand of immunoglobulin formulation included variations in dose according to individual subject and exact number of infusion sites per subject. Further, the concomitant use of prophylactic antibiotics was not as consistently reported in older studies. There was also variability in reporting across studies, which could potentially affect the rates of infection in any given study.

Options were explored for network meta-analysis to derive comparative estimates across the products identified. Among the 9 between-person comparative studies, 3 compared different immunoglobulin products, including one RCT comparing Gamunex 10% and Gamimune N 10%, one RCT comparing Flebogamma 5% and Flebogamma 10%, one crossover RCT comparing Endoglobulin 5% and Gammabulin 16%, and one crossover RCT comparing Gamunex 5% to Gamunex 10%. All remaining studies compared the same immunoglobulin brand applied with different administration routes or regimens. No between-person studies shared a common treatment arm to allow for indirect comparisons.

Among the 12 within-person studies, there was some overlap in the treatments assessed in 4 trials, but the resulting network only facilitated comparisons between different concentrations of Gammagard with Subcuvia and Cuvitru (already assessed in a comparative study^28^), and not all studies reported data for each outcome.

Because of the heterogeneity across studies, the fact that so few studies evaluated common comparators, and the small number of studies reporting similar preparations, statistical pooling was not considered useful. In other words, while some outcome measures were the same (ie, SBI incidence), the individual variables of a study allowing for ITC were different enough that ITC or other pooled analyses were determined not to be appropriate.

### Results of included studies

Despite the difficulties we had comparing treatments across IgRT studies, there were key data outcomes identified across the included studies, which we summarize in Table E4 in the Online Repository available at www.jaci-global.org.

**Fig 2:**
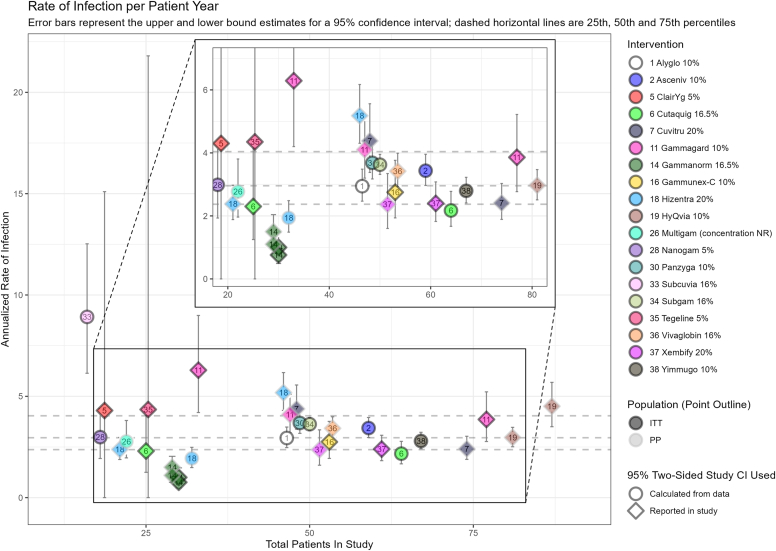
Annualized rate of infection.

**Fig 3:**
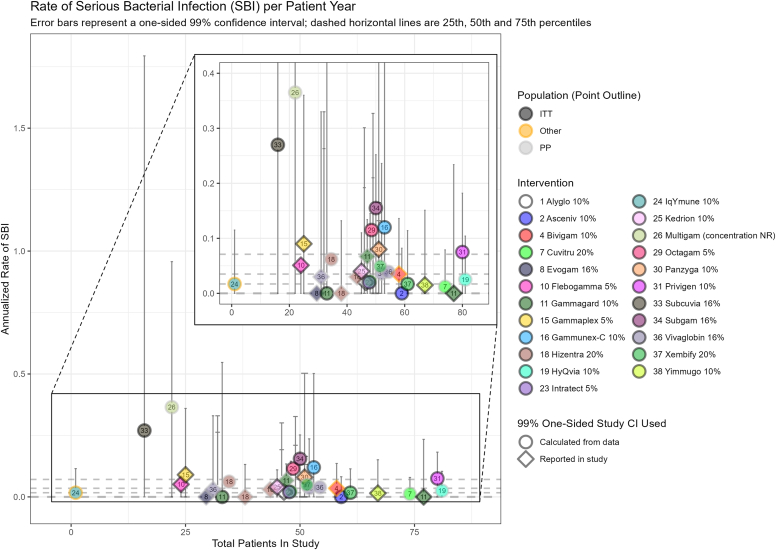
Annualized rate of SBIs.

**Fig 4:**
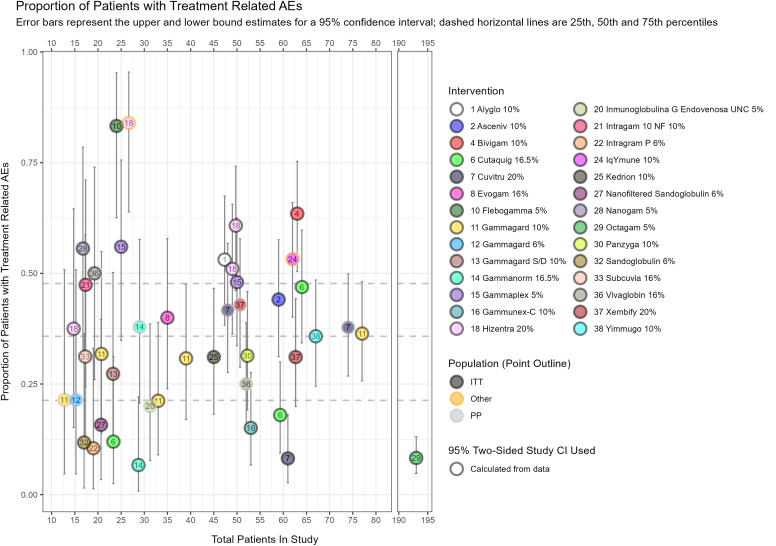
TRAEs.

Studies that reported 95% CIs (or 99% upper CI for SBI), or sufficient data for these to be calculated, were included in plots. The most data were available for annualized rates of infection (Fig 2), SBI (Fig 3), and treatment-related AEs (Fig 4), with fewer data present for hospitalizations (see Fig E2 in the Online Repository available at www.jaci-global.org), mean trough levels (see Fig E3 in the Online Repository), antibiotic receipt (see Fig E4 in the Online Repository), and school/work days missed (see Fig E5 in the Online Repository). Data were also plotted for annualized infection rate and treatment-related AE rate plotted by mean immunoglobulin trough level (see Fig E6 and Fig E7, respectively, in the Online Repository).

### Annualized rate of infection

A total of 43 studies reported an annualized rate of infection, of which only 22 reported sufficient data to allow for interpretation and inclusion in a plot of the data. The other 21 studies reported the annualized infection rate alongside range, interquartile range, another level of CI, or no measure of variance, and did not report sufficient information to allow for calculation of the 95% CI. Nineteen different immunoglobulin preparations were assessed across the 22 plotted studies. Five preparations were assessed by more than one study (see Table E5 in the Online Repository available at www.jaci-global.org). The annualized infection rates ranged from 0.76 (95% CI, 0.49 to 1.20) (Gammanorm 16.5% rapid pump^22^) to 8.92 (95% CI, 6.36 to 12.09) (Subcuvia 16%^28^), and considerable variation was also noted in the annualized rates reported within individual immunoglobulin preparations (see Table E5 in the Online Repository).

### Annualized rate of SBI

Thirty-nine studies reported an annualized SBI rate, of which 27 reported sufficient data to allow for interpretation and inclusion in a visualization plot.

The 27 studies included in the plot assessed 23 immunoglobulin preparations, with 5 assessed by more than one study (Table E5). The annualized SBI rates ranged from 0 to 0.365 (99% upper CI, 0.957) (Multigam^37^) in the studies that were plotted (Fig 3).

### Treatment-related AEs

Treatment-related AE (TRAE) data were reported over a range of follow-up periods and using a variety of different approaches. Because of the variation in reporting across the studies, a number of assumptions were made. Studies reporting TRAEs described as “definitely,” “possibly,” or “plausibly” related AEs are included. In some cases, it is stated that infections are excluded from reported AEs, but in other cases, this is not stated. With the assumption that infections would not be reported as an AE unless stated, studies with both types of reporting are included. Both types of studies were included in generating aggregate data for visualization plotting (Fig 4). The term *treatment-emergent AE* was assumed to mean any AE occurring within the treatment period and does not indicate that the AEs are treatment related unless specifically stated, and thus these were not included in the visualization plot. We also note that intervention characteristics that could affect TRAEs such as infusion premedication and infusion rate were not well reported across studies.

A total of 28 immunoglobulin preparations were assessed in 35 studies, with 8 assessed by more than one trial (Table E6). TRAEs were experienced by 6.7% (Gammanorm 16.5%^22^) to 84% (Hizentra 20%^38^) of patients across all but 2 treatment arms.

## Discussion

This pragmatic review aimed to identify and map the evidence of the efficacy of different immunoglobulin preparations used as IgRT in PIDDs. While there is perception of differing efficacy and tolerability across different immunoglobulin preparations, there is limited comparative evidence to determine whether this is the case. Previous reviews have evaluated the relative efficacy of SCIG and IVIG administration routes^13^ and the relationship between immunoglobulin dose and trough levels with clinical outcomes^12^^,^^39^^,^^40^ rather than the relative efficacy of different products. There was a paucity of head-to-head comparative evidence between immunoglobulin products^40^ (or between particular concentration strengths^12^) and a small number of studies investigating the same product for pooling.^39^ Previous review authors have also noted difficulty comparing across studies as a result of heterogeneity in patient diagnoses,^39^ dosing, and outcome definition (with specific recommendation for more uniform definitions for infection and AEs).^40^ Other reviews have found limited reporting of intervention characteristics such as dosing and number of infusion sites, and have also found that limited methods reporting impedes thorough quality assessment.^41^

### Summary of findings

We identified 70 prospective studies in this review that evaluated 34 different immunoglobulin preparations used for IgRT in PIDDs. Twenty-one studies provided comparative data for ≥2 different interventions, formulations, or administration routes; these comprised 9 between-person comparisons and 12 within-person comparisons. A total of 49 studies provided single-arm data only.

Of the 43 studies reporting annualized rate of infection, only 22 studies reported 95% CI or sufficient data to calculate it; 19 different immunoglobulin preparations were assessed. There was considerable variation both across and within immunoglobulin preparations, with rates ranging from 0.76 (95% CI, 0.49 to 1.20) to 8.92 (95% CI, 6.36 to 12.09). For SBI, of the 39 studies reporting an annualized SBI rate, 27 reported 99% CIs or sufficient data to calculate them, and rates ranged from 0 to 0.365 (99% upper CI, 0.957). A total of 35 studies reported the proportion of patients experiencing TRAE, which varied considerably, from 6.7% to 84%. We note that intervention characteristics that could affect TRAEs such as infusion premedication and infusion rate were not well reported across studies.

### Limitations of evidence

Where reported, the date of patient recruitment ranged from 1994 to 2020, during which time the practice of immunoglobulin therapy may have changed. SCIG was approved by the FDA in 2006, and since then, there has been ongoing refinement in its dosing, administration, and application. Included studies were conducted in a range of countries, which may also affect comparability. There are differing requirements of some of the licensing agencies such as the FDA and the European Medicines Agency (notably dose conversion from intravenous to subcutaneous routes of administration).^42^ It is also likely that outcomes such as missed work/school and quality of life might have different characteristics across nations owing to different social norms and should be considered within that context.

All studies were assessed for risk of bias using a widely applied tool^18^ and were considered to have a moderate to high risk of bias. Common issues identified were in relation to limited reporting of methods and differences in baseline populations or insufficient information to make a judgment. The full risk of bias assessment is presented in Table E7 in the Online Repository available at www.jaci-global.org. Risks of bias are in part reflective of the challenges that are encountered when designing studies in the arena of rare diseases, but they also highlight the need for consistent reporting guidance. Indeed, it underscores a challenge with the noninferiority SBI criteria and a lack of requirement for consistency in reporting/definitions in IgRT pivotal trials. A formal assessment of the certainty of evidence (eg, GRADE [Grades of Recommendation Assessment, Development, and Evaluation]) was not performed because this was a pragmatic scoping review involving heterogeneous data without meta-analysis.

We explored options for ITC, but the small number of comparative studies with common treatment arms meant that the available network would have only facilitated comparisons between different concentrations of Gammagard with Subcuvia and Cuvitru (which were already assessed directly in a comparative study^28^). Furthermore, the differences in reporting, definitions, and variability in application prevented IgRT from being considered the common arm. More specifically, naive pooling was not considered to be appropriate because of the observed heterogeneity across patient populations, outcomes, and study designs identified in this review.

Visualization plots of key outcome data were produced on the basis of common measures that were similarly reported in the included studies. For each key annualized outcome, only studies that reported (or permitted the calculation of) the required CIs were included, although these need to be interpreted with caution.

An additional 14 studies, of 7 different products, have been published since our searches were conducted. None of the identified studies was comparative in nature, so our conclusions around the availability of data to draw comparative conclusions remain applicable. However, the identified studies may provide additional data points on many of the visualizations we present here. We also note that 6 of the 14 identified studies were of a single preparation (HyQvia), so any consideration of this particular immunoglobulin should not be based exclusively on the work here, which only included a single study of this preparation.

To facilitate future cross-study comparisons of IgRT trials in PIDD populations, more consistent and comprehensive reporting of patient characteristics, outcome definitions, and outcome data is required. Published studies would benefit from including complete and consistent data not only on treatment effects but also on measures of variance, such as standard deviations or CIs. While this additional information is likely generated during study execution, it is often not included in submitted manuscripts or required by journals. Providing patient-level data as supplementary material or appendixes would provide additional transparency and opportunity to improve availability of data for evidence synthesis and comparisons in this rare disease population. The scarcity of patients living with PIDDs and their willingness to participate in IgRT studies begs enhancement of data reporting.

Despite this being a crucial evidence base for lifesaving PIDD therapies, reporting consistency and detail have not improved over time—something potentially influenced by the ongoing requirement for noninferiority study design as a prerequisite for regulatory approval. Standardization initiatives promoting uniform outcome measure reporting across studies should be considered to strengthen the quality of the IgRT evidence base. More granular reporting of outcome definitions and patient-level data would improve options for evidence synthesis to advance clinical understanding and therapeutic strategies for PIDD patients.

### Limitations of this review

A number of limitations owing to the pragmatic nature of this review must be considered when interpreting its findings.

The search methods incorporated pragmatic decisions to reduce the number of records retrieved for screening. These included population concepts designed only to retrieve records referring to nonspecific PIDDs or a limited number of specific PIDD conditions and intervention concepts designed only to retrieve records referring explicitly to immunoglobulins (not including *antibody* as a variant term) with subcutaneous or intravenous administration. Further details of the search methods and pragmatic decisions are provided in Appendix 1 in the Online Repository available at www.jaci-global.org.

Study selection was carried out by a single reviewer with a 10% check by a second reviewer. We note that this approach does not conform to systematic review guidelines but was considered to be appropriate in the context of a pragmatic review, which is what we have done here, as labeled in our study’s title as well as throughout the report. After single-reviewer study selection, a greater-than-anticipated amount of literature was included, and a protocol amendment was agreed to only extract and synthesize prospective evidence.

Outcome data were synthesized when reported at similar time points with sufficient statistical information for inclusion in the data plots, though outcome definitions and measures were not always clearly reported. Naive comparisons identified potential outlier results but do not account for heterogeneity. Visual inspection of overlapping CIs does not constitute formal statistical testing, and we note that the small sample sizes in the identified studies (and resulting wide CIs) could be masking true variation. It is not possible to offer a statistical test to evaluate a null hypothesis, so conclusions should not be drawn here that are based on a visual inspection of what we present. Exploratory plotting of mean immunoglobulin levels by outcome data was conducted to visualize possible relationships; however, the data allowed the plotting of just 2 outcomes (infection rate and proportion of patients experiencing AEs), with 5 to 6 available studies.

### Conclusion and recommendations

This pragmatic review aimed to identify and map the evidence of the efficacy of different immunoglobulin preparations for PIDDs. We found that reporting in studies of IgRT for PIDDs varies widely, with patient and study characteristics often provided in different ways, outcomes defined in different or unclear ways, and outcome data reported without measures of variance. Robust comparisons across IgRT brands could not be made.

### Recommendations for future research

One of the key challenges to assessing the comparative efficacy and safety of the studies is the reporting of key information in relation to patient characteristics, definitions, and interventions to establish whether studies are similar enough to draw meaningful comparisons. The current variability in reporting makes it difficult to draw comparisons across studies and available preparations, or to identify where a specific preparation may address a particular patient need. Recommendations for improving reporting are summarized in Table II. This is especially important given the challenges in designing trials in this area due to the limited numbers of PIDD patients available for studies of IgRT and would provide the opportunity to improve approaches and therapies by enabling these comparisons. While we acknowledge the essential and lifesaving nature of IgRT for PIDD patients (and acknowledge the importance of noninferiority end points), we believe it would be in the community’s best interest to utilize more standardized definitions and make more of the patient-level data available to allow for continued innovation in the field and on behalf of PIDD patients.

**Key messages**

• Reporting of patient and study characteristics, outcome definitions, and outcome data in studies of IgRT for PIDDs varies widely, preventing robust comparisons across IgRT brands.
• We propose recommendations for improving reporting.

**Table II:** Recommendations for reporting

Domain	Issues observed in this review	Recommendation
Study designs	• Because of the limited number of comparative studies, traditional meta-analysis and network meta-analysis were not feasible. • Naive comparisons were not considered appropriate because of the limited reporting of patient characteristics and the observed heterogeneity where they were reported.	• When considering options for statistical pooling ITCs, the most robust evidence to consider is RCT evidence. To create a network, studies must have at least 2 treatment arms and a common comparator with another study to facilitate network links. • Alternatively, more robust, population-adjusted analyses could be conducted if individual patient data were available from single-arm or comparative studies. • We recommend making individual patient data available in supplementary material or appendixes in published articles.
Patient characteristics	• In this review, where reported, variation in patient characteristics identified was generally considered to reflect PIDDs in a real-world clinical setting. However, in many studies, it was difficult to assess which patients were included in the study and how they might differ from others, and an assessment of heterogeneity could not be made.	• Key patient information (age, PIDD type, details of preexisting illness) should be reported to allow assessment of heterogeneity across study populations and individual studies.
Interventions characteristics	• Intervention characteristics, particularly dosage, varied between studies; others were not often available. Details of infusion rate and criteria for premedicalization were generally not reported.	• Clear reporting of intervention characteristics is important to allow assessment of the suitability of comparison across studies (ie, whether studies of the same intervention are actually assessing the same thing). • At minimum, immunoglobulin product, dose, dosing schedules, and administration routes should be reported, along with full details of any permitted concomitant therapies such as antibiotics. • Reports should consider standardized premedication regimens or adhering to FDA recommendations for discouraging premedication, especially for a product that a patient has never before received.
Outcomes	• Several outcomes were assessed across the identified studies, but the most common were annualized rates for SBI, overall infection, hospitalizations, school/work days missed, and antibiotic therapy. However, there were noted variabilities. • *Variabilities in definition:* The way that these outcomes were defined and reported varied across studies, which meant it was not always possible to determine whether the studies were assessing comparable things. • *Variabilities in reporting of data:* Most reported annualized event rate, but some reported average value only (with no measure of variance); some reported different measures of variance that cannot be compared (eg, 90% CI and 99% CI). We also identified variability across several studies in the reported mean and CI (we calculated mean and CI where data permitted this). The reports described no standardized adjustments to account for these differences and thus allow for their comparability. • *Variabilities in time point of assessment/follow-up:* It was not always clear what time frame data were reported. Depending on the outcome, extrapolating from 3 months to an annual rate could over- or underestimate the true rate. Time of year may also influence outcomes like infection, and we could not always discern these variables.	• Outcomes should be clearly defined. • Data should report number of episodes or patients with episode. • While difficult, efforts should be made to use standardized definitions of infection in PIDD patients. • For outcomes like requirement for antibiotics, studies should be clear about whether this includes prophylactic and/or therapeutic use; these should be provided and considered separately. • Details regarding prescribed regimens should be provided (ie, prophylaxis provided during entire study period or just part, and for what). • When reporting overall rates of something that overlaps with another outcome, it should be made clear whether this includes or excludes the overlapping outcome (eg, overall infection rate; does this include SBI?). • Outcome data should be reported alongside a measure of variance that illustrates the uncertainty around the estimate. • For annualized outcome data, means and 95% CIs should be reported, alongside the number of events and patient-years to allow calculation if required. • Standard CI reporting should be adopted for all IgRT studies in PIDDs. • For time point of assessment/follow-up, clear reporting of study dates and duration of assessment should be provided to allow for any seasonality or specific overarching events (ie, COVID-19).
Data availability	• Available data were only those reported along with the publication of studies, although others are available in prescriber information but are difficult to cross-reference.	• Individual patient data should be made available as supplementary materials or appendixes to publications of IgRT studies in PIDD populations.

## Disclosure statement

This report was commissioned, paid for, and conducted in association with Columbia University and was supported by the 10.13039/100002350St Giles Foundation and an investigator-initiated research grant from ADMA Biologics to Columbia University Vagelos College of Physicians and Surgeons (to J.S.O.).

Disclosure of potential conflict of interest: E. Carr, R. McCool, L. Ferrante di Ruffano, M. Arber, and K. Reddish conducted this review as part of their employment at YHEC and declare no conflicts of interest. J. Burns and R. Holcomb-Selbert conducted statistical analysis as commissioned by YHEC and declare no conflicts of interest. J. S. Orange declares ongoing consultancy for ADMA Biologics, CSL Behring, Grifols, Takeda and Teva, and has previously been in receipt of a research grant from ADMA Biologics. M. Abdalgani declares no relevant conflicts of interest.
