# Supplementary material for: Immunoglobulin replacement therapy in primary immunodeficiency disorders: Pragmatic review and evidence mapping
Source: J Allergy Clin Immunol Glob. 2026 Mar 19;5(4):100685. doi: 10.1016/j.jacig.2026.100685 (PMC13098416; doi:10.1016/j.jacig.2026.100685)
Supplement: Supplementary Figs and Tables [file mmc1.docx]

# Appendices

## Appendices

## Search Methods

### Literature Search Methods

### Search Strategy

A MEDLINE (OvidSP) search strategy was designed to identify studies reporting on the use of IVIG or SCIG in populations with a diagnosis of non-specific PIDD or one of the eligible specific PIDDs (CVID, agammaglobulinemia, hypogammaglobulinemia, SAD). The final MEDLINE strategy is presented in Figure E1.

The main structure of the strategy comprised seven concepts:

- Non-specific PIDD (search lines 1 to 17).
- CVID (search lines 18 to 21).
- Agammaglobulinemia / hypogammaglobulinemia (search lines 22 to 33).
- SAD (search lines 34 to 43).
- Ig (search lines 45 to 56).
- Subcutaneous administration (search lines 57 to 64).
- Intravenous administration (search lines 65 to 70).

The concepts were combined as follows:

(non-specific PIDD OR CVID OR agammaglobulinemia / hypogammaglobulinemia OR SAD) AND Ig AND (subcutaneous administration OR intravenous administration).

The strategy also included a separate search strand that combined population terms with pre-combined terms for IVIG / SCIG (search lines 72 to 90).

The strategy was devised using a combination of subject indexing terms and free text search terms in the Title, Abstract, Keyword Heading Word, Registry Number, Name of Substance, and Original Title fields. The search terms were identified through discussion within the research team, scanning background literature, browsing database thesauri and use of the PubMed PubReminer tool (http://hgserver2.amc.nl/cgi-bin/miner/miner2.cgi).

The strategy excluded animal studies from MEDLINE using a standard algorithm (search line 92). The strategy also excluded some ineligible publication types which were unlikely to yield relevant study reports (editorials, news items and case reports) and records with the phrase 'case report' in the title (search line 93).

Reflecting the eligibility criteria, the strategy was restricted to studies published in English language from 2000 to date (search lines 95 to 96).

**Potential Strategy Limitations**

The search strategy was appropriate to the pragmatic review context. It incorporated some pragmatic decisions designed to reduce the number of records retrieved for screening. These decisions were discussed and agreed within the research team. The pragmatic decisions included, for example:

- The terms for the population concepts (search lines 1 to 44) were only designed to retrieve records that explicitly referred to non-specific PIDD, records that explicitly referred to the four specific PIDDs named in the eligibility criteria (i.e. CVID, agammaglobulinemia, hypogammaglobulinemia and SAD), or records that explicitly referred to conditions specified by the research team as falling within these groups (i.e. X-linked agammaglobulinemia, Bruton’s agammaglobulinemia and IgG subclass deficiency only). The research team was aware that some of the eligible conditions were umbrella terms that included further specific eligible subtypes, but it was decided that no further specific condition terms should be included in the search strategy.
- The terms for the non-specific PIDD concept (search lines 1 to 17) were designed to retrieve records that made explicit the 'primary' context. The search was therefore not designed to retrieve records that only referred to immunodeficiency disease without this explicit context.
- The variant search terms included for the non-specific PIDD concept (search lines 1 to 17) text were focused on relatively specific descriptions of the condition. The strategy was not designed, for example, to retrieve records that only referred to, for example, 'low levels of antibodies' or 'decreased levels of immunoglobulin'.
- The eligibility criteria states that the following population is eligible: "patients...with a diagnosis of...PAD (not further specified). This population was added to the eligibility criteria late in the protocol development process. The search strategy was not prospectively designed to retrieve records that referred to a non-specific primary antibody deficiency population. However, the strategy did already include terms that would retrieve records that referred to either 'primary antibody deficiency' (search line 34) or 'PAD' (search line 15). Based on the assumption that studies referring to a non-specific primary antibody deficiency population in an abstract would do so by explicitly referring to either 'primary antibody deficiency', 'PAD' or one of the other terms included in the strategy, the research team decided that no further search development was required.
- The terms for the immunoglobulin therapy concept (search lines 45 to 56, 72 to 87) were designed to retrieve records that referred explicitly to immunoglobulins. The terms did not include 'antibody' as a variant term for immunoglobulin.
- The terms for the immunoglobulin therapy concept (search lines 45 to 56, 72 to 87) were designed to only retrieve records that made the subcutaneous or intravenous administration context explicit.

**Strategy Testing and Peer Review**

The performance of the draft strategy was tested using records for the studies included in four reviews identified by the research team ^1-4^. Across the four reviews, 58 studies were included for which records were available to be found in Ovid MEDLINE. Before date and language limits were applied, the draft strategy successfully retrieved records for all 58 studies.

The final Ovid MEDLINE strategy was peer-reviewed before execution by a second Information Specialist. Peer review considered the appropriateness of the strategy for the review scope and eligibility criteria, inclusion of key search terms, errors in spelling, syntax and line combinations, and application of exclusions.

#### Figure E1: Search strategy for Ovid MEDLINE® ALL

1 primary immunodeficiency diseases/ (1234)

2 immunologic deficiency syndromes/ (15537)

3 ((primary or congenital or familial or hereditary or inherited or inborn) adj3 (immune deficienc* or immuno-deficienc* or immunodeficienc* or immunity deficienc* or immunologic* deficienc*)).ti,ab,kf. (9334)

4 ((primary or congenital or familial or hereditary or inherited or inborn) adj3 (immune defect* or immuno-defect* or immunodefect* or immunity defect* or immunologic* defect*)).ti,ab,kf. (138)

5 ((primary or congenital or familial or hereditary or inherited or inborn) adj3 (immune deficit* or immuno-deficit* or immunodeficit* or immunity deficit* or immunologic* deficit*)).ti,ab,kf. (19)

6 ((primary or congenital or familial or hereditary or inherited or inborn) adj3 (immune depress* or immuno-depress* or immunodepress* or immunity depress* or immunologic* depress*)).ti,ab,kf. (14)

7 ((primary or congenital or familial or hereditary or inherited or inborn) adj3 (immune incompeten* or immuno-incompeten* or immunoincompeten* or immunity incompeten* or immunologic* incompeten*)).ti,ab,kf. (1)

8 ((primary or congenital or familial or hereditary or inherited or inborn) adj3 (immune response depress* or immuno-response depress* or immunoresponse depress* or immunity response depress* or immunologic* response depress*)).ti,ab,kf. (0)

9 ((primary or congenital or familial or hereditary or inherited or inborn) adj3 (immune disorder* or immuno-disorder* or immunodisorder* or immunity disorder* or immunologic* disorder*)).ti,ab,kf. (165)

10 ((primary or congenital or familial or hereditary or inherited or inborn) adj3 (immune dysfunction* or immuno-dysfunction* or immunodysfunction* or immunity dysfunction* or immunologic* dysfunction*)).ti,ab,kf. (37)

11 ((primary or congenital or familial or hereditary or inherited or inborn) adj3 (immune disease* or immuno-disease* or immunodisease* or immunity disease* or immunologic* disease*)).ti,ab,kf. (53)

12 ((primary or congenital or familial or hereditary or inherited or inborn) adj3 (immune suppress* or immuno-suppress* or immunosuppress* or immunity suppress* or immunologic* suppress*)).ti,ab,kf. (949)

13 ((primary or congenital or familial or hereditary or inherited or inborn) adj3 error* adj3 immun*).ti,ab,kf. (974)

14 (pidd or pidds).ti,ab,kf. (292)

15 (pid or pids or pad or pads).ti,ab,kf. (43867)

16 (iei or ieis).ti,ab,kf. (544)

17 or/1-16 (66574)

18 common variable immunodeficiency/ (2534)

19 (common variable or common varied).ti,ab,kf. (3595)

20 (cvid or cvids).ti,ab,kf. (1901)

21 or/18-20 (4210)

22 agammaglobulinemia/ (6778)

23 (agammaglobulinemi* or agammaglobulinaemi*).ti,ab,kf. (2794)

24 (agamma-globulinemi* or agamma-globulinaemi*).ti,ab,kf. (51)

25 (hypogammaglobulinemi* or hypogammaglobulinaemi*).ti,ab,kf. (4481)

26 (hypo-gammaglobulinemi* or hypo-gammaglobulinaemi* or hypogamma-globulinemi* or hypogamma-globulinaemi*).ti,ab,kf. (52)

27 (agammaglobinemi* or agammaglobinaemi*).ti,ab,kf. (2)

28 (agamma-globinemi* or agamma-globinaemi*).ti,ab,kf. (1)

29 (hypogammaglobinemi* or hypogammaglobinaemi*).ti,ab,kf. (43)

30 (hypo-gammaglobinemi* or hypo-gammaglobinaemi* or hypogamma-globinemi* or hypogamma-globinaemi*).ti,ab,kf. (0)

31 (bruton* adj (disease* or disorder* or syndrome*)).ti,ab,kf. (59)

32 (xla or xlas).ti,ab,kf. (777)

33 or/22-32 (9752)

34 ((antibod* or anti-bod*) adj3 deficien*).ti,ab,kf. (2642)

35 ((immunoglobulin* or immuno-globulin* or immuneglobulin* or immune-globulin* or immunoglobin* or immuno-globin* or immuneglobin* or immune-globin* or immune serum globulin* or immune serum globin* or immunogammaglobulin* or immunogammaglobin*) adj6 deficien*).ti,ab,kf. (1883)

36 ((ig or igs) adj6 deficien*).ti,ab,kf. (387)

37 ((allerglobulin* or allerglobin* or gamma-g or gamma-g1 or gamma-g2 or gamma-g2a or gamma-g2b or gamma-g3 or gamma-g4 or gamma globulin* or gammag* or gamma globin* or polyglobulin* or polyglobin*) adj6 deficien*).ti,ab,kf. (248)

38 ((igg or iggs or igg1 or igg2 or igg2a or igg2b or igg3 or igg4 or iggt) adj6 deficien*).ti,ab,kf. (1604)

39 ((ig-g or ig-gs or ig-g1 or ig-g2 or ig-g2a or ig-g2b or ig-g3 or ig-g4 or ig-gt) adj6 deficien*).ti,ab,kf. (29)

40 ((b-cell* or bcell*) adj3 deficien*).ti,ab,kf. (2869)

41 (impaired adj3 polysaccharide respons*).ti,ab,kf. (9)

42 (sad or sads).ti,ab,kf. (13275)

43 or/34-42 (21696)

44 17 or 21 or 33 or 43 (94767)

45 immunization, passive/ (19124)

46 immunoglobulins/ (44741)

47 Immunoglobulin Isotypes/ (4143)

48 immunoglobulin g/ (137758)

49 (immunoglobulin* or immuno-globulin* or immuneglobulin* or immune-globulin* or immunoglobin* or immuno-globin* or immuneglobin* or immune-globin* or immune serum globulin* or immune serum globin* or immunogammaglobulin* or immunogammaglobin*).ti,ab,kf,rn,nm,ot. (394671)

50 (ig or igs).ti,ab,kf,rn,nm,ot. (47190)

51 (allerglobulin* or allerglobin* or gamma-g or gamma-g1 or gamma-g2 or gamma-g2a or gamma-g2b or gamma-g3 or gamma-g4 or gamma globulin* or gammag* or gamma globin* or polyglobulin* or polyglobin*).ti,ab,kf,rn,nm,ot. (31102)

52 (igg or iggs or igg1 or igg2 or igg2a or igg2b or igg3 or igg4 or iggt).ti,ab,kf,rn,nm,ot. (189387)

53 (ig-g or ig-gs or ig-g1 or ig-g2 or ig-g2a or ig-g2b or ig-g3 or ig-g4 or ig-gt).ti,ab,kf,rn,nm,ot. (3378)

54 (immunoreplac* or immun* replac*).ti,ab,kf,rn,nm,ot. (827)

55 (9007-83-4 or 97794-27-9 or 308067-58-5 or 66y330cjhs).ti,ab,kf,rn,nm,ot. (65)

56 or/45-55 (520523)

57 Injections/ (44459)

58 injections, subcutaneous/ or infusions, subcutaneous/ (35248)

59 (subcutaneous* or sub-cutaneous*).ti,ab,kf,rn,nm,ot. (187878)

60 (subcu or sub-cu or subq or sub-q or subcut or sub-cut or subcutanea or sub-cutanea or subcutis or sub-cutis).ti,ab,kf,rn,nm,ot. (3130)

61 (sc or sq).ti,ab,kf,rn,nm,ot. (93947)

62 hypoderm*.ti,ab,kf,rn,nm,ot. (5182)

63 (inject* or infus*).ti,ab,kf,rn,nm,ot. (1085172)

64 or/57-63 (1301535)

65 administration, intravenous/ or infusions, intravenous/ or injections, intravenous/ (148445)

66 intravenous*.ti,ab,kf,rn,nm,ot. (393061)

67 (venous* or vein*).ti,ab,kf,rn,nm,ot. (448395)

68 (drip or drips).ti,ab,kf,rn,nm,ot. (8550)

69 iv.ti,ab,kf,rn,nm,ot. (481530)

70 or/65-69 (1302219)

71 44 and 56 and (64 or 70) (3531)

72 immunoglobulins, intravenous/ (15298)

73 (ivig or ivigs).ti,ab,kf,rn,nm,ot. (9419)

74 (ivigg or iviggs or ivigg1 or ivigg2 or ivigg2a or ivigg2b or ivigg3 or ivigg4 or iviggt).ti,ab,kf,rn,nm,ot. (110)

75 (igiv or igivs).ti,ab,kf,rn,nm,ot. (308)

76 (iggiv or iggsiv or igg1iv or igg2iv or igg2aiv or igg2biv or igg3iv or igg4iv or iggtiv).ti,ab,kf,rn,nm,ot. (0)

77 (ig-giv or ig-gsiv or ig-g1iv or ig-g2iv or ig-g2aiv or ig-g2biv or ig-g3iv or ig-g4iv or ig-gtiv).ti,ab,kf,rn,nm,ot. (0)

78 (scig or scigs).ti,ab,kf,rn,nm,ot. (380)

79 (scigg or sciggs or scigg1 or scigg2 or scigg2a or scigg2b or scigg3 or scigg4 or sciggt).ti,ab,kf,rn,nm,ot. (16)

80 (igsc or igscs).ti,ab,kf,rn,nm,ot. (55)

81 (iggsc or iggssc or igg1sc or igg2sc or igg2asc or igg2bsc or igg3sc or igg4sc or iggtsc).ti,ab,kf,rn,nm,ot. (17)

82 (ig-gsc or ig-gssc or ig-g1sc or ig-g2sc or ig-g2asc or ig-g2bsc or ig-g3sc or ig-g4sc or ig-gtsc).ti,ab,kf,rn,nm,ot. (0)

83 (fscig or fscigs).ti,ab,kf,rn,nm,ot. (29)

84 (fscigg or fsciggs or fscigg1 or fscigg2 or fscigg2a or fscigg2b or fscigg3 or fscigg4 or fsciggt).ti,ab,kf,rn,nm,ot. (0)

85 (igfsc or igsfsc).ti,ab,kf,rn,nm,ot. (0)

86 (iggfsc or iggsfsc or igg1fsc or igg2fsc or igg2afsc or igg2bfsc or igg3fsc or igg4fsc or iggtfsc).ti,ab,kf,rn,nm,ot. (0)

87 (ig-gfsc or ig-gsfsc or ig-g1fsc or ig-g2fsc or ig-g2afsc or ig-g2bfsc or ig-g3fsc or ig-g4fsc or ig-gtfsc).ti,ab,kf,rn,nm,ot. (0)

88 (allerglobuline* or alphaglobin* or asceniv* or baygam* or beriglobin* or biggam* or biogam* or bivigam* or carimune* or citax* or clairyg* or cutaquig* or cuvitru* or endobulin* or flebogamma* or gamafine* or gamastan* or gamimmune* or gamimune* or gamma-16 or gamma-16r or gamma-16tm or gamma16* or gammabulin* or gammagard* or gammagen* or gammaked* or gammaplex* or gammar-p or gammar-pr or gammar-ptm or gammarp* or gammonativ* or gamunex* or globulin-n or globulin-nr or globulin-ntm or globulinn* or globuman* or hizentra* or hyqvia* or intragam* or intraglobin* or iqymune* or iveegam* or kiovig* or norga* or octagam* or panzyga* or polygam* or privigen* or sandoglobulin* or subcuvia* or venimmune* or venogamma* or venoglobulin* or vigam* or vivaglobin* or xembify*).ti,ab,kf,rn,nm,ot. (2032)

89 or/72-88 (20825)

90 44 and 89 (1927)

91 71 or 90 (3626)

92 exp animals/ not humans/ (5089116)

93 (news or editorial or case reports).pt. or case report.ti. (3213962)

94 91 not (92 or 93) (2267)

95 limit 94 to english language (1996)

96 limit 95 to yr="2000 -current" (1403)

Key to Ovid symbols and commands:

* Unlimited right-hand truncation symbol

ti,ab,kf,rn,nm,ot Searches are restricted to the Title (ti), Abstract (ab), Keyword Heading Word (kf), Registry Number/Name of Substance (rn), Name of Substance Word (nm) fields and Original Title (ot) fields

adj Retrieves records that contain terms next to each other (in the shown order)

adjN Retrieves records that contain terms (in any order) within a specified number (N) of words of each other

/ Searches are restricted to the Subject Heading field

exp The subject heading is exploded

pt. Search is restricted to the publication type field

or/1-16 Combines sets 1 to 16 using OR

limit 95 to yr="2000 -current" Limits results to studies published from 2000

### Resources Searched

Literature searches were conducted in the databases and information sources shown in Table E1. The selection of resources reflected the pragmatic review context.

#### Table E1: Databases and information sources searched

| Resource | Interface / URL |
| --- | --- |
| Databases | |
| MEDLINE(R) ALL | OvidSP |
| Cochrane Database of Systematic Reviews (CDSR) | Cochrane Library/Wiley |
| Cochrane Central Register of Controlled Trials (CENTRAL) | Cochrane Library/Wiley |
| HTA Database | https://database.inahta.org/ |
| Other resources | |
| Reference list checking | n/a |

The included studies lists of any retrieved relevant systematic reviews published from 2018 to the date of search were checked for any eligible studies that may have been missed by the database searches.

For each paper that was selected for inclusion in the review, a check was also made to establish if any of the following notices were associated with the included paper: retraction notice, erratum notice, corrected and republished paper notice, expression of concern notice. The check was conducted via the PubMed record for the paper or (if no PubMed record was found) via the journal webpage for the paper. If a relevant notice was associated with an included paper, the notice was assessed by the research team.

### Running the Search Strategies and Downloading Results

Searches were conducted using each database or resource listed above, translating the agreed Ovid MEDLINE strategy appropriately. Translation included consideration of differences in database interfaces and functionality, in addition to variation in indexing languages and thesauri. The final translated database strategies were peer-reviewed by a second Information Specialist. Peer review considered the appropriateness of the translation for the database being searched, errors in syntax and line combinations, and application of exclusions.

The full strategies (including search dates) for all databases searched are shown below (A.1 to A.4).

Results of searches were downloaded in a tagged format and loaded into bibliographic software (EndNote) ^5^. The results were deduplicated using several algorithms and the duplicate references held in a separate EndNote database for checking if required.

1. **Source: MEDLINE ALL**

Interface / URL: OvidSP

Database coverage dates: 1946 to February 02, 2023

Search date: 03/02/2023

Retrieved records: 1403

Search strategy:

1 primary immunodeficiency diseases/ (1234)

2 immunologic deficiency syndromes/ (15537)

3 ((primary or congenital or familial or hereditary or inherited or inborn) adj3 (immune deficienc* or immuno-deficienc* or immunodeficienc* or immunity deficienc* or immunologic* deficienc*)).ti,ab,kf. (9334)

4 ((primary or congenital or familial or hereditary or inherited or inborn) adj3 (immune defect* or immuno-defect* or immunodefect* or immunity defect* or immunologic* defect*)).ti,ab,kf. (138)

5 ((primary or congenital or familial or hereditary or inherited or inborn) adj3 (immune deficit* or immuno-deficit* or immunodeficit* or immunity deficit* or immunologic* deficit*)).ti,ab,kf. (19)

6 ((primary or congenital or familial or hereditary or inherited or inborn) adj3 (immune depress* or immuno-depress* or immunodepress* or immunity depress* or immunologic* depress*)).ti,ab,kf. (14)

7 ((primary or congenital or familial or hereditary or inherited or inborn) adj3 (immune incompeten* or immuno-incompeten* or immunoincompeten* or immunity incompeten* or immunologic* incompeten*)).ti,ab,kf. (1)

8 ((primary or congenital or familial or hereditary or inherited or inborn) adj3 (immune response depress* or immuno-response depress* or immunoresponse depress* or immunity response depress* or immunologic* response depress*)).ti,ab,kf. (0)

9 ((primary or congenital or familial or hereditary or inherited or inborn) adj3 (immune disorder* or immuno-disorder* or immunodisorder* or immunity disorder* or immunologic* disorder*)).ti,ab,kf. (165)

10 ((primary or congenital or familial or hereditary or inherited or inborn) adj3 (immune dysfunction* or immuno-dysfunction* or immunodysfunction* or immunity dysfunction* or immunologic* dysfunction*)).ti,ab,kf. (37)

11 ((primary or congenital or familial or hereditary or inherited or inborn) adj3 (immune disease* or immuno-disease* or immunodisease* or immunity disease* or immunologic* disease*)).ti,ab,kf. (53)

12 ((primary or congenital or familial or hereditary or inherited or inborn) adj3 (immune suppress* or immuno-suppress* or immunosuppress* or immunity suppress* or immunologic* suppress*)).ti,ab,kf. (949)

13 ((primary or congenital or familial or hereditary or inherited or inborn) adj3 error* adj3 immun*).ti,ab,kf. (974)

14 (pidd or pidds).ti,ab,kf. (292)

15 (pid or pids or pad or pads).ti,ab,kf. (43867)

16 (iei or ieis).ti,ab,kf. (544)

17 or/1-16 (66574)

18 common variable immunodeficiency/ (2534)

19 (common variable or common varied).ti,ab,kf. (3595)

20 (cvid or cvids).ti,ab,kf. (1901)

21 or/18-20 (4210)

22 agammaglobulinemia/ (6778)

23 (agammaglobulinemi* or agammaglobulinaemi*).ti,ab,kf. (2794)

24 (agamma-globulinemi* or agamma-globulinaemi*).ti,ab,kf. (51)

25 (hypogammaglobulinemi* or hypogammaglobulinaemi*).ti,ab,kf. (4481)

26 (hypo-gammaglobulinemi* or hypo-gammaglobulinaemi* or hypogamma-globulinemi* or hypogamma-globulinaemi*).ti,ab,kf. (52)

27 (agammaglobinemi* or agammaglobinaemi*).ti,ab,kf. (2)

28 (agamma-globinemi* or agamma-globinaemi*).ti,ab,kf. (1)

29 (hypogammaglobinemi* or hypogammaglobinaemi*).ti,ab,kf. (43)

30 (hypo-gammaglobinemi* or hypo-gammaglobinaemi* or hypogamma-globinemi* or hypogamma-globinaemi*).ti,ab,kf. (0)

31 (bruton* adj (disease* or disorder* or syndrome*)).ti,ab,kf. (59)

32 (xla or xlas).ti,ab,kf. (777)

33 or/22-32 (9752)

34 ((antibod* or anti-bod*) adj3 deficien*).ti,ab,kf. (2642)

35 ((immunoglobulin* or immuno-globulin* or immuneglobulin* or immune-globulin* or immunoglobin* or immuno-globin* or immuneglobin* or immune-globin* or immune serum globulin* or immune serum globin* or immunogammaglobulin* or immunogammaglobin*) adj6 deficien*).ti,ab,kf. (1883)

36 ((ig or igs) adj6 deficien*).ti,ab,kf. (387)

37 ((allerglobulin* or allerglobin* or gamma-g or gamma-g1 or gamma-g2 or gamma-g2a or gamma-g2b or gamma-g3 or gamma-g4 or gamma globulin* or gammag* or gamma globin* or polyglobulin* or polyglobin*) adj6 deficien*).ti,ab,kf. (248)

38 ((igg or iggs or igg1 or igg2 or igg2a or igg2b or igg3 or igg4 or iggt) adj6 deficien*).ti,ab,kf. (1604)

39 ((ig-g or ig-gs or ig-g1 or ig-g2 or ig-g2a or ig-g2b or ig-g3 or ig-g4 or ig-gt) adj6 deficien*).ti,ab,kf. (29)

40 ((b-cell* or bcell*) adj3 deficien*).ti,ab,kf. (2869)

41 (impaired adj3 polysaccharide respons*).ti,ab,kf. (9)

42 (sad or sads).ti,ab,kf. (13275)

43 or/34-42 (21696)

44 17 or 21 or 33 or 43 (94767)

45 immunization, passive/ (19124)

46 immunoglobulins/ (44741)

47 Immunoglobulin Isotypes/ (4143)

48 immunoglobulin g/ (137758)

49 (immunoglobulin* or immuno-globulin* or immuneglobulin* or immune-globulin* or immunoglobin* or immuno-globin* or immuneglobin* or immune-globin* or immune serum globulin* or immune serum globin* or immunogammaglobulin* or immunogammaglobin*).ti,ab,kf,rn,nm,ot. (394671)

50 (ig or igs).ti,ab,kf,rn,nm,ot. (47190)

51 (allerglobulin* or allerglobin* or gamma-g or gamma-g1 or gamma-g2 or gamma-g2a or gamma-g2b or gamma-g3 or gamma-g4 or gamma globulin* or gammag* or gamma globin* or polyglobulin* or polyglobin*).ti,ab,kf,rn,nm,ot. (31102)

52 (igg or iggs or igg1 or igg2 or igg2a or igg2b or igg3 or igg4 or iggt).ti,ab,kf,rn,nm,ot. (189387)

53 (ig-g or ig-gs or ig-g1 or ig-g2 or ig-g2a or ig-g2b or ig-g3 or ig-g4 or ig-gt).ti,ab,kf,rn,nm,ot. (3378)

54 (immunoreplac* or immun* replac*).ti,ab,kf,rn,nm,ot. (827)

55 (9007-83-4 or 97794-27-9 or 308067-58-5 or 66y330cjhs).ti,ab,kf,rn,nm,ot. (65)

56 or/45-55 (520523)

57 Injections/ (44459)

58 injections, subcutaneous/ or infusions, subcutaneous/ (35248)

59 (subcutaneous* or sub-cutaneous*).ti,ab,kf,rn,nm,ot. (187878)

60 (subcu or sub-cu or subq or sub-q or subcut or sub-cut or subcutanea or sub-cutanea or subcutis or sub-cutis).ti,ab,kf,rn,nm,ot. (3130)

61 (sc or sq).ti,ab,kf,rn,nm,ot. (93947)

62 hypoderm*.ti,ab,kf,rn,nm,ot. (5182)

63 (inject* or infus*).ti,ab,kf,rn,nm,ot. (1085172)

64 or/57-63 (1301535)

65 administration, intravenous/ or infusions, intravenous/ or injections, intravenous/ (148445)

66 intravenous*.ti,ab,kf,rn,nm,ot. (393061)

67 (venous* or vein*).ti,ab,kf,rn,nm,ot. (448395)

68 (drip or drips).ti,ab,kf,rn,nm,ot. (8550)

69 iv.ti,ab,kf,rn,nm,ot. (481530)

70 or/65-69 (1302219)

71 44 and 56 and (64 or 70) (3531)

72 immunoglobulins, intravenous/ (15298)

73 (ivig or ivigs).ti,ab,kf,rn,nm,ot. (9419)

74 (ivigg or iviggs or ivigg1 or ivigg2 or ivigg2a or ivigg2b or ivigg3 or ivigg4 or iviggt).ti,ab,kf,rn,nm,ot. (110)

75 (igiv or igivs).ti,ab,kf,rn,nm,ot. (308)

76 (iggiv or iggsiv or igg1iv or igg2iv or igg2aiv or igg2biv or igg3iv or igg4iv or iggtiv).ti,ab,kf,rn,nm,ot. (0)

77 (ig-giv or ig-gsiv or ig-g1iv or ig-g2iv or ig-g2aiv or ig-g2biv or ig-g3iv or ig-g4iv or ig-gtiv).ti,ab,kf,rn,nm,ot. (0)

78 (scig or scigs).ti,ab,kf,rn,nm,ot. (380)

79 (scigg or sciggs or scigg1 or scigg2 or scigg2a or scigg2b or scigg3 or scigg4 or sciggt).ti,ab,kf,rn,nm,ot. (16)

80 (igsc or igscs).ti,ab,kf,rn,nm,ot. (55)

81 (iggsc or iggssc or igg1sc or igg2sc or igg2asc or igg2bsc or igg3sc or igg4sc or iggtsc).ti,ab,kf,rn,nm,ot. (17)

82 (ig-gsc or ig-gssc or ig-g1sc or ig-g2sc or ig-g2asc or ig-g2bsc or ig-g3sc or ig-g4sc or ig-gtsc).ti,ab,kf,rn,nm,ot. (0)

83 (fscig or fscigs).ti,ab,kf,rn,nm,ot. (29)

84 (fscigg or fsciggs or fscigg1 or fscigg2 or fscigg2a or fscigg2b or fscigg3 or fscigg4 or fsciggt).ti,ab,kf,rn,nm,ot. (0)

85 (igfsc or igsfsc).ti,ab,kf,rn,nm,ot. (0)

86 (iggfsc or iggsfsc or igg1fsc or igg2fsc or igg2afsc or igg2bfsc or igg3fsc or igg4fsc or iggtfsc).ti,ab,kf,rn,nm,ot. (0)

87 (ig-gfsc or ig-gsfsc or ig-g1fsc or ig-g2fsc or ig-g2afsc or ig-g2bfsc or ig-g3fsc or ig-g4fsc or ig-gtfsc).ti,ab,kf,rn,nm,ot. (0)

88 (allerglobuline* or alphaglobin* or asceniv* or baygam* or beriglobin* or biggam* or biogam* or bivigam* or carimune* or citax* or clairyg* or cutaquig* or cuvitru* or endobulin* or flebogamma* or gamafine* or gamastan* or gamimmune* or gamimune* or gamma-16 or gamma-16r or gamma-16tm or gamma16* or gammabulin* or gammagard* or gammagen* or gammaked* or gammaplex* or gammar-p or gammar-pr or gammar-ptm or gammarp* or gammonativ* or gamunex* or globulin-n or globulin-nr or globulin-ntm or globulinn* or globuman* or hizentra* or hyqvia* or intragam* or intraglobin* or iqymune* or iveegam* or kiovig* or norga* or octagam* or panzyga* or polygam* or privigen* or sandoglobulin* or subcuvia* or venimmune* or venogamma* or venoglobulin* or vigam* or vivaglobin* or xembify*).ti,ab,kf,rn,nm,ot. (2032)

89 or/72-88 (20825)

90 44 and 89 (1927)

91 71 or 90 (3626)

92 exp animals/ not humans/ (5089116)

93 (news or editorial or case reports).pt. or case report.ti. (3213962)

94 91 not (92 or 93) (2267)

95 limit 94 to english language (1996)

96 limit 95 to yr="2000 -current" (1403)

1. **Source: Cochrane Database of Systematic Reviews (CDSR)**

Interface / URL: Cochrane Library / Wiley

Database coverage dates: Information not found. Issue searched: Issue 2 of 12, February 2023

Search date: 03/02/2023

Retrieved records: 3

Search strategy:

#1 [mh ^"primary immunodeficiency diseases"] 5

#2 [mh ^"immunologic deficiency syndromes"] 613

#3 ((primary or congenital or familial or hereditary or inherited or inborn) near/3 (immune next deficienc* or immuno-deficienc* or immunodeficienc* or immunity next deficienc* or immunologic* next deficienc*)):ti,ab,kw 324

#4 ((primary or congenital or familial or hereditary or inherited or inborn) near/3 (immune next defect* or immuno-defect* or immunodefect* or immunity next defect* or immunologic* next defect*)):ti,ab,kw 0

#5 ((primary or congenital or familial or hereditary or inherited or inborn) near/3 (immune next deficit* or immuno-deficit* or immunodeficit* or immunity next deficit* or immunologic* next deficit*)):ti,ab,kw 0

#6 ((primary or congenital or familial or hereditary or inherited or inborn) near/3 (immune next depress* or immuno-depress* or immunodepress* or immunity next depress* or immunologic* next depress*)):ti,ab,kw 0

#7 ((primary or congenital or familial or hereditary or inherited or inborn) near/3 (immune next incompeten* or immuno-incompeten* or immunoincompeten* or immunity next incompeten* or immunologic* next incompeten*)):ti,ab,kw 0

#8 ((primary or congenital or familial or hereditary or inherited or inborn) near/3 (immune next response next depress* or immuno-response next depress* or immunoresponse next depress* or immunity next response next depress* or immunologic* next response next depress*)):ti,ab,kw 0

#9 ((primary or congenital or familial or hereditary or inherited or inborn) near/3 (immune next disorder* or immuno-disorder* or immunodisorder* or immunity next disorder* or immunologic* next disorder*)):ti,ab,kw 11

#10 ((primary or congenital or familial or hereditary or inherited or inborn) near/3 (immune next dysfunction* or immuno-dysfunction* or immunodysfunction* or immunity next dysfunction* or immunologic* next dysfunction*)):ti,ab,kw 3

#11 ((primary or congenital or familial or hereditary or inherited or inborn) near/3 (immune next disease* or immuno-disease* or immunodisease* or immunity next disease* or immunologic* next disease*)):ti,ab,kw 9

#12 ((primary or congenital or familial or hereditary or inherited or inborn) near/3 (immune next suppress* or immuno-suppress* or immunosuppress* or immunity next suppress* or immunologic* next suppress*)):ti,ab,kw 243

#13 ((primary or congenital or familial or hereditary or inherited or inborn) near/3 error* near/3 immun*):ti,ab,kw 9

#14 (pidd or pidds):ti,ab,kw 14

#15 (pid or pids or pad or pads):ti,ab,kw 7553

#16 (iei or ieis):ti,ab,kw 30

#17 #1 or #2 or #3 or #4 or #5 or #6 or #7 or #8 or #9 or #10 or #11 or #12 or #13 or #14 or #15 or #16 8723

#18 [mh ^"common variable immunodeficiency"] 28

#19 ("common variable" or "common varied"):ti,ab,kw 78

#20 (cvid or cvids):ti,ab,kw 31

#21 #18 or #19 or #20 82

#22 [mh ^"agammaglobulinemia"] 51

#23 (agammaglobulinemi* or agammaglobulinaemi*):ti,ab,kw 117

#24 (agamma-globulinemi* or agamma-globulinaemi*):ti,ab,kw 0

#25 (hypogammaglobulinemi* or hypogammaglobulinaemi*):ti,ab,kw 147

#26 (hypo-gammaglobulinemi* or hypo-gammaglobulinaemi* or hypogamma-globulinemi* or hypogamma-globulinaemi*):ti,ab,kw 0

#27 (agammaglobinemi* or agammaglobinaemi*):ti,ab,kw 0

#28 (agamma-globinemi* or agamma-globinaemi*):ti,ab,kw 0

#29 (hypogammaglobinemi* or hypogammaglobinaemi*):ti,ab,kw 0

#30 (hypo-gammaglobinemi* or hypo-gammaglobinaemi* or hypogamma-globinemi* or hypogamma-globinaemi*):ti,ab,kw 0

#31 (bruton* next (disease* or disorder* or syndrome*)):ti,ab,kw 1

#32 (xla or xlas):ti,ab,kw 10

#33 #22 or #23 or #24 or #25 or #26 or #27 or #28 or #29 or #30 or #31 or #32 234

#34 ((antibod* or anti-bod*) near/3 deficien*):ti,ab,kw 78

#35 ((immunoglobulin* or immuno-globulin* or immuneglobulin* or immune-globulin* or immunoglobin* or immuno-globin* or immuneglobin* or immune-globin* or immune next serum next globulin* or immune next serum next globin* or immunogammaglobulin* or immunogammaglobin*) near/6 deficien*):ti,ab,kw 189

#36 ((ig or igs) near/6 deficien*):ti,ab,kw 143

#37 ((allerglobulin* or allerglobin* or gamma-g or gamma-g1 or gamma-g2 or gamma-g2a or gamma-g2b or gamma-g3 or gamma-g4 or gamma next globulin* or gammag* or gamma next globin* or polyglobulin* or polyglobin*) near/6 deficien*):ti,ab,kw 23

#38 ((igg or iggs or igg1 or igg2 or igg2a or igg2b or igg3 or igg4 or iggt) near/6 deficien*):ti,ab,kw 46

#39 ((ig-g or ig-gs or ig-g1 or ig-g2 or ig-g2a or ig-g2b or ig-g3 or ig-g4 or ig-gt) near/6 deficien*):ti,ab,kw 51

#40 ((b-cell* or bcell*) near/3 deficien*):ti,ab,kw 7

#41 (impaired near/3 polysaccharide next respons*):ti,ab,kw 0

#42 (sad or sads):ti,ab,kw 3056

#43 #34 or #35 or #36 or #37 or #38 or #39 or #40 or #41 or #42 3418

#44 #17 or #21 or #33 or #43 12237

#45 [mh ^"immunization, passive"] 496

#46 [mh ^"immunoglobulins"] 1414

#47 [mh ^"Immunoglobulin Isotypes"] 45

#48 [mh ^"immunoglobulin g"] 3249

#49 (immunoglobulin* or immuno-globulin* or immuneglobulin* or immune-globulin* or immunoglobin* or immuno-globin* or immuneglobin* or immune-globin* or immune next serum next globulin* or immune next serum next globin* or immunogammaglobulin* or immunogammaglobin*):ti,ab,kw 16378

#50 (ig or igs):ti,ab,kw 19757

#51 (allerglobulin* or allerglobin* or gamma-g or gamma-g1 or gamma-g2 or gamma-g2a or gamma-g2b or gamma-g3 or gamma-g4 or gamma next globulin* or gammag* or gamma next globin* or polyglobulin* or polyglobin*):ti,ab,kw 2202

#52 (igg or iggs or igg1 or igg2 or igg2a or igg2b or igg3 or igg4 or iggt):ti,ab,kw 8478

#53 (ig-g or ig-gs or ig-g1 or ig-g2 or ig-g2a or ig-g2b or ig-g3 or ig-g4 or ig-gt):ti,ab,kw 9045

#54 (immunoreplac* or immun* next replac*):ti,ab,kw 37

#55 ("9007-83-4" or "97794-27-9" or "308067-58-5" or 66y330cjhs):ti,ab,kw 10

#56 #45 or #46 or #47 or #48 or #49 or #50 or #51 or #52 or #53 or #54 or #55 30172

#57 [mh ^"Injections"] 2988

#58 [mh ^"injections, subcutaneous"] or [mh ^"infusions, subcutaneous"] 4353

#59 (subcutaneous* or sub-cutaneous*):ti,ab,kw 36187

#60 (subcu or sub-cu or subq or sub-q or subcut or sub-cut or subcutanea or sub-cutanea or subcutis or sub-cutis):ti,ab,kw 318

#61 (sc or sq):ti,ab,kw 17392

#62 hypoderm*:ti,ab,kw 242

#63 (inject* or infus*):ti,ab,kw 183595

#64 #57 or #58 or #59 or #60 or #61 or #62 or #63 208698

#65 [mh ^"administration, intravenous"] or [mh ^"infusions, intravenous"] or [mh ^"injections, intravenous"] 20324

#66 intravenous*:ti,ab,kw 106859

#67 (venous* or vein*):ti,ab,kw 47222

#68 (drip or drips):ti,ab,kw 1771

#69 iv:ti,ab,kw 88917

#70 #65 or #66 or #67 or #68 or #69 203500

#71 #44 and #56 and (#64 or #70) 372

#72 [mh ^"immunoglobulins, intravenous"] 1039

#73 (ivig or ivigs):ti,ab,kw 1574

#74 (ivigg or iviggs or ivigg1 or ivigg2 or ivigg2a or ivigg2b or ivigg3 or ivigg4 or iviggt):ti,ab,kw 19

#75 (igiv or igivs):ti,ab,kw 144

#76 (iggiv or iggsiv or igg1iv or igg2iv or igg2aiv or igg2biv or igg3iv or igg4iv or iggtiv):ti,ab,kw 1

#77 (ig-giv or ig-gsiv or ig-g1iv or ig-g2iv or ig-g2aiv or ig-g2biv or ig-g3iv or ig-g4iv or ig-gtiv):ti,ab,kw 1

#78 (scig or scigs):ti,ab,kw 90

#79 (scigg or sciggs or scigg1 or scigg2 or scigg2a or scigg2b or scigg3 or scigg4 or sciggt):ti,ab,kw 1

#80 (igsc or igscs):ti,ab,kw 9

#81 (iggsc or iggssc or igg1sc or igg2sc or igg2asc or igg2bsc or igg3sc or igg4sc or iggtsc):ti,ab,kw 3

#82 (ig-gsc or ig-gssc or ig-g1sc or ig-g2sc or ig-g2asc or ig-g2bsc or ig-g3sc or ig-g4sc or ig-gtsc):ti,ab,kw 0

#83 (fscig or fscigs):ti,ab,kw 8

#84 (fscigg or fsciggs or fscigg1 or fscigg2 or fscigg2a or fscigg2b or fscigg3 or fscigg4 or fsciggt):ti,ab,kw 0

#85 (igfsc or igsfsc):ti,ab,kw 0

#86 (iggfsc or iggsfsc or igg1fsc or igg2fsc or igg2afsc or igg2bfsc or igg3fsc or igg4fsc or iggtfsc):ti,ab,kw 0

#87 (ig-gfsc or ig-gsfsc or ig-g1fsc or ig-g2fsc or ig-g2afsc or ig-g2bfsc or ig-g3fsc or ig-g4fsc or ig-gtfsc):ti,ab,kw 0

#88 (allerglobuline* or alphaglobin* or asceniv* or baygam* or beriglobin* or biggam* or biogam* or bivigam* or carimune* or citax* or clairyg* or cutaquig* or cuvitru* or endobulin* or flebogamma* or gamafine* or gamastan* or gamimmune* or gamimune* or gamma-16 or gamma-16r or gamma-16tm or gamma16* or gammabulin* or gammagard* or gammagen* or gammaked* or gammaplex* or gammar-p or gammar-pr or gammar-ptm or gammarp* or gammonativ* or gamunex* or globulin-n or globulin-nr or globulin-ntm or globulinn* or globuman* or hizentra* or hyqvia* or intragam* or intraglobin* or iqymune* or iveegam* or kiovig* or norga* or octagam* or panzyga* or polygam* or privigen* or sandoglobulin* or subcuvia* or venimmune* or venogamma* or venoglobulin* or vigam* or vivaglobin* or xembify*):ti,ab,kw 484

#89 #72 or #73 or #74 or #75 or #76 or #77 or #78 or #79 or #80 or #81 or #82 or #83 or #84 or #85 or #86 or #87 or #88 2336

#90 #44 and #89 180

#91 #71 or #90 with Cochrane Library publication date Between Jan 2000 and Dec 2023, in Cochrane Reviews, Cochrane Protocols 3

1. **Source: Cochrane Central Register of Controlled Trials (CENTRAL)**

Interface / URL: Cochrane Library / Wiley

Database coverage dates: Information not found. Issue searched: Issue 2 of 12, February 2023

Search date: 03/02/2023

Retrieved records: 314

Search strategy:

#1 [mh ^"primary immunodeficiency diseases"] 5

#2 [mh ^"immunologic deficiency syndromes"] 613

#3 ((primary or congenital or familial or hereditary or inherited or inborn) near/3 (immune next deficienc* or immuno-deficienc* or immunodeficienc* or immunity next deficienc* or immunologic* next deficienc*)) 363

#4 ((primary or congenital or familial or hereditary or inherited or inborn) near/3 (immune next defect* or immuno-defect* or immunodefect* or immunity next defect* or immunologic* next defect*)) 1

#5 ((primary or congenital or familial or hereditary or inherited or inborn) near/3 (immune next deficit* or immuno-deficit* or immunodeficit* or immunity next deficit* or immunologic* next deficit*)) 0

#6 ((primary or congenital or familial or hereditary or inherited or inborn) near/3 (immune next depress* or immuno-depress* or immunodepress* or immunity next depress* or immunologic* next depress*)) 0

#7 ((primary or congenital or familial or hereditary or inherited or inborn) near/3 (immune next incompeten* or immuno-incompeten* or immunoincompeten* or immunity next incompeten* or immunologic* next incompeten*)) 0

#8 ((primary or congenital or familial or hereditary or inherited or inborn) near/3 (immune next response next depress* or immuno-response next depress* or immunoresponse next depress* or immunity next response next depress* or immunologic* next response next depress*)) 0

#9 ((primary or congenital or familial or hereditary or inherited or inborn) near/3 (immune next disorder* or immuno-disorder* or immunodisorder* or immunity next disorder* or immunologic* next disorder*)) 14

#10 ((primary or congenital or familial or hereditary or inherited or inborn) near/3 (immune next dysfunction* or immuno-dysfunction* or immunodysfunction* or immunity next dysfunction* or immunologic* next dysfunction*)) 3

#11 ((primary or congenital or familial or hereditary or inherited or inborn) near/3 (immune next disease* or immuno-disease* or immunodisease* or immunity next disease* or immunologic* next disease*)) 12

#12 ((primary or congenital or familial or hereditary or inherited or inborn) near/3 (immune next suppress* or immuno-suppress* or immunosuppress* or immunity next suppress* or immunologic* next suppress*)) 264

#13 ((primary or congenital or familial or hereditary or inherited or inborn) near/3 error* near/3 immun*) 14

#14 (pidd or pidds) 21

#15 (pid or pids or pad or pads) 8116

#16 (iei or ieis) 33

#17 #1 or #2 or #3 or #4 or #5 or #6 or #7 or #8 or #9 or #10 or #11 or #12 or #13 or #14 or #15 or #16 9359

#18 [mh ^"common variable immunodeficiency"] 28

#19 ("common variable" or "common varied") 83

#20 (cvid or cvids) 31

#21 #18 or #19 or #20 87

#22 [mh ^"agammaglobulinemia"] 51

#23 (agammaglobulinemi* or agammaglobulinaemi*) 121

#24 (agamma-globulinemi* or agamma-globulinaemi*) 0

#25 (hypogammaglobulinemi* or hypogammaglobulinaemi*) 155

#26 (hypo-gammaglobulinemi* or hypo-gammaglobulinaemi* or hypogamma-globulinemi* or hypogamma-globulinaemi*) 0

#27 (agammaglobinemi* or agammaglobinaemi*) 0

#28 (agamma-globinemi* or agamma-globinaemi*) 0

#29 (hypogammaglobinemi* or hypogammaglobinaemi*) 0

#30 (hypo-gammaglobinemi* or hypo-gammaglobinaemi* or hypogamma-globinemi* or hypogamma-globinaemi*) 0

#31 (bruton* next (disease* or disorder* or syndrome*)) 1

#32 (xla or xlas) 10

#33 #22 or #23 or #24 or #25 or #26 or #27 or #28 or #29 or #30 or #31 or #32 246

#34 ((antibod* or anti-bod*) near/3 deficien*) 88

#35 ((immunoglobulin* or immuno-globulin* or immuneglobulin* or immune-globulin* or immunoglobin* or immuno-globin* or immuneglobin* or immune-globin* or immune next serum next globulin* or immune next serum next globin* or immunogammaglobulin* or immunogammaglobin*) near/6 deficien*) 249

#36 ((ig or igs) near/6 deficien*) 151

#37 ((allerglobulin* or allerglobin* or gamma-g or gamma-g1 or gamma-g2 or gamma-g2a or gamma-g2b or gamma-g3 or gamma-g4 or gamma next globulin* or gammag* or gamma next globin* or polyglobulin* or polyglobin*) near/6 deficien*) 35

#38 ((igg or iggs or igg1 or igg2 or igg2a or igg2b or igg3 or igg4 or iggt) near/6 deficien*) 48

#39 ((ig-g or ig-gs or ig-g1 or ig-g2 or ig-g2a or ig-g2b or ig-g3 or ig-g4 or ig-gt) near/6 deficien*) 53

#40 ((b-cell* or bcell*) near/3 deficien*) 7

#41 (impaired near/3 polysaccharide next respons*) 0

#42 (sad or sads) 3321

#43 #34 or #35 or #36 or #37 or #38 or #39 or #40 or #41 or #42 3753

#44 #17 or #21 or #33 or #43 13165

#45 [mh ^"immunization, passive"] 496

#46 [mh ^"immunoglobulins"] 1414

#47 [mh ^"Immunoglobulin Isotypes"] 45

#48 [mh ^"immunoglobulin g"] 3249

#49 (immunoglobulin* or immuno-globulin* or immuneglobulin* or immune-globulin* or immunoglobin* or immuno-globin* or immuneglobin* or immune-globin* or immune next serum next globulin* or immune next serum next globin* or immunogammaglobulin* or immunogammaglobin*) 16776

#50 (ig or igs) 21663

#51 (allerglobulin* or allerglobin* or gamma-g or gamma-g1 or gamma-g2 or gamma-g2a or gamma-g2b or gamma-g3 or gamma-g4 or gamma next globulin* or gammag* or gamma next globin* or polyglobulin* or polyglobin*) 2332

#52 (igg or iggs or igg1 or igg2 or igg2a or igg2b or igg3 or igg4 or iggt) 8716

#53 (ig-g or ig-gs or ig-g1 or ig-g2 or ig-g2a or ig-g2b or ig-g3 or ig-g4 or ig-gt) 9283

#54 (immunoreplac* or immun* next replac*) 42

#55 ("9007-83-4" or "97794-27-9" or "308067-58-5" or 66y330cjhs) 10

#56 #45 or #46 or #47 or #48 or #49 or #50 or #51 or #52 or #53 or #54 or #55 32284

#57 [mh ^"Injections"] 2988

#58 [mh ^"injections, subcutaneous"] or [mh ^"infusions, subcutaneous"] 4353

#59 (subcutaneous* or sub-cutaneous*) 38061

#60 (subcu or sub-cu or subq or sub-q or subcut or sub-cut or subcutanea or sub-cutanea or subcutis or sub-cutis) 430

#61 (sc or sq) 32297

#62 hypoderm* 258

#63 (inject* or infus*) 187211

#64 #57 or #58 or #59 or #60 or #61 or #62 or #63 226224

#65 [mh ^"administration, intravenous"] or [mh ^"infusions, intravenous"] or [mh ^"injections, intravenous"] 20324

#66 intravenous* 110274

#67 (venous* or vein*) 48834

#68 (drip or drips) 1842

#69 iv 94861

#70 #65 or #66 or #67 or #68 or #69 211536

#71 #44 and #56 and (#64 or #70) 455

#72 [mh ^"immunoglobulins, intravenous"] 1039

#73 (ivig or ivigs) 1647

#74 (ivigg or iviggs or ivigg1 or ivigg2 or ivigg2a or ivigg2b or ivigg3 or ivigg4 or iviggt) 20

#75 (igiv or igivs) 150

#76 (iggiv or iggsiv or igg1iv or igg2iv or igg2aiv or igg2biv or igg3iv or igg4iv or iggtiv) 1

#77 (ig-giv or ig-gsiv or ig-g1iv or ig-g2iv or ig-g2aiv or ig-g2biv or ig-g3iv or ig-g4iv or ig-gtiv) 1

#78 (scig or scigs) 92

#79 (scigg or sciggs or scigg1 or scigg2 or scigg2a or scigg2b or scigg3 or scigg4 or sciggt) 2

#80 (igsc or igscs) 10

#81 (iggsc or iggssc or igg1sc or igg2sc or igg2asc or igg2bsc or igg3sc or igg4sc or iggtsc) 3

#82 (ig-gsc or ig-gssc or ig-g1sc or ig-g2sc or ig-g2asc or ig-g2bsc or ig-g3sc or ig-g4sc or ig-gtsc) 0

#83 (fscig or fscigs) 8

#84 (fscigg or fsciggs or fscigg1 or fscigg2 or fscigg2a or fscigg2b or fscigg3 or fscigg4 or fsciggt) 0

#85 (igfsc or igsfsc) 0

#86 (iggfsc or iggsfsc or igg1fsc or igg2fsc or igg2afsc or igg2bfsc or igg3fsc or igg4fsc or iggtfsc) 0

#87 (ig-gfsc or ig-gsfsc or ig-g1fsc or ig-g2fsc or ig-g2afsc or ig-g2bfsc or ig-g3fsc or ig-g4fsc or ig-gtfsc) 0

#88 (allerglobuline* or alphaglobin* or asceniv* or baygam* or beriglobin* or biggam* or biogam* or bivigam* or carimune* or citax* or clairyg* or cutaquig* or cuvitru* or endobulin* or flebogamma* or gamafine* or gamastan* or gamimmune* or gamimune* or gamma-16 or gamma-16r or gamma-16tm or gamma16* or gammabulin* or gammagard* or gammagen* or gammaked* or gammaplex* or gammar-p or gammar-pr or gammar-ptm or gammarp* or gammonativ* or gamunex* or globulin-n or globulin-nr or globulin-ntm or globulinn* or globuman* or hizentra* or hyqvia* or intragam* or intraglobin* or iqymune* or iveegam* or kiovig* or norga* or octagam* or panzyga* or polygam* or privigen* or sandoglobulin* or subcuvia* or venimmune* or venogamma* or venoglobulin* or vigam* or vivaglobin* or xembify*) 883

#89 #72 or #73 or #74 or #75 or #76 or #77 or #78 or #79 or #80 or #81 or #82 or #83 or #84 or #85 or #86 or #87 or #88 2777

#90 #44 and #89 207

#91 #71 or #90 with Publication Year from 2000 to 2023, in Trials 314

1. **Source: HTA database**

Interface / URL: https://database.inahta.org/

Database coverage dates: Information not found. The former database was produced by the CRD until March 2018, at which time the addition of records was stopped as INAHTA was in the process of rebuilding the new database platform. In July 2019, the database records were exported from the CRD platform and imported into the new platform that was developed by INAHTA. The rebuild of the new platform was launched in June 2020.

Search date: 03/02/2023

Retrieved records: 47

Search strategy:

1 "primary immunodeficiency diseases"[mh] 1

2 "immunologic deficiency syndromes"[mh] 6

3 (primary or congenital or familial or hereditary or inherited or inborn) and immun* 112

4 pidd or pidds or pid or pids or pad or pads or iei or ieis 46

5 "common variable immunodeficiency"[mh] 0

6 "common variable" or "common varied" 2

7 cvid or cvids 0

8 "agammaglobulinemia"[mh] 3

9 agammaglobulinemi* or agammaglobulinaemi* 2

10 agamma and (globulinemi* or globulinaemi* or globinemi* or globinaemi*) 0

11 hypogammaglobulinemi* or hypogammaglobulinaemi* 6

12 hypo and (gammaglobulinemi* or gammaglobulinaemi* or gammaglobinemi* or gammaglobinaemi*) 0

13 hypogamma and (globulinemi* or globulinaemi* or globinemi* or globinaemi*) 0

14 agammaglobinemi* or agammaglobinaemi* 0

15 hypogammaglobinemi* or hypogammaglobinaemi* 0

16 bruton* 4

17 xla or xlas 1

18 (antibod* or anti-body or anti-bodies) and deficien* 12

19 immun* and deficien* 26

20 (ig or igs) and deficien* 2

21 (allerglobulin* or allerglobin* or gamma-g or gamma-g1 or gamma-g2 or gamma-g2a or gamma-g2b or gamma-g3 or gamma-g4 or (gamma and globulin*) or gammag* or (gamma and globin*) or polyglobulin* or polyglobin*) and deficien* 0

22 (igg or iggs or igg1 or igg2 or igg2a or igg2b or igg3 or igg4 or iggt) and deficien* 4

23 (ig-g or ig-gs or ig-g1 or ig-g2 or ig-g2a or ig-g2b or ig-g3 or ig-g4 or ig-gt) and deficien* 0

24 (b-cell or b-cells or bcell*) and deficien* 26

25 impaired and polysaccharide and respons* 0

26 sad or sads 3

27 #26 OR #25 OR #24 OR #23 OR #22 OR #21 OR #20 OR #19 OR #18 OR #17 OR #16 OR #15 OR #14 OR #13 OR #12 OR #11 OR #10 OR #9 OR #8 OR #7 OR #6 OR #5 OR #4 OR #3 OR #2 OR #1 203

28 "immunization, passive"[mh] 4

29 "immunoglobulins"[mh] 61

30 "immunoglobulin isotypes"[mh] 0

31 "immunoglobulin g"[mh] 40

32 immunoglobulin* or immuneglobulin* or immunoglobin* or immuneglobin* or immunogammaglobulin* or immunogammaglobin* or (immun* and (globin* or globulin*)) 107

33 ig or igs 10

34 allerglobulin* or allerglobin* or gamma-g or gamma-g1 or gamma-g2 or gamma-g2a or gamma-g2b or gamma-g3 or gamma-g4 or (gamma and globulin*) or gammag* or (gamma and globin*) or polyglobulin* or polyglobin* 2

35 igg or iggs or igg1 or igg2 or igg2a or igg2b or igg3 or igg4 or iggt 17

36 ig-g or ig-gs or ig-g1 or ig-g2 or ig-g2a or ig-g2b or ig-g3 or ig-g4 or ig-gt 0

37 immunoreplac* or (immun* and replac*) 25

38 9007-83-4 or 97794-27-9 or 308067-58-5 or 66y330cjhs 0

39 #38 OR #37 OR #36 OR #35 OR #34 OR #33 OR #32 OR #31 OR #30 OR #29 OR #28 176

40 #39 AND #27 48

41 "immunoglobulins, intravenous"[mh] 56

42 ivig or ivigs or ivigg or iviggs or ivigg1 or ivigg2 or ivigg2a or ivigg2b or ivigg3 or ivigg4 or iviggt or igiv or igivs or iggiv or iggsiv or igg1iv or igg2iv or igg2aiv or igg2biv or igg3iv or igg4iv or iggtiv or ig-giv or ig-gsiv or ig-g1iv or ig-g2iv or ig-g2aiv or ig-g2biv or ig-g3iv or ig-g4iv or ig-gtiv or scig or scigs or scigg or sciggs or scigg1 or scigg2 or scigg2a or scigg2b or scigg3 or scigg4 or sciggt or igsc or igscs or iggsc or iggssc or igg1sc or igg2sc or igg2asc or igg2bsc or igg3sc or igg4sc or iggtsc or ig-gsc or ig-gssc or ig-g1sc or ig-g2sc or ig-g2asc or ig-g2bsc or ig-g3sc or ig-g4sc or ig-gtsc or fscig or fscigs or fscigg or fsciggs or fscigg1 or fscigg2 or fscigg2a or fscigg2b or fscigg3 or fscigg4 or fsciggt or igfsc or igsfsc or iggfsc or iggsfsc or igg1fsc or igg2fsc or igg2afsc or igg2bfsc or igg3fsc or igg4fsc or iggtfsc or ig-gfsc or ig-gsfsc or ig-g1fsc or ig-g2fsc or ig-g2afsc or ig-g2bfsc or ig-g3fsc or ig-g4fsc or ig-gtfsc 44

43 allerglobuline* or alphaglobin* or asceniv* or baygam* or beriglobin* or biggam* or biogam* or bivigam* or carimune* or citax* or clairyg* or cutaquig* or cuvitru* or endobulin* or flebogamma* or gamafine* or gamastan* or gamimmune* or gamimune* or gamma-16 or gamma-16r or gamma-16tm or gamma16* or gammabulin* or gammagard* or gammagen* or gammaked* or gammaplex* or gammar-p or gammar-pr or gammar-ptm or gammarp* or gammonativ* or gamunex* or globulin-n or globulin-nr or globulin-ntm or globulinn* or globuman* or hizentra* or hyqvia* or intragam* or intraglobin* or iqymune* or iveegam* or kiovig* or norga* or octagam* or panzyga* or polygam* or privigen* or sandoglobulin* or subcuvia* or venimmune* or venogamma* or venoglobulin* or vigam* or vivaglobin* or xembify* 2

44 #43 OR #42 OR #41 70

45 #44 AND #27 31

46 #45 OR #40 48

47 * FROM 2000 TO 2023 19163

48 #47 AND #46 47

Search note:

Post search and assessment of results, an issue with how the HTA Database search interface deals with search terms that contain less than 3 characters was identified. It was identified that the interface will not search on such terms when they are stand-alone terms, or when a number of such terms form a phrase together, and does not appear to search on them when they precede a term with three or more characters as part of a phrase. The interface does appear to search on them when they follow a term with three or more characters as part of a phrase.

The strategy was checked to see if adjusting it to take account of the above would have retrieved any additional results. It was established that no additional results would have been retrieved.

### Data Extraction Elements

Study methods:

- Study design.
- Location of study and number of sites.
- Date of study and patient recruitment.
- Outcomes.
- Data collection timepoints.
- Subgroups:
  - Adult/child.
  - Pre-existing illness (e.g. bronchiectasis).
  - Dose.
  - Regimen.
  - Trough level.
- List of ineligible subgroups (subgroups other than those listed).
- Duration of Ig therapy.
- Duration of follow-up.
- Details of statistical analysis (including number of patients included/analysed, withdrawals and discontinuations, methods used to account for missing data, etc.).

Intervention details:

- Brand name and manufacturer.
- Infusion route.
- Mean dose.
- Regimen/treatment interval.
- Mean number of infusions.
- Details of infusion procedure (e.g. manual vs pump-assisted).
- No. of infusion sites.
- Mean trough Ig level at baseline.
- Pre-treatments ahead of Ig infusion.
- Concomitant medications.
- Prophylactic antibiotic use.

Population details:

- Key inclusion criteria.
- Key exclusion criteria.
- PIDDs diagnosis.
- Diagnostic criteria.
- Age (including age group adults/children/mixed).
- Gender.
- Race or ethnic group.
- Weight.
- Pre-existing illness.
- Previous Ig dose.

Outcomes:

- Ig trough levels.
- Serious bacterial infection (SBI) and overall infection rate.
- Hospitalizations.
- Antibiotic use.
- School/work days missed.
- HRQoL.
- Economic outcomes.
- Overall safety outcomes:
  - The overall number of adverse events reported as serious, general, treatment-related and local or systemic.
  - General or non-serious events further specified by severity (e.g. mild/moderate/severe) were not extracted.
  - Specific adverse events (e.g. headache, fatigue, hypotension) apart from local infusion reactions were not extracted.

## Data Tables

#### Table E2: Included study characteristics

| **Study reference** | **Details of funding** | **Study design** | **Number of sites and location** | **Date of patient recruitment (or date of study)** | **Outcomes assessed** | **Duration of study Ig exposure** | **Patient withdrawals/exclusion following randomization** |
| --- | --- | --- | --- | --- | --- | --- | --- |
| **Between-person comparative (9 studies)** | | | | | | | |
| Anderson 2021 ^6^ | Funding from CSL Behring | Prospective cohort study | 12 sites in the USA and Canada | February 1^st^ 2017 to December 14^th^ 2018 | Ig levels AE | 12 to 16 weeks  Follow-up up to 17 weeks per participant | Rapid push: 1 AE in volume cohort,  Flow-rate cohort: 1 withdrawal  Manual push cohort 1 AE and 1 protocol deviation). |
| Bienvenu 2018 ^7^ | Sponsored by Octapharma | Crossover RCT | 6 sites in France | NR | Infection rate Antibiotic use  AE Patient satisfaction  HRQoL | 6 months | 1 discontinued due to AE, 1 withdrew |
| Chapel 2000 ^8^ | The study was conducted with the financial support of Immuno, Vienna (now Baxter Hyland Immuno) and a Biomed. 2 PL 963007 grant. | Crossover RCT | 3 sites in the UK and Sweden | June 1994 to April 1996 | AE leading to discontinuation (only outcome reported at point of crossover) | 12 months | 1 withdrew due to systemic AE |
| Desai 2009 ^9^ | Supported by a grant from Talecris Biotherapeutics, Inc | Crossover RCT | USA | NR | Ig levels Infection rate AE Treatment preference | 12 months | 1 withdrew due to pregnancy. |
| Matamoros 2005 ^10^ | NR | RCT | 3 sites in Spain | NR | Ig levels (only outcome for which PIDs subgroup reported) | 16 months | 33/35 completed the study. reasons for discontinuation NR |
| Roifman 2003 ^11^ | Funded by Bayer Healthcare | RCT | 25 sites in the USA and Canada | NR (Study: March 1999 to June 2000) | Infection rate Lung function AE | 9 months | IGIV-C: 13 protocol violations (due to shorter treatment period, or to fluctuation of IVIG dose prior to study entry), 1 noncompliant  IGIV-SD: 11 protocol violations (due to shorter treatment period, or to fluctuation of IVIG dose prior to study entry), 1 noncompliant |
| Warnatz 2022 ^12^ | The study was funded by Octapharma | Crossover RCT | 12 sites in Australia, Germany, Italy, UK | July 2015 to June 2017 | Ig levels Infection rate Hospitalizations Patient satisfaction HRQoL (LQI, TSQM) Antibiotic use Costs AE | 6 months | 1 excluded from the ITT population because LQI data at V2 and V3 were missing).  4 excluded from the PP population due to missing data. Of those, 2prematurely withdrew from the study, one was lost to follow-up and another one prematurely discontinued due to an AE. |
| Wasserman 2017 ^13^ | Funded by Bio Products Laboratory | Crossover RCT | 16 sites in the USA, UK and Hungary | NR | Ig levels AEs | Range: 38 to 48 weeks for adults patient and 23 to 28 weeks for pediatrics patient | 2 (1 adult withdrew consent, 1 child was withdrawn at investigator discretion) |
| Wolf 2003 ^14^ | NR | RCT | NR | NR | Ig levels School/work days missed Antibiotic use Hospitalizations Patient wellbeing AE | 6 months | Not explicitly reported,  1 patient appears to have withdrawn from IVIG-N arm and 2 from Sandoglobulin arm |
| **Within-person comparative (12 studies)** | | | | | | | |
| Bjorkander 2006 ^15^ | Supported by Baxter AG, Vienna, Austria | Prospective before-after | 6 sites in Sweden and Finland | NR | Infection rate Antibiotic use School/work days absent AEs | 36 weeks | 1 (withdrew, patient developed B-cell lymphoma). |
| Bleasel 2012 ^16^ | This research was supported and funded by CSL Limited | Prospective before-after | 4 sites in Australia | NR | Ig levels Infection rates AEs | 28 to 36 weeks  Last follow-up at least 30 days post the last dose | 0 |
| Borte 2017 ^17^ | Funded by Baxalta (now part of Shire) | Prospective before-after | 16 sites in Austria, Germany, Hungary, Sweden, United Kingdom (NCT01412385) | NR | Fever episodes Ig levels Physician visits due to illness/infection HRQoL (LQI, EQ-5D, PEDS-QL, SF-36) Infection rate School/work days missed Hospitalizations AEs | 64 weeks total (period 1: 12 to 13 weeks; period 2: 52 weeks) | Period 1: 1 withdrew prematurely during period 1 after becoming pregnant.  Period 2: 3 (1 reported pain during and after administration and chose to stop participation and 2 withdrew consent for reasons unrelated to an AE) |
| Heimall 2016 ^18^ | Sponsored by Grifols Therapeutics Inc | Prospective before-after | 4 sites in the USA | March 2012 to October 2013 | Ig levels Infection rates AEs | Mean patient exposure 14.20 weeks for the run-in phase, 4.50 weeks for the IV phase, and 11.03 weeks for the SC phase. | 3 (1 discontinued due to AE post-screening, 1 withdrew during run-in phase due to protocol violation, 1 withdrew during SCIG phase due to pain at injection site) |
| Kallenberg 2007 ^19^ | This research was funded by Baxter. | Prospective before-after | 1 site in the Netherlands | NR | Ig levels Pharmacoeconomic data AEs | 28 infusions | 1 (missing infusion times) |
| Niebur 2015 ^20^ | Funding was provided by a grant from CSL Behring, LLC | Prospective before-after | 1 sites in the USA | NR | Ig levels Infection rates Antibiotic use School/work days missed Acute medial visits  Hospitalizations AE Treatment satisfaction | 32 weeks | NR |
| Sleasman 2019 ^21^ | Supported by Grifols, Research Triangle Park, NC, USA | Prospective before-after | 20 sites in the USA and Canada | 4 January 2016 and 14 December 2017 | Ig levels Infection rates AEs Antibiotic use Hospitalization School/work days missed | 28 weeks | 11 (5 due to AEs, 4 withdrew, 1 lost to follow-up,1 other reason) |
| Suez 2016 ^22^ | Funded by Baxalta (now part of Shire) | Prospective before-after | 15 sites in the USA and Canada | NR | Infection rates Fever episodes Physician visits due to illness/infection School/work days missed Hospitalizations Medication use AEs Patient satisfaction (TSQM-9) Ig levels | 81 weeks | Period 1: 3 (2 non-adherence, 1 hospitalized due to treatment-related headache) Period 3: 4 (reasons reported under period 4) Period 4: 3 (period 3 and 4 discontinuations included 1 discontinuation due to fatigue, 1 due to non-adherence and 5 discontinuations not related to AEs, 6 of these 7 patients withdrew consent) |
| Viallard 2017 ^23^ | This study was sponsored by LFB Biomédicaments France | Prospective before-after | 7 sites in France | September 2006 and August 2007 | Ig level Infection rate Hospitalizations Antibiotic use School/work days missed Fever episodes AEs | 9 months | 1 did not receive TEGELINE® at regular intervals during the 6 months prior to enrolment |
| Wasserman 2012 ^24^ | Supported by Baxter Healthcare Corporation | Prospective before-after | USA and Canada | NR | Infection rates School/work days missed Hospitalizations Antibiotic use Ig levels Treatment preference AEs | 3 months IVIG, then 14 to 18 months HyQvia  Last follow-up at 17 to 21 months | 6 (due to AEs) |
| Wasserman 2010 ^25^ | Supported by the Talecris Biotherapeutics centre for Science and Education | Prospective before-after | 8 sites in the USA and Canada | November 2006 to August 2008 | Ig levels Infection rates AEs | 29 weeks (5 weeks IVIG, 24 week SCIG) | Run-in phase:  3 (1 due to AE, 1 lost to follow-up, 1 withdrew consent).  SCIG phase:  7 (2 due to AE, 5 due to non-compliance or withdrawn consent). |
| Wasserman 2011 ^26^ | Baxalta now part of Shire | Prospective single arm trial | 9 sites in the USA | (October 3, 2007 to September 1, 2009) | Infection rates Hospitalizations School/work days missed Antibiotic use AEs IG levels | Part 1/Period 1: 13 weeks Part 2/Period 2: 12 weeks Part 3a/Period 3: 6 weeks  Part 3b/Period 4: 12 weeks  Extension period: 5 months  End of study: 1 year and 9 months | 5 (reasons NR) |
| **Single-arm studies (49 studies)** | | | | | | | |
| Ballow 2009 ^27^ | NR | Prospective single-arm trial | 7 sites in USA | NR | Infections AEs Ig levels Antibiotic use | Mean 383 days (range: 181 to 484) | 4 patients withdrew, none due to treatment related AEs |
| Ballow 2016 ^28^ | All phases of this study were supported by Instituto Grifols S.A., the sponsor. | Prospective single-arm trial | 8 sites in USA | NR | AEs Infections Hospitalizations School days missed | 12 months | 1 patient before receiving study drug.  5 patients withdrew early: 4 due to patient/parent or legal guardian decision, 1 due to investigator’s decision |
| Berger 2004 ^29^ | Instituto Grifols, SA, Barcelona, Spain, provided support for this study. The study was conducted under the auspices of Primary immune Services, Inc. (PSI) (Baltimore, MD). | Prospective single-arm trial | 7 sites in the USA | NR | Infection rate School/work days missed Hospitalizations ED visits AEs | 12 months | 3 (5.9%) (2 due to patient/parent or legal guardian decision, 1 reason listed as “other”). |
| Berger 2010 ^30^ | NR | Prospective single-arm trial | 6 sites in USA | NR | Infections School/work days missed Hospitalizations Emergency room visits Antibiotic use AEs | 12 months, follow-up visits scheduled 10 to 14 days, 3 months, and 6 months after the last study infusion | 9 (4 patient or parent decision, 3 due to AEs, 2 reason listed as “other”) |
| Berger 2010 ^31^ | NR | Prospective single arm trial | USA (number of sites NR) | NR | QoL (SF-36, CHQ-PF50) Infection rates Antibiotic treatment Hospitalizations Days missed from work, school or usual activities AEs Ig Levels | 12 months | 6 (2 AEs, 2 withdrawal of consent, 1 lost to follow-up, 1 protocol violation)  Further loss to follow up in the analysis population is not reported |
| Bezrodnik 2013 ^32^ | Grant from CSL Behring | Prospective case series (retrospective IVIG therapy brand not named, ineligible) | 3 sites in Argentina | NR (Study: Prospective period: September 2010 and July 2011) | Ig levels Infection rates AEs | 36 weeks | 2 (1 due to discomfort at injection site, 1 due administration difficulties caused by uncooperativeness) |
| Blazek 2015 ^33^ | Supported by Baxter | Prospective case series (retrospective IVIG therapy brand not named, ineligible) | 20 sites in Austria, Czech Republic, Denmark, France, Germany, Great Britain, Spain and Sweden | NR | Overall efficacy as assessed by investigator Ig level Infection rates Hospitalization | Mean 210.8 days (range: 149 to 287 days) observational period | 9 (1 AE, 1 death due to existing illness, 5 withdrawn by attending physician (4 non drug-related reason, 1 unspecified reason), 1 withdrew consent, 1 lost to follow-up. |
| Borte 2011 ^34^ | This study was supported by CSL Behring AG, Berne, Switzerland | Prospective single arm trial | 6 sites in Canada, Germany, Italy, and Spain | NR | Ig levels HRQoL (SF-36, CHQ-PF50) School/work days missed Infection rate AEs | 6 months | 1 (withdrew consent) |
| Borte 2017 ^35^ | Funded by Octapharma | Prospective single arm trial | 11 sites in the USA and Europe | January 2010 to June 2012 | Infection rates Fever episodes Ig levels Medication use School/work days missed Hospitalizations AEs | 12 months | 2 (1 withdrawn by investigator, 1 protocol violation). |
| Canessa 2017 ^36^ | Funded by CSL Behring, S.p.A., Italy | Prospective single arm trial (in person comparison combines Hizentra and Vivaglobin which is ineligible) | 15 sites in Italy | Data were collected from 1 August 2012 to 9 January 2014. | Ig levels Infection rate School/work days missed AEs Patient satisfaction (TSQM questionnaire) | 6 months | 8 discontinued (due transfer of the patient to other centre or abroad, or unspecified personal reasons) |
| Church 2006 ^37^ | NR | Prospective single arm trial | 11 sites in the USA | NR | Infection rates Hospitalizations Ig levels AEs | Minimum 12 months | 3 (2 withdrew consent, 1 withdrawn by the investigator for non-treatment reasons). |
| Dash 2015 ^38^ | Funded by Bio Products Laboratory limited (BPL) | Prospective single arm trial | 14 sites in the UK | June 2000 to January 2005 | Ig levels Infections School/work days missed Treatment satisfaction | Maximum of 54 months | Stage 1 (6 months): 1 (emigrated) Stage 2 (long term follow-up): 14 (10 withdrew consent, 2 protocol deviations, 1 did not like diary cards, 1 returned to IVIG therapy) |
| Debes 2007 ^39^ | NR | Prospective case series | 310 sites in Germany | (February 1995 to February 2005) | Safety outcomes | Up to 223 infusions  Follow up mean 625 days; median 243 days | NR (safety population analysis) |
| Empson ^40^ | Sponsored by CSL Limited, Australia | Prospective single-arm trial (Previous IVIG brand not reported) | 9 sites in Australia and New Zealand | (April 2007 to October 2009) | Infection rate Ig levels Hospitalization School/work days missed Antibiotic use HRQoL (SF-36, LQI) AEs | 36 weeks (including 12 week wash-in/out period)  Follow up 40 weeks | 7 (4 major protocol violations, 3 patients with insufficiently evaluable blood samples) |
| Euctr 2007 ^41^ | Primary sponsor LFB Biotechnologies (formerly LFB SA) | Prospective single arm trial | 10 sites in France | 13 June 2007 to 17 June 2008 | Infection rate AEs | 31 months | 7 (5 withdrew consent, 2 due to AE) |
| Fasth 2008 ^42^ | Funded by a research grant from Baxter Healthcare | Prospective single-arm trial (Previous IVIG brand not reported) | 1 site in Sweden | (29 November 2004 until 13 October 2006) | Ig levels Infection rates Hospitalization School/work days missed AEs HRQoL (CHQ) | 6 months | None |
| Gardulf 2006 ^43^ | Supported by ZLB Behring, Marburg, Germany | Prospective single arm trial | 12 sites in Austria, Brazil, Germany, Poland, Spain, and Sweden | NR | Ig levels Infection rate School/work days missed AEs | 43 weeks (10 months) | 8 (2 protocol violation, 1 due to a longer stay abroad, 1 suspected severe systemic AE, 2 withdrew consent, 1 moderate local infusion reaction, 1 due to serum IgG trough levels not raising sufficiently) |
| Gupta 2023 ^44^ | Sponsored by Octapharma | Prospective single arm trial | 16 sites in the USA | NR | Ig levels AEs Infection rate Antibiotic use HRQoL Hospitalization Fever episodes School/work days missed | 24 weeks | 9 (4 withdrawal of consent, 3 due to AEs, 1 investigator decision, 1 lost to follow-up) |
| Gustafson 2008 ^45^ | Supported by Baxter AG, Vienna, Austria | Prospective single-arm trial | 1 site in Sweden | NR | Ig levels Infection rate AEs | 24 weeks | None |
| Hagan 2010 ^46^ | Supported by CSL Behring LLC, King of Prussia, PA, USA | Prospective single arm trial | 12 sites in the USA | NR | Infection rates School/work days missed Hospitalizations Antibiotic use Ig levels AEs | 15 months (with 3 month wash-in/wash-out period) | Wash-in/out period:  11 (8 withdrawal of consent, 2 due to AEs, 1 disqualifying laboratory results)  Efficacy period:  11 (6 withdrawal of consent, 1 termination of study site, 1 multiple violations of protocol, 1 lost to follow-up, 1 noncompliance) |
| Hoffmann, 2010 ^47^ | Sponsored by CSL Behring | Prospective case series | 24 sites in Germany | NR | Ig levels Treatment satisfaction HRQoL (SF-36, CHQ-PF50) AEs | 9 months | 22 adults (1 hospitalization for stem cell transplantation, 1 no further Ig treatment necessary, 1 decision of patient, 1 due to AE, 1 end of study reached before patient’s scheduled final visit, 1 progression of underlying malignant disease, 1 patient choice due to discomfort with subcutaneous treatment, 1 psychiatric disease, 14 NR) |
| Jolles 2011 ^48^ | Supported by CSL Behring AG, Berne, Switzerland | Prospective single arm trial | 15 sites in Europe | NR | Ig levels Infection rate School/work days missed Hospitalization Antibiotic use AEs | 40 weeks (including 12 week wash-in/wash-out period) | Wash-in/out period:  5 (3 AEs, 2 withdrew consent);  Efficacy period:  3 (3 AEs) |
| Kanegane 2014 ^49^ | Supported by CSL Behring AG, Berne, Switzerland | Prospective single arm trial (Previous IVIG brand not reported)study) | 9 sites in Japan | NR | Ig levels Infection rates Hospitalization Antibiotic use AEs School/work days missed | 3 months followed by safety follow-up visit 12 to 17 weeks after last SCIG infusion | Wash-in/out period:  1 (1 due to relocation);  Efficacy period:  3 (reason NR) |
| Keith 2022 ^50^ | Funded by Baxalta US Inc. and Baxalta Innovations GmBH, a Takeda company | Prospective single arm trial | 6 sites in Canada | July 1 2018 to August 31 2020 | Ig levels Infection rate AEs HRQoL (TSQM-9, LQI, TPQ) | 12 months | Discontinuations NR separately for PIDS subgroup; overall 20 patients discontinued the study early (reasons NR) |
| Knutsen 2015 ^51^ | Funding for the study was a grant received from CSL Behring | Prospective case series (cohort study, but retrospective IVIG therapy arm mixed brands NR separately) | 1 site in USA | 2014 to 2015 | Ig levels Infection rates S. pneumoniae antibody titres levels | 12 months | None reported |
| Kobayashi 2019 ^52^ | Authors report grants from Octapharma AG during the conduct of the study | Prospective single-arm trial (Previous IVIG brand not reported) | 18 sites in USA, Canada, Hungary, Russia, Slovakia, Poland, Czech republic, | NR | Infection rates Ig levels Hospitalization School/work days missed Antibiotic use Fever episodes AEs HRQoL (CHQ-PF50, SF-36)) | 64 weeks; data collection on a small number of pediatric patients was ongoing at the time of the manuscript. | 6 (6 withdrew due to personal reasons) |
| Krasovec 2007 ^53^ | Supported by Laboratorio de Hemoderivados, Universidad Nacional de Córdoba, Córdoba, Argentina | Prospective single arm trial (Previous IVIG brand not reported) | 1 site in Argentina | NR | Infection rate Medication use School/work days absent Hospitalizations AEs | 7 months | 2 (1 due to worsening of pre-existing Ataxia-Telangiectasia, 1 due to pregnancy) |
| Kreuz 2010 ^54^ | Supported by Biotest AG. | Prospective single arm trial | 5 sites in Germany, Hungary, Italy, Poland and Spain | NR | Infection rate Antibiotic use School/work days missed Hospitalization AEs Ig levels | Mean 47 weeks | None |
| Krivan ^55^ | NR | Prospective single arm trial | 18 sites in France, Hungary, Poland, Serbia and Ukraine | August 2011 to March 2013 | Ig levels Infection rates School/work days missed Hospitalization Fever episode Antibiotic use AEs | Mean 44.5 (SD 11.6) weeks (range: 0.1 to 54.4) | 9 (4 AE, 2 consent withdrawal, 1 late identification of exclusion criterion, 1 pregnancy, 1 omission of last study visit) |
| Krivan 2022 ^56^ | Funded by Biotest AG, Dreieich, Germany | Prospective single arm trial | 17 sites in USA, Hungary, Germany, Spain, Russia | 4 October 2016 to 1 April 2020 | Infection rate Ig level Antibiotic use Time to resolution of infection School/work days missed Hospitalization HRQoL (EQ-5D) AEs | 12 months | 7 discontinued (3 AE, 3 patient decision, 1 withdrew informed consent); 3 removed from per protocol set due to protocol violation |
| Latysheva 2020 ^57^ | Medical writing assistance was funded by Octapharma Pharmazeutika Prod.Ges.m.b.H. | Prospective single arm trial | 5 sites in Russia | NR | Infection rates Antibiotic use Hospitalizations Fever episode School/work days missed HRQoL (SF-36) Ig levels AEs | 8 months | 1 (moved away from study centre) |
| Melamed 2016 ^58^ | This study was funded by Bio Products Laboratory Ltd | Prospective single arm trial | 9 sites in the USA (7 sites), Chile (1 site) and Israel (1 site) | NR | Infection rates School/work days missed Hospitalizations Antibiotic use AEs Ig levels | 12 months  Follow up 15 months | 1 (withdrew consent) |
| Moy 20120 ^59^ | Supported by Bio Products Laboratory | Prospective single arm trial | 7 sites in USA | Date of study February 2006 to November 2007 | Infection rate Antibiotic use Fever episodes Hospitalization Emergency room visit School/work days missed Ig levels AEs | 12 months | 5 (2 AEs, 1 pregnancy, 1 lost to follow-up, 1 withdrew) |
| Nicolay 2005 ^60^ | Supported in part by ZLB Behring, Marburg, Germany | Prospective single-arm trial | Germany, Poland, Sweden, Spain, Brazil, Austria (number of sites NR) | NR | HRQoL (LQI) | 10 months | 7 (reasons NR, reported to be unrelated to patient health) |
| Nicolay 2006 ^61^ | Supported by ZLB Behring LLC, King of Prussia United States | Prospective single-arm trial | USA and Canada (number of sites NR) | NR | HRQoL (SF-36, LQI]) Treatment preference Dropouts | 12 months | 10 (3 withdrawal of consent, 1 lost to follow up, 1 missed the last visit, 3 discontinued due to local infusion site reactions, 1 due to AE, 1 was withdrawn by investigator) |
| Ochs 2004 ^62^ | Supported by Octapharma Pharmazeutika Produktionsges.m.b.H | Prospective single-arm trial | 9 sites in the USA | NR | Infection rates School/work days missed Hospitalization Emergency room visits Ig levels AEs | 12 months | 6 (2 withdrawal of consent, 1 investigator decision and 1 lost to follow-up, 1 death, 1 protocol deviation) |
| Ochs 2006 ^63^ | Sponsored by ZLB Behring, Marburg, Germany | Prospective single-arm trial | International (multicentre, number of sites NR) | NR | Ig levels Infection rates Antibiotic use Hospitalizations School/work days missed AEs | 15 months | 17 (3 withdrew prior to treatment, 6 due to AE, 6 withdrew consent, 1 lost to follow-up, 1 protocol violation) |
| Perez 2021 ^64^ | Funded by GC Pharma, Korea | Prospective single arm trial | 17 sites in USA and Canada | NR | Infection rates Ig levels School/work days missed Hospitalizations Antibiotic use AEs | 12 months  Follow up 13 months | 6 (2 non-compliance, 2 withdrawal of consent, 1 due to melanoma diagnosis, 1 due to inability to obtain IV access) |
| Santaella 2005 ^65^ | NR | Prospective case series | 1 site in Puerto Rico | NR | Ig levels AEs | 3 years | None reported |
| Santamaria 2022 ^66^ | This study was funded in full by Grifols. | Prospective single arm trial | 22 sites in the United Kingdom, Hungary, Germany, Czech Republic, Spain, France, Poland, and Australia | 29 June 2016 through 15 May 2019 | Infection rates Ig levels Antibiotic use Hospitalizations School/work days missed AEs | 52 weeks  Follow up 53 weeks | 1 (withdrawn at sponsor’s request prior to receiving any amount of IGSC 20% due to reported serious AEs) |
| Stein 2009 ^67^ | Financial support was provided by CSL Behring, King of Prussia, PA, USA | Prospective single-arm trial | 19 sites in the USA, France, the UK, Germany, Belgium, and Switzerland | NR | Infection rates School/work days missed Hospitalization Antibiotic use Ig levels AEs | 12 months | 10 (4 withdrew consent, 3 due to AEs, 1 died, 2 protocol violation) |
| Stein 2016 ^68^ | NR | Prospective single-arm trial | 11 sites in the USA (9 sites) and Canada (2 sites) | November 2012 to July 2013 | Infection rates School/work days missed Physician visits Antibiotic use Hospitalizations AEs Ig levels | 12 months  Follow up 14 months | 1 (withdrew consent) |
| Tcheurekdjian 2006 ^69^ | Funded by a grant from Bayer Pharmaceuticals Corporation | Prospective case series | 5 sites in Argentina | October 1998 to September 1999 | AEs | 6 months | None |
| Tuerlinckx 2014 ^70^ | The study received fees for administration support by Central Department for Fractionation of the Red Cross, Brussels, Belgium. | Prospective single-arm trial | Belgium (number of sites NR) | NR | Infection rate Antibiotic use Ig levels | Mean 179 (95% CI 135 to 224) days; median 147 days  Follow up mean 227 days | 1 (reason NR, reported to be unrelated to AE) |
| van der Meer 2011 ^71^ | NR | Prospective single-arm trial | NR | NR | Infections rate Antibiotic use Hospitalizations AEs | 6 months (Part A), 3.5 years (Part B) | 4 (1 NR, 1 due to fever of unknown origin; 1 due to interstitial pneumonia not related to the study drug; 1 due to long-term stay abroad) |
| Vultaggio 2015 ^72^ | Supported by CSL Behring S.p.A. Italy | Prospective case series (retrospective IVIG therapy stage mixed brands NR separately) | 11 sites in Italy | October 2008 to March 2011 | Ig levels Infection rate HRQoL (LQI, treatment satisfaction and 100mm VAS) School/work days missed Hospitalizations AEs | 24 months | 11 (3 lost to follow-up, 4 change of Ig preparations, 1 non-compliance, 3 due to AEs) |
| Vultaggio 2018 ^73^ | Funded by CSL Behring, Italy | Prospective case series (Previous IVIG brand not reported) | 7 sites in Italy | NR | Ig levels Infection rates Hospitalizations Patient satisfaction Antibiotic use AEs | 12 months | 4 (2 withdrew consent, 2 lost to follow-up) |
| Wasserman 2012 ^74^ | Financial support from Biotest Pharmaceuticals Corporation | Prospective single arm trial | 15 sites in the USA | NR | AEs Infection rates Ig levels Antibiotic use Hospitalizations  Infusion rates School/work days missed  Mean dose | 12 months  Follow up 15 months | 12 (5 lost to follow-up when a study site was discontinued, 7 protocol violations) |
| Wasserman 2016 ^75^ | Financial support was provided by ADMA Biologics, Inc., Ramsey, NJ | Prospective single arm trial | 9 sites in the USA | NR | Infection rates School/work days missed Hospitalizations  Infusion  Emergency room visits Antibiotic use AEs Ig levels | 12 months | 2 (due to AE) |

Abbreviations: AE – adverse event; CHQ – Child Health Questionnaire; CHQ-PF50 – Child Health Questionnaire – Parent Form 50; CI – confidence interval; ED – emergency department; EQ-5D - EuroQol- 5 Dimension; HRQoL – health-related quality-of-life; Ig - immunoglobulin; IGIV – immunoglobulin intravenous; IGSC – immunoglobulin subcutaneous; IVIG – intravenous immunoglobulin; IV - intravenous; LQI – Life Quality Index; NR – not reported; PEDS-QL - Pediatric Quality of Life Inventory; PID - primary immunodeficiency; PP – per protocol; RCT – randomized controlled trial; SCIG – subcutaneous immunoglobulin; SD – standard deviation; SF-36 – 36-Item Short Form Health Survey; TPQ - Treatment Perception Questionnaire; TSQM - Treatment Satisfaction Questionnaire for Medication.

#### Table E3: Patient Characteristics

| **Study reference** | **Ig preparation** | **Number of patients** | **PIDDs diagnosis**  **n(%)** | **Pre-existing illness**  **n(%)** | **Previous Ig dose, Mean (SD) mg/kg/infusion** |  |
| --- | --- | --- | --- | --- | --- | --- |
| **Between-person comparative (9 studies)** | | | | | |  |
| Anderson 2021 6  (Prospective cohort study) | Hizentra® 20% (volume cohort) | 15 | CVID: 11 (73.3) Congenital agammaglobulinemia: 1 (6.7) Other immunodeficiency: 3 (20) | Any concomitant disease: 15 (100) Asthma: 9 (60.0) Rhinitis allergic: 7 (46.7) | NR |  |
|  | Hizentra® 20% (flow-rate cohort) | 18 | CVID: 8 (44.4) Congenital agammaglobulinemia: 1 (5.6) Other immunodeficiency: 9 (50) | Any concomitant disease: 18 (100) Asthma: 7 (38.9) Rhinitis allergic: 7 (38.9) | NR |  |
|  | Hizentra® 20% (manual push cohort) | 16 | CVID: 14 (87.5) Other (secondary antibody deficiency and specific antibody deficiency with normal IgG concentration and normal number of B cells): 2 (12.5) | Any concomitant disease: 16 (100.0) Asthma: 8 (50.0) Chronic sinusitis: 7 (43.8) Gastroesophageal reflux disease: 6 (37.5) Depression: 6 (37.5) Bronchiectasis: 4 (25.0) Urinary tract infection: 4 (25.0) Hypothyroidism: 4 (25.0) Hypertension: 4 (25.0) | NR |  |
| Bienvenu 2018 7  (Crossover RCT) | Gammanorm® (rapid push) | 30 | CVID: 17 (56.7*) Hypogammaglobulinemia: 8 (26.7*) Other/NR: 5 (16.7*) | NR | NR |  |
|  | Gammanorm® (pump-infused) |  |  |  |  |  |
| Chapel 2000 8  (Crossover RCT) | Endobulin 5%; | 30 (baseline characteristics NR by arm) | Sweden: CVID: 11 IgG subclass deficiency: 9 Specific antibody deficiency (SAD): 0  Great Britain: CVID: 7 IgG subclass deficiency: 1 SAD: 2 | NR | Treatment naïve - four of the patients were previously untreated (NR which arm) |  |
|  | Gammabulin 16% |  |  |  |  |  |
| Desai 2009 9  (Crossover RCT) | Gamunex 10% IVIG | 12 | NR | NR | NR |  |
|  | Gamunex 10% SCIG |  |  |  |  |  |
| Matamoros 2005 10  (RCT) | Flebogamma 5% | 17 (all patients, baseline characteristics not reported for PIDs subgroup) | CVID: 12 (54%) IgG subclass deficiency: 5 (23) XLA: 3 (14) Hyper IgM syndrome: 1 (4.5) Hyper IgM syndrome and IgG subclass deficiency: 1 (4.5) | NR | NR |  |
|  | Flebogamma 10% | 18 (all patients, baseline characteristics not reported for PIDs subgroup) | NR | NR | NR |  |
| Roifman 2003 11  (RCT) | Gamunex 10% IVIG | 73 | CVID: 46 (53) Hypogammaglobulinemia, unspecified: 31 (36) Congenital hypogammaglobulinemia: 8 (9) Combined immune deficiency: 1 (1) Immunodeficiency with increased IgM: 0 (0) Other immunoglobulin deficiency: 1 (1) | Bronchiectasis: 15 (21) | 434 mg/kg |  |
|  | Gamimune 10% IVIG | 73 | CVID: 44 (52) Hypogammaglobulinemia, unspecified: 24 (28) Congenital hypogammaglobulinemia: 11 (13) Combined immune deficiency: 5 (6) Immunodeficiency with increased IgM: 0 (0) Other immunoglobulin deficiency: 1 (1) | Bronchiectasis: 16 (22) | 452 mg/kg |  |
| Warnatz 2022 12  (Crossover RCT) | Gammanorm® (rapid push) | 30 | Agammaglobulinemia: 1 (3.3) XLA: 1 (3.3) Hypogammaglobulinemia: 4 (13.3) CVID: 23 (76.7) Other: 1 (3.3) | NR | 476 mg/kg/month |  |
|  | Gammanorm® (pump-infused) |  |  |  |  |  |
| Wasserman 2017 13 | Gammaplex® 10%; | 48 | CVID: 38 (79.2) X-linked and autosomal forms of agammaglobulinemia: 8 (16.7) Hypogammaglobulinemia: 2 (4.2) | Abnormal chest X-ray: 5 (10.4) | The selected dose was the same as the dose of IVIG administered during the 3 months prior to study entry |  |
|  | Gammaplex® 5% |  |  |  |  |  |
| Wolf 2003 14  (RCT) | Nanofiltered Sandoglobulin 6% IVIG | 19 | CVID: 14 (73.7*) XLA: 3 (15.8*) IgG subclass deficiency: 2 (10.5*) | Concomitant other diseases that could interfere with the study, fever and acute infection were exclusion criteria | NR |  |
|  | Sandoglobulin 6% IVIG | 17 | CVID: 10 (58.8*) XLA: 5 (29.4*) IgG subclass deficiency: 2 (11.8*) | Concomitant other diseases that could interfere with the study, fever and acute infection were exclusion criteria | NR |  |
| Within-person comparative studies (12 studies) | | | | | |  |
| Bjorkander 2006 15 | | Gammagard 10%; | 22 | CVID: 18 (81.8*) XLA: 2 1 (9.0*) Hypogammaglobulinemia– Good syndrome: 1 (4.5*) Hypogammaglobulinemia with high IgM: 1 (4.5*) | NR | NR |
|  |  | Gammagard S/D 5% |  |  |  |  |
| Bleasel 2012 16 | | Intragam P 6% | 19 | XLA: 2 (10.5) CVID: 17 (89.5) | NR | Range: 0.2 to 0.8 g/kg |
|  |  | Intragam 10 NF 10% |  |  |  |  |
| Borte 2017 17 | | Subcuvia 16% SCIG | 49 | CVID: 32 (65.3) XLA: 9 (18.4) Autosomal recessive hypogammaglobulinemia: 2 (4.1) Hyper-IgM syndrome: 2 (4.1) Specific antibody deficiency with IgG subclass deficiency: 2 (4.1) Specific antibody deficiency: 1 (2.0 ) IgG and IgM deficiency: 1 (2.0) | NR | Range: 0.3 to 1 g/kg/4 weeks for 3 months prior |
|  |  | Gammagard 10% IVIG |  |  |  |  |
|  |  | Cuvitru 20% SCIG |  |  |  |  |
| Heimall 2016 18 | Gamunex 10% SCIG | 12 | NR | NR | Every 3 weeks: 558.3 (93.0)  Every 4 weeks 479.5 (103.9) |  |
|  | Gamunex 10% IVIG |  |  |  |  |  |
| Kallenberg 2007 19 | | Gammagard 6% | 15 | CVID: 12 (85.7) XLA: 1 (7.1) Dysgammaglobulinemia: 1 (7.1) | NR | NR |
|  |  | Gammagard 10% |  |  |  |  |
| Niebur 2015 20 | | Vivaglobin 16%; | 32 | CVID: 13 (40.6) SAD: 11 (34.6) Agammaglobulinemia: 5 (15.7) X-linked hyper IgM (XLHIGM): 2 (6.3) IgG subclass deficiency: 1 (3.1) | NR | NR |
|  |  | Hizentra 20% |  |  |  |  |
| Suez 2016 ^22^ | Gammagard 10% | 77 | XLA: 9 (11.7) Agammaglobulinemia – autosomal recessive: 2/77 (2.6) XLHIGM: 1/77 (1.3) Hyper-IgM - autosomal recessive: 1/77 (1.3) Severe combined immune deficiency: 1/77 (1.3) CVID: 26/77 (33.8) Specific antibody deficiency: 18/77 (23.4) Specific antibody deficiency with IgG subclass deficiency: 7/77 (9.1) Specific antibody deficiency with hypogammaglobulinemia: 10/77 (13.0) Ataxia telangiectasia: 1/77 (1.3) Other: IgG1 and IgG3 subclass deficiency with low IgG: 1/77 (1.3) | NR | Range: 0.3 to 1 g/kg/4 weeks for 3 months prior |  |
|  | Cuvitru 20% |  |  |  |  |  |
| Sleasman 2019 ^21^ | Xembify 20% | 53 | CVID: 41 (77.4) XLA: 5 (9.4) Hyper IgM immunodeficiency syndrome: 2 (3.8) Primary hypogammaglobulinemia: 2 (3.8) SCID (post-transplantation): 2 (3.8) Autosomal recessive agammaglobulinemia: 1 (1.9) | NR | Range: 300 to 800 mg/kg |  |
|  | Gamunex -C 10% |  |  |  |  |  |
| Viallard 2017 23 | | Tegeline® 5% (LFB); | 22 | CVID: 18 (81.8) XLA: 3 (13.6) Hyper-IgM syndrome: 1 (4.5) | NR | NR |
|  |  | ClairYg® 5% (LFB) |  |  |  |  |
| Wasserman 2012 24 | HyQvia 10% IVIG | 87 (baseline characteristics for all 89 enrolled patients are not reported) | CVID: 49 (56.3*) Hypogammaglobulinemia: 17 (19.5*) XLA: 6 (6.9*) IgG subclass deficiency: 4 (4.6*) Specific antibody deficiency: 4 (4.6*) Hyper IgM syndrome: 2 (2.3*) CVID + Hyper IgE syndrome: 1 (1.1*) Dysgammaglobulinemia: 1 (1.1*) Hyper IgE syndrome: 1 (1.1*) SCID: 1 (1.1*) SCID + hypogammaglobulinemia: 1 (1.1*) | NR | Patients were eligible if they had been receiving IgG for ≥3 months before enrolment at a dose of ≥300 mg/kg of body weight/4 weeks |  |
|  | HyQvia 10% SCIG |  |  |  |  |  |
| Wasserman 25 | Gamunex 10% SCIG | 35 | NR | NR | NR |  |
|  | Gamunex 10% IVIG |  |  |  |  |  |
| Wasserman 2011 26 | Gammagard 10% IVIG | 49 | CVID: 27 (55.1*) Hypogammaglobulinemia: 15 (30.6*) XLA: 3 (6.1*) C2 complement deficiency+ IgG deficiency: 1 (2.0*) Combined immune deficiency: 1 (2.0*) Nijmegen chromosome breakage syndrome: 1 (2.0*) Hypogammaglobulinemia secondary to B-cell lymphoma: 1 (2.0*) | NR | Range: 300 to 1,000 mg/kg/month |  |
|  | Gammagard 10% SCIG |  |  |  |  |  |
| Single-arm studies (49 studies) | | | | | |  |
| Ballow 2009 27 | Flebogamma | 50 | NR | NR | All patients had been receiving IVIG replacement therapy at a dose that had not changed by more than 50% of the mean dose before entrance into the study. |  |
| Ballow 2016 28 | Flebogamma | 25 | CVID: 14 (58.3) XLA: 7 (29.2) Other: 3 (12.5) | NR | NR |  |
| Berger 2004 29 | Flebogamma 5% | 51 | CVID: 37 (72.5) X-linked agammaglobulinemia: 12 (23.5) Hyper IgM syndrome: 1 (2.0) Other: 1 (2.0) | NR | NR |  |
| Berger 2010 30 | Flebogamma | 46 | XLA: 8 (17.4) CVID: 37 (80.4) Ataxia Telangiectasia: 1 (2.1) | NR | Range: 300 to 600 mg/kg every 3 or 4 weeks for at least 3 months before enrolment |  |
| Berger 2010 31 | Vivaglobin 16% | 51 | Congenital hypo- or agammaglobulinemia: 11 (21.6) CVID: 33 (64.7) Hyper-IgM: 1 (2.0)  Other: 6 (11.8) | NR | NR |  |
| Bezrodnik 2013 32 | IVIG; Beriglobina P SCIG | 13 | XLA: 3 (20) CVID: 5 (33) Severe hypogammaglobulinemia: 2 (13) SAD: 5 (33) | Bronchiectasis: 5 | Previous IVIG: 556 mg/kg/month |  |
| Blazek 2015 33 | Kiovig/Gammagard liquid 10% | 39 | CVID: 17 XLA: 3 NR: 12 | NR | Ig-naïve: 16 Previously treated: 23 |  |
| Borte 2011 34 | Vivaglobin® 16% | 18 | CVID, ≤1 years: 15 (83) >1 and ≤2 years: 1 (6) >2 and ≤5 years: 1 (6) XLA, >1 and ≤2 years: 1 (6) | NR | Treatment naïve |  |
| Borte 2017 35 | Panzyga® 10% | 51 | CVID: 43/51 (84.3) XLA: 8/51 (15.7) | NR | Range: 200 to 800 mg/kg |  |
| Canessa 2017 36 | Hizentra® 20% | 82 (baseline characteristics data only available for 76 patients) | Known for 76/82 patients: CVID: 53/76 (69.7) XLA: 8/76 (10.5) Autosomal recessive agammaglobulinemia: 2/76 (2.6) Other: 13/76 (17.1) | Infections were reported in the clinical history of:  Visit 1: 7  Visit 3: 22  Visit 6: 24  Autoimmune diseases were reported in the clinical history of:  Visit 1: 4  Visit 3: 3 | NR |  |
| Church 2006 37 | Gammagard 10% | 61 | Either CVID or hypogammaglobulinemia: 41/61 (67.2*) XLA: 5 (8.2*) Ataxia telangiectasia: 1 (1.6*) SCID (adenosine deaminase deficiency): 1 (1.6*) IgG subclass deficiency: 1 (1.6*) Combined IgG and CD 40 deficiency: 1 (1.6*) Hyper-IgE syndrome: 1 (1.6*) PID (no further details): 10 (16.4*) | NR | Range: 300 to 600 mg/kg body weight |  |
| Dash 2015 38 | Subgam 16% |  | CVID: 30 (60*) XLA: 3 (6*) Other: 17 (34*) | Allergy: 18 Cardiovascular: 3 Dermatological: 11 Endocrine: 2 ENT: 17 GI: 10 Hepatobiliary: 1 Lymph nodes: 2 Musculoskeletal: 14 Neurological: 6 Psychiatric: 1 Renal/genitourinary: 2 Respiratory: 16 Other: 14 | 9.87 (range: 2.96 to 18.68) g/L |  |
| Debes 2007 39 | Octagam 5% | 193 | CVID: 74 (38.3*) Agammaglobulinemia: 27 (14.0*) Hypogammaglobulinemia: 15 (7.8*) SCID: 4 (2.1*) Wiskott-Aldrich syndrome: 2 (1.0*) IgG subclass defect: 38 (19.7*) Hyper-IgM syndrome: 6 (3.1*) Ataxia telangiectasia: 3 (1.6*) Other PID’s: 24 (12.4*) | NR | NR |  |
| Empson 40 | Evogam® 16% | 35 | XLA: 2 (5.7) CVID: 33 (94.3) | Bronchiectasis: 11 (31.4) | NR |  |
| Euctr 2007 41 | IGNG/Clairyg (LFB Biotechnologies) | 23 | NR | NR | NR |  |
| Fasth 2008 42 | Subcuvia 16% | 12 | Hypogammaglobulinemia: 4 (33.3) IgG/IgG2 subclass deficiency: 3 (25.0) IgG subclass and IgA deficiency: 1 (8.3) CVID: 1 (8.3) DiGeorge syndrome: 1 (8.3) XLA (Bruton’s agammaglobulinemia): 1 (8.3) X-linked hyper-IgM syndrome (CD40L defect): 1 (8.3) | NR | Median 448.5 (range: 81.0 to 763.0) mg kg−1 month−1 |  |
| Gardulf 2006 43 | Vivaglobin 16% | 60 | Children (2 to 11 years): CVID: 2 Other hypo- or agammaglobulinemia: 11 XLA: 0 IgG subclass deficiency: 2 SCID: 1 Wiskott–Aldrich syndrome: 0 Nijmegen–Breakage syndrome: 0 Adolescents/adults ( ≥12 years):  CVID: 33 Other hypo- or agammaglobulinemia: 7 XLA: 2 IgG subclass deficiency: 0 SCID: 0 Wiskott–Aldrich syndrome: 1 Nijmegen–Breakage syndrome: 1 | 3 of the adolescents/adults had chronic medical complications | A majority of the patients had received a cumulative monthly dose of 400 mg/kg |  |
| Gupta 2023 44 | Cutaquig® 16.5% | 64 | CVID: 57 (89.1) Other: 6 (9.4) XLA: 1 (1.6) | NR | NR |  |
| Gustafson 2008 45 | Subcuvia 16% | 12 | CVID: 11 (91.7*) XLA: 1 (8.3*) | NR | 100 mg/kg body weight/week |  |
| Hagan 2010 46 | Hizentra® 20% | 49, baseline details reported only for analysis pop (n=38) | CVID: 36 (95) XLA: 2 (5) | Patients with ongoing serious bacterial infections at screening, or with other significant medical conditions were excluded from the study. | NR |  |
| Hoffmann, 2010 47 | Vivaglobin® | 82 | CVID: 44 (54) XLA: 13 (16) Severe combined immunodeficiency: 4 (5) Ig subclass deficiency: 4 (5) Other congenital hypo- or agammaglobulinemia: 5 (6) Secondary immunodeficiency: 9 (11) Missing: 3 (4) | NR but 1 patient discontinued for progression of an underlying malignant disease and another because of psychiatric disease | For 20 patients previously on IVIG: 0.39 g/kg; 6 (7%) patients were treatment naïve |  |
| Jolles 2011 48 | Hizentra® 20% | 51; (only 46 patients who began treatment are summarized in patient characteristics) | Patients starting treatment (46): CVID: 28 (60.9) XLA: 17 (37.0) ARAG (autosomal recessive agammaglobulinemia): 1 (2.2) | Patients with ongoing serious bacterial infections at screening, or with other significant medical conditions were excluded from the study. | NR |  |
| Kanegane 2014 49 | Hizentra® 20% | 25 (baseline data only reported for 24 patients who completed wash-in/out period) | XLA: 12 (50) CVID: 10 (41.7) ARAG: 1 (4.2) Hyper IgM syndrome 1 (4.2) | Patients who developed a serious bacterial infection (SBI) at the time of screening or during the mandatory IVIG treatment period were excluded from the study. Other exclusion criteria included lymphoid system malignancy and hyperprolinemia. | Mean dose during IVIG period (weekly equivalent): 77.3 (30.5) mg/kg |  |
| Keith 2022 50 | Cuvitru | 52 | CVID: 43 (70.5) Isolated IG subclass deficiency: 3 (4.9) IgG2 deficiency: 3 (4.9) Specific antibody deficiency with normo- or hypogammaglobulinemia: 2 (3.3) Unclassified antibody deficiency: 5 (8.2) Combined IgA/IgG subclass deficiency: 1 (1.6) Other: 10 (16.4) | NR | NR |  |
| Knutsen 2015 51 | Hizentra® 20% | 13 | XLA: 4 (30.8*)  CVID: 6 (46.2*) SCID post-transplantation: 1 (7.7*) SAD: 2 (15.4*) | NR | NR |  |
| Kobayashi 2019 52 | Cutaquig/Octanorm 16.5% | 61 | CVID: 53 (86.9) XLA: 3 (4.9) Other: 5 (8.2) | NR | Constant IVIG dose between 200 and 800 mg/kg body weight (inclusion criteria) |  |
| Krasovec 2007 53 | Inmunoglobulina G Endovenosa UNC© 5% | 32 (data only reported for 30 patients who completed the study) | XLA: 14 (46.7) CVID: 10 (33.3) Ataxia-Telangiectasia: 4 (13.3) Hyper IgE Syndrome: 1 (3.3) Autosomal Recessive Agammaglobulinemia: 1 (3.3) | Preexisting related chronic conditions: 21 (70): Bronchiectasis: 16 (53.3) Chronic otitis: 2 (6.7) Chronic sinusitis: 2 (6.7) Chronic lobar atelectasis: 1 (3.3) Chronic enteroviral meningoencephalitis: 1 (3.3) Neurological sequelae post bacterial meningitis: 1 (3.3) | NR |  |
| Kreuz 2010 54 | Intratect | 51 | Overall (n=51): CVID: 20 Congenital agammaglobulinemia: 12 Hypogammaglobulinemia: 5 SCID: 1 Ataxia telangiectasia: 5 Hyper-IgM syndrome: 5 Hyper-IgE syndrome: 1 Selective IgG subclass deficiency: 2 | NR | NR |  |
| Krivan 55 | IqYmune® | 62 | Overall (n=62) XLA: 20 (32.3) CVID: 42 (67.7)  Adults (n=36):  XLA: 3 (8.3) CVID: 33 (91.7)  Children (n=26):  XLA: 17 (65.4) CVID: 9 (34.6) Children (n=26): 23 (88.5) | Bronchiectasis: 7 (11.3) patients | NR, 4 (6.5) patients Ig naïve |  |
| Krivan 2022 56 | Yimmugo | 67 | CVID: 53 (79.1) XLA: 10 (14.9) Congenital agammaglobulinemia: 2 (3.0) Congenital hypogammaglobulinemia: 1 (1.5) Other: 1 (1.5) | Drug hypersensitivity: 17 (25.4)  Gastroesophageal reflux disease: 13 (19.4)  Hypertension: 13 (19.4)  Arthralgia:10 (14.9)  Rhinitis allergic: 9 (13.4)  Asthma: 8 (11.9)  Hypothyroidism: 8 (11.9)  Chronic sinusitis:7 (10.4)  Depression: 7 (10.4)  Migraine: 7 (10.4)  Osteoarthritis: 6 (9.0)  Insomnia : 6 (9.0)  Bronchitis chronic: 5 (7.5)  Vitamin D deficiency: 5 (7.5)  Anemia: 5 (7.5)  Iron deficiency anemia: 5 (7.5)  Bronchiectasis: 4 (6.0)  Irritable bowel syndrome: 4 (6.0)  Rhinitis: 4 (6.0)  Hypercholesterolemia: 4 (6.0)  Hyperlipidemia: 4 (6.0)  Anxiety: 4 (6.0)  Headache: 4 (6.0)  Post menopause: 4 (6.0)  Fatigue: 4 (6.0). | NR |  |
| Latysheva 2020 57 | Cutaquig/Octanorm | 25 | CVID: 23 (92.0) XLA: 2 (8.0) | Chronic bronchitis 15 (60.0) Chronic sinusitis 8 (32.0) Chronic tonsillitis: 6 (24.0) Splenomegaly: 5 (20.0) Lymphadenopathy: 5 (20.0) Sinusitis: 5 (20.0) Gastro-oesophageal reflux disease: 4 (16.0) Interstitial lung disease: 3 (12.0) COPD: disease 3 (12.0) Thrombocytopenia: 3 (12.0) Iron-deficiency anemia: 3 (12.0) Gastroduodenitis: 3 (12.0) Polypectomy: 3 (12.0) Chronic urticaria: 3 (12.0) | Range: 0.2 to 0.8 g/kg/month |  |
| Melamed 2016 58 | Gammaplex 5% | 25 | CVID: 22 (88.0) X-linked and autosomal forms of agammaglobulinemia: 3 (12.0) | NR | NR |  |
| Moy 20120 59 | Gammaplex | 50 | CVID: 46 (92.0) XLA: 4 (8.0) | Infection in past 6 months: 27 (54.0) SABI in past 6 months 6: (12.0) Ongoing infections: 2 (4.0 - one patient was completing treatment for bacterial pneumonia at the time of the first Gammaplex® infusion) | NR |  |
| Nicolay 2005 60 | Vivaglobin 16% | 58 | CVID: 35 (60) Hypo- or agammaglobulinemia: 17 (29) Other: 6 (10) | NR | NR |  |
| Nicolay 2006 61 | Vivaglobin 16% | 44 | Group A: CVID: 20 XLA: 8 Group B: CVID: 14 XLA: 2 | NR | NR |  |
| Ochs 2004 62 | Octagam 5% | 46 | CVID: 28 (61) XLA: 13 (28) Hyper IgM syndrome: 2 (4) Functional immunodeficiency: 1 IgG subclass deficiency and hypergammaglobulinemia: 1 Hypogammaglobulinemia: 1 | NR | NR |  |
| Ochs 2006 63 | Vivaglobin 16% | 68 | CVID: 51 (78) XLA: 14 (22) | NR | 116.1 (41.9) mg/kg |  |
| Perez 2021 64 | Alyglo 10% | 49 | Agammaglobulinemia or hypogammaglobulinemia | NR | 538 (SD 120.5) mg/kg/infusion |  |
| Santaella 2005 65 | Gammagard | 20 | CVID: 20 (100) | Presenting symptoms: Infections: 20 Allergies: 13 Bronchial asthma: 10 Malabsorption syndromes: 9 ITP: 5 Aseptic arthritis: 3 Hypothyroidism: 2 Bronchiectasis: 2 Rheumatoid arthritis: 2 Crohn's disease: 1 IgA deficiency: 1 | None - treatment naïve |  |
| Santamaria 2022 66 | Xembify 20% | 61 | CVID: 39 (63.9) XLA: 13 (21.3) Primary hypogammaglobulinemia: 8 (13.1) Hyper IgM immunodeficiency syndrome: 1 (1.6) | NR | 121.45 (36.034) mg/kg |  |
| Stein 2009 67 | Privigen® 10% | 80 | CVID: 59 (73.8) XLA: 21 (26.2) | NR | NR |  |
| Stein 2016 68 | Kedrion | 45 | CVID: 29 (64) Hypogammaglobulinemia: 11 (24) X-linked/Bruton agammaglobulinemia: 5 (11) | NR | 501.7 mg/kg |  |
| Tcheurekdjian 2006 69 | Gamimune N | 65 | CVID: 33 (51) Selective antibody deficiency: 20 (31) IgG subclass deficiency: 14 (22) X-linked agammaglobulinemia: 2 (3) Other: 6 (9) | NR | Range: 200 to 600 mg/kg every 3 to 4 weeks |  |
| Tuerlinckx 2014 70 | Multigam | 23 | CVID: 11 (50) Specific antibody deficiency: 5 (23) XLA: 2 (9) IgG subclasses deficiency: 2 (9) Other agammaglobulinemia: 2 (9) Other: 6 (27) | Severe bacterial infection (episodes): Pneumonia: 7 Proven IPD: 10 | NR |  |
| van der Meer 2011 71 | Nanogam | 18 | CVID: 12 (66.7*) XLA: 6 (33.3*) | NR | NR |  |
| Vultaggio 2015 72 | Vivaglobin 16% | 50 | CVID: 32 (64 ) XLA: 10 (20 ) Others: 8 (16 ) | NR | NR |  |
| Vultaggio 2018 73 | Hizentra® 20% | 36 (baseline data only available for 35 patients) | CVID: 20 (57.1) XLA: 9 (25.7) ARAG: 1 (2.9) Other: 5 (14.3) | Autoimmune disease: 3 (8.6) Other PID-related pathologies: 12 (34.3) | Median: 6 g/infusion every week |  |
| Wasserman 2012 74 | Bivigam 10% | 63 | XLA: 6 (9.5) CVID: 51 (81.0) Other hypogammaglobulinemia: 6 (9.5) | Historical SBI: 8 (12.7) (bacterial pneumonia 7 (11.1); other 1 (1.6)) | Range: 300 to 800 mg/kg |  |
| Wasserman 2016 75 | Asceniv | 59 | CVID: 46 (77.9) XLA: 6 (10.2) Antibody deficiencies: 7 (11.9) | NR | NR |  |

Abbreviations: ARAG - autosomal recessive agammaglobulinemia; C2 – complement component 2; CD-40 - cluster of differentiation 40; COPD – chronic obstructive pulmonary disease; CVID – common variable immunodeficiency; ENT – ear, nose and throat; GI - gastrointestinal; Ig - immunoglobulin; IGNG – immunoglobulin new generation; IPD – inherited platelet disorder; ITP - immune thrombocytopenia; IVIG – intravenous immunoglobulin; NF – nuclear factor; NR – not reported; PID – primary immunodeficiency; PIDD – Primary Immunodeficiency Disorder; RCT – randomized controlled trial; SAD - specific antibody deficiency; SBI - serious bacterial infection; SCIG – subcutaneous immunoglobulin; S/D - signs and symptoms; SD – standard deviation; XLA – X-linked agammaglobulinemia; XLHIGM - X-linked hyper IgM syndrome. * - reviewer-calculated data.

#### Table E4: Overview of Results

| **Outcome** | **Number of studies reporting data** | **Number of studies plotted** | **Number of Ig preparations evaluated** | **IgRT preparations evaluated** | **Range values**  **Details of outliers** |
| --- | --- | --- | --- | --- | --- |
| Annualized rate of overall infection episodes | 43 | 22 | 19 | Alyglo 10% ^64^  Asceniv 10% ^75^  ClairYg 5% ^23^  Cutaquig 16.5% ^44, 57^  Cuvitru 20% ^17, 22^  Gammagard 10% ^17, 22, 26^  Gammanorm 16.5% ^7, 12^  Gammunex-C 10% ^21^  HyQvia 10% ^24^  Hizentra 20% ^46, 48, 73^  Multigam (concentration NR) ^70^  Nanogam 5% ^71^  Panzyga 10% ^35^  Subcuvia 16% ^17^  Subgam 16% ^38^  Tegeline 5% ^23^  Vivaglobin 16% ^31^  Xembify 20% ^21, 66^  Yimmugo 10% ^56^ | 0.76 (95% CI: 0.49 to 1.20) (Gammanorm 16.5% rapid pump ^7^) to 8.92 (95% CI: 6.36 to 12.09) (Subcuvia 16% ^17^)  No outliers identified |
| Annualized rate of SBI episodes | 39 | 27 | 23 | Alyglo 10% ^64^  Asceniv 10% ^75^  Bivigam 10% ^74^  Cuvitru 20% ^17, 22^  Evogam 16% ^40^  Flebogamma 5% ^28^  Gammagard 10% ^17, 22, 26^  Gammaplex 5% ^58^  Gammunex-C 10% ^21^  HyQvia 10% ^24^  Hizentra 20% ^46, 48, 73^  Intratect 5% ^54^  IqYmune 10% ^55^  Kedrion 10% ^68^  Multigam (concentration NR) ^70^  Octagam 5% ^62^  Panzyga 10% ^35^  Privigen 10% ^67^  Subcuvia 16% ^17^  Subgam 16% ^38^  Vivaglobin 16% ^31, 43, 63^  Xembify 20% ^21, 66^  Yimmugo 10% ^56^ | 0 to 0.365 (99% UCI 0.957) (Multigam ^70^)  No outliers identified |
| Annualized rate of hospitalization episodes | 9 | 5 | 6 | Asceniv 10% ^75^  Cuvitru 20% ^17, 22^  Gammagard 10% ^17, 22^  Gammunex-C 10% ^21^  Subcuvia 16% ^17^  Xembify 20% ^21, 66^ | 0.017 (95% CI: 0.008 to 0.033) (IGSC 20% ^66^) to 0.153 (95% CI: 0.062 to 0.316) (Cuvitru 20% ^17^).  No outliers identified |
| Annualized rate of treatment related adverse events | 37 | 35 | 30 | Alyglo 10% ^64^  Asceniv 10% ^75^  Bivigam 10% ^74^  Cutaquig 16.5% ^44, 52, 57^  Cuvitru 20% ^17, 22, 50^  Evogam 16% ^40^  Flebogamma 5% ^28^  Gammagard 10% ^15, 17, 19, 22, 33^  Gammagard 6% ^19^  Gammagard S/D 10% ^15^  Gammanorm 16.5% ^7, 12^  Gammaplex 5% ^58, 59^  Gammunex-C 10% ^21^  Hizentra 20% ^6, 46, 48, 49^  Inmunoglobulina G Endovenosa UNC 5% ^53^  Intragam 10 NF 10% ^16^  Intragram P 6% ^16^  IqYmune 10% ^55^  Kedrion 10% ^68^  Nanofiltered Sandoglobulin 6% ^14^  Nanogam 5% ^71^  Octagam 5% ^39^  Panzyga 10% ^35^  Sandoglobulin 6% ^14^  Subcuvia 16% ^17^  Vivaglobin 16% ^43^  Vivaglobin 16% ^34^  Xembify 20% ^66^  Xembify 20% ^21^  Yimmugo 10% ^56^ | 0.067 (Gammanorm 16.5% ^7^ to 0.84 (Hizentra 20%) ^49^  No outliers identified |
| Trough levels | 56 | 17 | 17 | Beriglobina P. 16% ^32^  Bivigam 10% ^74^  ClairYg 5% ^23^  Flebogamma 10% ^10, 30^  Flebogamma 5% ^10^  Gammagard 10% ^65^  Gammanorm 16.5% ^12^  Gammunex-C 10% ^21^  Gamunex 10% ^9, 25^  Hizentra 20% ^36, 48^  Intragam 10 NF 10% ^16^  Intragram P 6% ^16^  IqYmune 10% ^55^  Multigam (concentration NR) ^70^  Nanogam 5% ^71^  Tegeline 5% ^23^  Xembify 20% ^21, 66^ | 435.22 ^32^ to 1253 mg/dL ^21^  No outliers identified |
| Annualized rate of days of antibiotic use | 15 | 7 | 8 | Cuvitru 20% ^17, 22^  Evogam 16% ^40^  Gammagard 10% ^17, 22, 24^  Gammunex-C 10% ^21^  HyQvia 10% ^24^  IqYmune 10% ^55^  Subcuvia 16% ^17^  Xembify 20% ^21, 66^ | 1.69 (HyQvia 10% SCIG ^24^) to 63.2 (Gammagard 10% ^22^)  1 outlier result, a comparatively lower rate for HyQvia 10% SCIG, 1.69; 95% CI 1.29 to 2.16 ^24^. This may be due to the particularly narrow confidence intervals reported by this study |
| Annualized rate of school/work days missed | 15 | 8 | 8 | Asceniv 10% ^75^  Cuvitru 20% ^17, 22^  Gammagard 10% ^17, 22, 26^  Gammunex-C 10% ^21^  HyQvia 10% ^24^  IqYmune 10% ^55^  Subcuvia 16% ^17^  Xembify 20% ^21, 66^ | 0.23 (HyQvia 10% IVIG ^24^) to 50.42 (Subcuvia 16% SCIG ^17^)  No outliers identified |
| Ann rate infection by trough levels | NA | 7 | 7 | ClairYg 5% ^23^  Gammanorm 16.5% ^12^  Hizentra 20% ^48^  Multigam (concentration NR) ^70^  Nanogam 5% ^71^  Tegeline 5% ^23^  Xembify 20% ^21, 66^ | NA  1 outlier result, a higher rate for XEMBIFY 20% (1,253 mg/dL, 95% CI 1,172.862 to 1,333.138) ^21^. The cause of this higher mean trough level is uncertain, though it may be attributed to the use of dose adjustment from prior IVIG dosing resulting in higher mean Ig doses; with a dose adjustment factor of 1.37 the mean XEMBIFY 20% dose 179 mg/kg, whereas the mean dose for other SCIG studies in this plot was 125.5 mg/kg/week (IGSC 20%, DAF 1.00 ^66^) 492 mg/kg/month and 507 mg/kg/month (Gammanorm 16.5, prior SCIG inclusion criteria thus no dose adjustment ^12^) |
| AEs by trough levels | 9 | 5 | 5 | Gammanorm 16.5% ^12^  Intragam 10 NF 10% ^16^  Intragram P 6% ^16^  Nanogam 5% ^71^  Xembify 20% ^21, 66^ | NA  2 outlier results were identified for XEMBIFY 20% (1,253 mg/dL, 95% CI 1,172.862 to 1,333.138) ^21^ and Nanogam 5% (680 mg/dL, 95% CI 620.325 to 739.675) ^71^. The cause of the higher mean trough levels in the XEMBIFY 20% study may be attributed to dose adjustment resulting in higher doses being administered as discussed above. The authors of the Nanogam 5% study reported that because dosing was based on participants’ prior therapy regimen a lower dose (ranging from 150 to 400 mg/kg every 2 to 5 weeks) was used compared to other studies of similar preparations, which could produce the lower mean trough levels ^71^. It should be noted that the outlying component here was the trough level and not the TRAE which was not outlying and that in the aggregate trough level plotting (Figure E3 in Appendix 3) these preparations were not outlying. |

Abbreviations: CI – confidence interval; DAF – dose adjustment factor; IgRT – immunoglobulin replacement therapy; IGSC – subcutaneous immunoglobulin; IVIG – intravenous immunoglobulin; NA – not applicable; NF - nuclear factor; NR – not reported; SBI – serious bacterial infection; SCIG – subcutaneous immunoglobulin; S/D – signs and symptoms; TRAE – treatment-related adverse event; UCI – uncertainty confidence interval; UNC – Universidad Nacional de Córdoba (Blood Derivatives Laboratory).

#### Table E5: Annualized Outcomes Table

|  | **Mean annualized infection rate** | | | **Mean annualized SBI rate** | | | **Mean annualized rate of hospitalizations** | | |
| --- | --- | --- | --- | --- | --- | --- | --- | --- | --- |
| **Intervention** | **Number of studies**  **(included in plot)** | **Number of patients** | **Mean annualized infection rate reported across studies** | **Number of studies**  **(included in plot)** | **Number of patients** | **Mean annualized infection rate reported across studies** | **Number of studies**  **(included in plot)** | **Number of patients** | **Mean annualized infection rate reported across studies** |
| Alyglo 10% | 1 (1) | 49 | 2.94 | 1 (1) | 49 | 0.022 | 1 (0) | 49 | 0.1 |
| Berigoblina 16% | 1 (0) | 13 | 0.4 | NR | NR | NR | NR | NR | NR |
| Bivigam 10% | 1 (0) | 58 | 2.6 | 1 (1) | 58 | 0.035 | NR | NR | NR |
| ClairYg 5% | 1 (1) | 22 | 4.3 | 1 (0) | 23 | 0.05 | NR | NR | NR |
| Cutaquig 16.5% | 3 (2) | 150 | 2.17 to 3.43 | 1 (0) | 27 | 0.02 | 1 (0) | 61 | 0.04 |
| Cuvitru 20% | 2 (2) | 122 | 2.41 to 4.38 | 2 (2) | 122 | 0.012 to 0.022 | 2 (1) | 122 | 0.02 to 0.153 |
| Endobulin 5% | 1 (0) | 30 | 4.12 | NR | NR | NR | NR | NR | NR |
| Evogam 16% | 1 (0) | 32 | 2.82 | 1 (1) | 32 | 0 | NR | NR | NR |
| Flebogamma 10% | 1 (0) | 46 | 2.2 | 1 (0) | 46 | 0.025 | NR | NR | NR |
| Flebogamma 5% | 2 (0) | 48 | 0.9 to 1.4 | 2 (1) | 70 | 0.021 to 0.051 | NR | NR | NR |
| Gammabulin 16% | 1 (0) | 30 | 3.82 | NR | NR | NR | NR | NR | NR |
| Gammagard 10% | 6 [one IV vs SC] (3) | 270 | 3.4 to 6.3 (3 studies reporting mean annualized rate) | 5 [one IV vs SC] (2) | 248 | 0 to 0.18 | 2 (2) | 110 | 0.05 to 0.119 |
| Gammanorm 16.5% | 2 [2 pump vs rapid push] (2) | 118 | 0.76 to 1.5 | NR | NR | NR | NR | NR | NR |
| Gammaplex 5% | 2 (0) | 75 | 3.2 to 3.28 | 1 (1) | 25 | 0.09 | NR | NR | NR |
| Gamunex 10% | 3 (1) [2 IV vs SC] | 139 | 2 to 4.72 | 2 (1) | 64 | 0.12; <1 | 1 (1) | 53 | 0.06 |
| HyQvia 10% | 1 [IV vs SC] (1) | 168 | 2.97 to 4.51 | 1 [IV vs SC] (1) | 81 | 0.025 | NR | NR | NR |
| Hizentra 20% | 6 (3) | 144 | 0.7 to 5.18 | 6 (3) | 182 | 0 to 0.14 | 2 (0) | 70 | 0.14 to 0.2 |
| Inmunoglobulina G Endovenosa UNC 5% | NR | NR | NR | 1 (0) | 30 | 0.114 | NR | NR | NR |
| Intratect 5% | NR | NR | NR | 1 (1) | 51 | 0.02 | NR | NR | NR |
| IqYmune 10% | 1 (0) | 51 | 3.79 | 1 (1) | 62 | 0.017 | NR | NR | NR |
| Kedrion 10% | NR | NR | NR | 1 (1) | 45 | 0.048 | NR | NR | NR |
| Multigam (concentration NR) | 1 (1) | 22 | 2.77 | 1 (1) | 22 | 0.365 | NR | NR | NR |
| Nanogam 5% | 1 (1) | 17 | 2.01 | NR | NR | NR | NR | NR | NR |
| Octagam 5% | NR | NR | NR | 1 (1) | 46 | 0.115 | NR | NR | NR |
| Panzyga 10% | 1 (1) | 51 | 3.69 | 1 (1) | 51 | 0.08 | NR | NR | NR |
| Privigen 10% | 1 (0) | 80 | 3.55 | 1 (1) | 80 | 0.075 | NR | NR | NR |
| Asceniv 10% | 1 (1) | 59 | 3.44 | 1 (1) | 59 | 0 | 1 (1) | 59 | 0.018 |
| Subcuvia 16% | 1 (1) | 16 | 8.92 | 1 (1) | 16 | 0.27 | 1 (1) | 16 | 0.541 |
| Subgam 16% | 1 (1) | 50 | 3.63 | 1 (1) | 50 | 0.155 | NR | NR | NR |
| Tegeline 5% | 1 (1) | 22 | 4.35 | NR | NR | NR | NR | NR | NR |
| Vivaglobin 16% | 4 (1) | 152 | 0.4 to 4.43 | 5 (3) | 210 | 0 to 0.056 | 1 (0) | 32 | 0 |
| Xembify 20% | 2 (2) | 110 | 2.37 to 2.4 | 2 (2) | 110 | 0.017 to 0.049 | 2 (2) | 110 | 0.017 to 0.049 |
| Yimmugo 10% | 1 (1) | 67 | 2.80 | 1 (1) | 67 | 0.015 | NR | NR | NR |

Abbreviations: IV - intravenous; NR – not reported; SBI – serious bacterial infection; SC - subcutaneous.

#### Table E6: Treatment-Emergent Adverse Events

| **Study** | **Outcome definition and measure** | **Intervention** | **Administration route** | **Timepoint of assessment** | **Proportion of patients who experienced TEAEs**  **n/N (%)** |
| --- | --- | --- | --- | --- | --- |
| Wasserman 2016 ^75^ | Patients experiencing at least 1 drug related treatment emergent AE | Asceniv 10% | IVIG | 12 months | 26/59 (44) |
| Perez 2021 ^64^ | Patients experiencing AEs assessed by the investigator as at least **possibly related** to GC5107 | Alyglo 10% | IVIG | 12 months | 26/49 (53) |
| Wasserman 2012 ^74^ | Patients with at least one drug-related AE | Bivigam 10% | IVIG | 15 months | 40/63 (64) |
| Latysheva 2020 ^57^ | Any related AE **(excluding infections and infusion site reactions)**, coded using the MedDRA | Cutaquig 16.5% | SCIG | 8 months | 3/25 (12) |
| Kobayashi 2019 ^52^ | Any treatment related AE (**excluding infections and infusion site reactions**) | Cutaquig 16.5% | SCIG | 64 weeks | 11/61 (18) |
| Gupta 2023 ^44^ | Patients experiencing systemic treatment-related, temporally associated TEAEs | Cutaquig 16.5% | SCIG | 24 weeks | 30/64 (47) |
| Keith 2022 ^50^ | AEs considered **definitely related** to treatment | Cuvitru 20% | SCIG | 12 months | 5/61 (8) |
| Borte 2017 ^17^ | **Causally related non-serious** AEs, excluding infections | Cuvitru 20% | SCIG | 52 weeks | 20/48 (42) |
| Suez 2016 ^22^ | Patients **experiencing causally related non-serious** AEs | Cuvitru 20% | SCIG | 68 weeks | 28/74 (38) |
| Empson ^40^ | Patients experiencing AEs considered by the investigator to be treatment related | Evogam 16% | SCIG | Week 24 | 14/35 (40) |
| Ballow 2016 ^28^ | Patients experiencing any treatment related AEs | Flebogamma 5% | IVIG | 72 hours | 20/24 (83) |
| Kallenberg 2007 ^19^ | Patients experiencing drug-related AE | Gammagard 10% | IVIG | 2 days after infusion | 3/14 (21) |
| Borte 2017 ^17^ | **Causally related non-serious** AEs, excluding infections | Gammagard 10% | IVIG | 13 weeks | 7/33 (21) |
| Suez 2016 ^22^ | Patients **experiencing causally related non-serious** AEs | Gammagard 10% | IVIG | 13 weeks | 28/77 (36) |
| Bjorkander 2006 ^15^ | Patients experiencing treatment-**related non-serious** AE during treatment | Gammagard 10% | IVIG | Week 9 to week 36 | 7/22 (32) |
| Blazek 2015 ^33^ | Adverse drug reactions related to IVIG 10% administration | Gammagard 10% | IVIG | NR | 12/39 (31) |
| Kallenberg 2007 ^19^ | Patients experiencing drug-related AE | Gammagard 6% | IVIG | 2 days after infusion | 3/14 (21) |
| Bjorkander 2006 ^15^ | Patients experiencing treatment-related **non-serious** AE | Gammagard S/D 10% | IVIG | Week 9 | 6/22 (27) |
| Bienvenu 2018 ^7^ | Patients experiencing treatment related AEs | Gammanorm 16.5% | SCIG, pump and rapid push | 6 months | 2/30 (7) |
| Warnatz 2022 ^12^ | Patients experiencing systemic TEAE considered at least **possibly related** to study treatment | Gammanorm 16.5% | SCIG, pump and rapid push | 6 months | 11/29 (38) |
| Moy 2010 ^59^ | All patients who experienced a treatment related AE | Gammaplex 5% | IVIG | 12 months | 24/50 (48) |
| Melamed 2016 ^58^ | Patients experiencing product related AEs | Gammaplex 5% | IVIG | NR | 14/25 (56) |
| Sleasman 2019 ^21^ | Any **definitely related** AE | Gammunex-C 10% | IVIG | 4 weeks | 8/53 (15.1) |
| Hagan 2010 ^46^ | Patients experiencing AE considered **possibly related** to treatment | Hizentra 20% | SCIG | 12 months | 25/49 (51) |
| Jolles 2011 ^48^ | Patients experiencing AE considered related to Hizentra® administration | Hizentra 20% | SCIG | 40 weeks | 31/51 (60) |
| Kanegane 2014 ^49^ | Patients experiencing any AE considered **plausibly related** to treatment | Hizentra 20% | SCIG | 25 weeks | 21/25 (84) |
| Anderson 2021 ^6^ | Patients experiencing AEs considered treatment-related | Hizentra 20% | SCIG, manual push cohort | Up to 17 weeks per participant | 6/16 (38) |
| Krasovec 2007 ^53^ | Patients who experienced minor AE related to IgG UNC | Inmunoglobulina G Endovenosa UNC 5% | IVIG | 7 months | 6/30 (20) |
| Bleasel 2012 ^16^ | Patients experiencing TEAEs that were considered related to the study treatment | Intragam 10 NF 10% | IVIG | Cycle 7 | 9/19 (47) |
| Bleasel 2012 ^16^ | Patients experiencing AEs possibly relating to intervention | Intragram P 6% | IVIG | Cycle 1 | 2/19 (11) |
| Krivan 2017 ^55^ | Drug-related temporally associated AE (all infection), defined as AEs occurring from the start of infusion up to 72 h after end of infusion | IqYmune 10% | IVIG | 12 months | 33/62 (53) |
| Stein 2016 ^68^ | Patients experiencing treatment related events **(including those considered possibly, probable and certainly related**) | Kedrion 10% | IVIG | 12 months | 14/45 (31) |
| Wolf 2003 ^14^ | Patients experiencing AEs considered to be treatment related | Nanofiltered Sandoglobulin 6% | IVIG | 6 months | 3/19 (15) |
| van der Meer 2011 ^71^ | Patients AE related to study drug | Nanogam 5% | IVIG | 6 months | 10/18 (55.6) |
| Debes 2007 ^39^ | Patients experiencing adverse drug reaction (defined as AE, which are **suspected to be related** to the study medication) | Octagam 5% | IVIG | 10 years | 16/193 (8) |
| Borte 2017 ^35^ | Patients experiencing TEAE (classified by the investigator to be related to the study medication) | Panzyga 10% | IVIG | 12 months | 16/51 (31) |
| Wolf 2003 ^14^ | Patients AEs considered to be treatment related | Sandoglobulin 6% | IVIG | 6 months | 2/17 (12) |
| Borte 2017 ^17^ | **Causally related non-serious** AEs, excluding infections | Subcuvia 16% | SCIG | 12 weeks | 5/16 (31) |
| Gardulf 2006 ^43^ | Patients experiencing systemic AE considered to be related to the study drug | Vivaglobin 16% | SCIG | 43 weeks | 13/52 (25) |
| Borte 2011 ^34^ | Patients experiencing AEs considered "at **least possibly related**" to study medication | Vivaglobin 16% | SCIG | 6 months | 9/18 (50) |
| Santamaria 2022 ^66^ | Adverse reactions (relationship assessed by investigators) | XEMBIFY 20% | SCIG | 53 weeks | 19/61 (31) |
| Sleasman 2019 ^21^ | Any **definitely related** AE | XEMBIFY 20% | SCIG | 24 weeks | 21/49 (42.9) |
| Krivan 2022 ^56^ | Patients experiencing TEAE judged to be treatment-related that occurred more than once | Yimmugo 10% | IVIG | 12 months | 24/67 (36) |

Abbreviations: AE – adverse event; IG - immunoglobulin; IGSC – subcutaneous immunoglobulin; IVIG – intravenous immunoglobulin; NF - nuclear factor; NR – not reported; SCIG – subcutaneous immunoglobulin; S/D - signs and symptoms; TEAE – treatment-related adverse event; UNC – Universidad Nacional de Córdoba (Blood Derivatives Laboratory).

#### Table E7: Risk of Bias Assessment (modified Spitzer et al 1990 tool ^76^)

| **Study ID** | **Study Design** | **1. Does the trial use proper random assignment?** | **2. Did the study use proper sampling?** | **3. Was the sample size adequate?** | **4. Were the criteria for definition or measurement of outcomes objective or verifiable?** | **5. Were outcomes measured with blind assessment?** | **6. Were objective criteria used for the eligibility of subjects?** | **7. Were attrition rates (%) provided?** | **8. Were groups under comparison comparable?** | **9. Are the results generalisable?** |
| --- | --- | --- | --- | --- | --- | --- | --- | --- | --- | --- |
| Anderson 2021 ^6^ | Prospective cohort study | No | No | Uncertain | No | No | Yes | Yes | No | No |
|  |  | Non-randomised | Non-randomised study, doesn't state whether consecutive. | Doesn't state power calculation | Definitions of some outcomes unclear, e.g. unclear if AEs include infections | Study not reported to be blinded | Clear eligibility criteria (both inclusion and exclusion) stated. | Reported number and reasons for patient discontinuation. | Differences in cohorts (e.g. age) reported | Only included patients with specific prior therapy regimen, excluded patients with ongoing SBIs |
| Ballow 2009 ^27^ | Prospective single arm trial | No | No | Uncertain | Yes | No | Uncertain | Yes | N/A | No |
|  |  | Single-arm study | Doesn't state sampling methods | Doesn't state power calculation | Clear definitions used | Study not reported to be blinded | Diagnostic criteria not reported, inclusion criteria specifies types of PIDDs eligible but no clear exclusion criteria | Reported discontinuations | Single-arm study | Only included patients with certain previous IVIG use |
| Ballow 2016 ^28^ | Prospective single-arm trial | No | No | Yes | Yes | No | Yes | Yes | N/A | No |
|  |  | Single-arm study | Doesn't state sampling methods | Study met power calculation | Clear definitions used | Study not reported to be blinded | Diagnostic criteria not clearly reported, though PIDDs diagnoses listed | Reported discontinuations | Single-arm study | Only included patients with certain previous IVIG use |
| Berger 2004 ^29^ | Prospective single-arm trial | No | No | Yes | Yes | No | Yes | Yes | N/A | No |
|  |  | Single-arm study | Doesn't state whether consecutive. | Sample size calculation required 40 patients, 51 were enrolled and analysed. | Clear definitions used, e.g. FDA definition of SBI | Study not reported to be blinded | Clear eligibility criteria (both inclusion and exclusion) stated. | Reported number and reasons for patient discontinuation. | Single-arm study | Only included patients with specific prior therapy regimen, IVIG only which may not reflect modern treatment which includes SCIG |
| Berger 2010 ^30^ | Prospective single-arm trial | No | No | Yes | Yes | No | Yes | Yes | N/A | No |
|  |  | Single-arm study | Doesn't state sampling methods | Study met power calculation | Clear definitions used | Study not reported to be blinded | Eligibility criteria reported. Diagnostic criteria not clearly reported, though PIDDs diagnoses listed. | Reported discontinuations | Single-arm study | Only included patients with certain previous IVIG use |
| Berger 2010 ^31^ | Prospective single arm trial | No | No | Uncertain | Yes | No | Yes | Yes | N/A | No |
|  |  | Single-arm study | Doesn't state whether consecutive. Specific inclusion criteria regarding prior Ig therapy. | Doesn't state power calculation | Clear definitions used. | Study not reported to be blinded (described as open label) | Clear eligibility criteria (both inclusion and exclusion) stated. | Reported number and reasons for patient discontinuation. | Single-arm study | Some forms of PIDS (IgA deficiency) excluded |
| Bezrodnik 2013 ^32^ | Prospective case series | No | No | Uncertain | Yes | No | Yes | Yes | N/A | Yes |
|  |  | Single-arm study | Not reported if patients were included consecutively | Doesn't state power calculation | Primary endpoint SBI rate according to FDA criteria, clear definitions used | Study not reported to be blinded | PAGID and ESID [Conley ME, Notarangelo LD, Etzioni A. Diagnostic criteria for primary immunodeficiencies. Representing PAGID (Pan-American Group for Immunodeficiency) and ESID (European Society for Immunodeficiencies). Clin Immunol. 1999;93(3):190–7.] used for diagnosis of PIDDs | Withdrawals reported | Single-arm study | Patients diagnosed according to PAGID and ESID criteria, first 6 SCIG infusions administered under supervision at primary care setting before home-administrations |
| Bienvenu 2018 ^7^ | Crossover trial | Uncertain | No | Uncertain | No | No | Yes | Yes | Uncertain | No |
|  |  | Method of randomisation not reported | Doesn't state whether consecutive. | Authors report that the sample size was calculated in order to warrant sufficient power for the comparison of LQI factor I, details of calculation not reported | Definitions of some outcomes unclear, e.g. unclear if infections include SBI | Study not reported to be blinded | Clear eligibility criteria (both inclusion and exclusion) stated. | Reported number of withdrawals | Baseline characteristics are not reported by treatment arm | Specific Ig therapy (SCIG) required prior to inclusion |
| Bjorkander 2006 ^15^ | Prospective before-after | No | No | Uncertain | Yes | No | Yes | Yes | N/A | No |
|  |  | Single-arm study | Doesn't state whether consecutive. Specific inclusion criteria regarding prior Ig therapy. | Doesn't state power calculation | Clear definitions used. | Study not reported to be blinded (described as open label) | Clear eligibility criteria (both inclusion and exclusion) and diagnostic criteria stated. | Reported number and reasons for patient discontinuation. | Single-arm study | Some forms of PIDS (IgA deficiency) excluded |
| Blazek 2015 ^33^ | Prospective case series | No | No | Uncertain | Yes | No | Yes | No | Uncertain | No |
|  |  | Single-arm study | Doesn't state whether consecutive. Specific inclusion criteria regarding prior Ig therapy. | Doesn't state power calculation | Clear definitions used for eligible outcomes | Study not reported to be blinded | Clear eligibility criteria (both inclusion and exclusion) stated. | Reported number and reason for premature termination of study participation. Ig data absent for substantial number of patients, reasons not reported | Single-arm study, with analysis according to patients' indication. Characteristics of study subgroups (Ig-naïve and Ig-pretreated) not reported | Patients who previously received IVIG 10% were excluded |
| Bleasel 2012 ^16^ | Prosp Before-after | No | No | Yes | Yes | No | Yes | Yes | N/A | No |
|  |  | Single-arm study | Doesn't state sampling methods | Study met power calculation | Clear definitions used | Study not reported to be blinded (described as open label) | Eligibility criteria reported. Diagnostic criteria not clearly reported, though PIDDs diagnoses listed. | Reported (lack of) discontinuations | Within person comparison | Only included patients with certain previous IVIG use |
| Borte 2011 ^34^ | Prospective single-arm trial | N/A | No | Yes | Yes | No | Yes | Yes | N/A | No |
|  |  | Single-arm study | Doesn't state whether consecutive. | Required sample size of 18, 18 patients recruited and analysed | Clear definitions used, e.g. FDA definition of SBI | Study reported to be open label | Clear eligibility criteria (both inclusion and exclusion) stated. | Reported number of withdrawals | Single-arm study | Only included Ig therapy-naïve patients |
| Borte 2017 ^35^ | Prospective single-arm trial | No | No | Yes | Yes | No | Yes | Yes | N/A | No |
|  |  | Single-arm study | Doesn't state whether consecutive. | Calculated that 50 patients were required, 51 enrolled and analysed | Clear definitions used | Study not reported to be blinded | Clear eligibility criteria (both inclusion and exclusion) stated. | Reported number of withdrawals | Single-arm study | Specific Ig therapy (IVIG) required prior to inclusion |
| Borte 2017 ^17^ | Prosp Before-after | No | No | Yes | Yes | No | Yes | Yes | Uncertain | No |
|  |  | Single-arm study | Doesn't state whether consecutive. Specific inclusion criteria regarding previous treatment and trough IgG levels | Study met power calculation | Clear definitions used | Study not reported to be blinded | Eligibility and diagnostic criteria reported | Reported discontinuations | Single-arm study, although no disaggregated patient characteristics for the two treatment groups in period 1 | Only included patients with certain previous IVIG use. Study conducted in 7 European countries |
| Canessa 2017 ^36^ | Prospective single arm trial | No | No | Uncertain | Yes | No | Yes | No | N/A | No |
|  |  | Single-arm study | Doesn't state whether consecutive. Specific inclusion criteria regarding prior Ig therapy. | Doesn't state power calculation | Clear definitions used, e.g. FDA definition of SBI | Study not reported to be blinded (described as open label) | Clear eligibility criteria (both inclusion and exclusion) and diagnostic criteria stated. | Reported reasons for discontinuations of 8 patients, further patients not analysed for various outcomes/timepoints and reasons are not clear. | Single-arm study | Only included patients with specific prior therapy regimen, Vivaglobin 16% SCIG, or IVIG only which may not reflect modern treatment which includes SCIG |
| Chapel 2000 ^8^ | Crossover trial | Unclear | No | Uncertain | Yes | No | Yes | Yes | Unclear | Yes |
|  |  | Study reported to be randomised but no details of methods used are reported. | Doesn't state whether consecutive. | Authors report choosing 40 participants as an arbitrary, achievable sample size | Clear definitions used (only one outcome, AEs leading to discontinuation, reported at crossover) | Study not reported to be blinded | Clear eligibility criteria (both inclusion and exclusion) stated. | Reported number and reasons for patient discontinuation. | Baseline characteristics are not reported by treatment arm | Inclusion criteria broad, e.g. included treatment naïve patients |
| Church 2006 ^37^ | Prospective single arm trial | No | No | Uncertain | Yes | No | Yes | Yes | N/A | No |
|  |  | Single-arm study | Doesn't state whether consecutive. Specific inclusion criteria regarding prior Ig therapy. | Doesn't state power calculation | Clear definitions used. | Study not reported to be blinded | Clear eligibility criteria (both inclusion and exclusion) and diagnostic criteria stated. | Reported number and reasons for patient discontinuation. | Single-arm study | Only included patients with specific prior therapy regimen, IVIG only which may not reflect modern treatment which includes SCIG |
| Dash 2015 ^38^ | Prospective single arm trial | No | No | Uncertain | Yes | No | Yes | Yes | N/A | Yes |
|  |  | Single-arm study | Doesn't state whether consecutive. Specific inclusion criteria regarding prior Ig therapy. | Reports that overall total was based upon treating the minimum number of children (<12 years) in the study in accordance with draft CPMP SCIG guidelines, no further details reported | Clear definitions used, e.g. infections as rated by clinicians | Study not reported to be blinded | Clear eligibility criteria (both inclusion and exclusion) and diagnostic criteria stated. | Reported number and reasons for patient discontinuation. | Single-arm study | Inclusion criteria broad, e.g. no requirements for certain treatment/Ig levels beyond Ig therapy for at least 6 months |
| Debes 2007 ^39^ | Prospective single-arm trial | No | No | Uncertain | Yes | No | Yes | No | N/A | Yes |
|  |  | Single-arm study | Doesn't state whether consecutive. | Doesn't state power calculation | Reports definition of adverse drug reaction clearly | Study not reported to be blinded | Clear eligibility criteria | No discontinuations or details of treatment duration are reported | Single-arm study | Broad inclusion criteria, included all PIDDs patients receiving Octagam |
| Desai 2009 ^9^ | Crossover trial | Uncertain | No | No | Yes | No | Uncertain | Yes | Uncertain | Uncertain |
|  |  | Method of randomisation not reported | Doesn't state whether consecutive. | Pilot trial, small sample size | Clear definitions used, e.g. FDA definition of SBI | Study not reported to be blinded | Limited reporting on inclusion and exclusion criteria | Reported number of withdrawals | Limited reporting of baseline characteristics | Inclusion criteria are unclear, difficult to assess how generalisable the population is |
| Empson ^40^ | Prospective single-arm trial | No | No | Uncertain | Yes | No | Yes | Yes | N/A | Yes |
|  |  | Single-arm study | Doesn't state whether consecutive. | Doesn't state power calculation | Clear definitions used, e.g. FDA definition of SBI | Study not reported to be blinded | Clear eligibility criteria (both inclusion and exclusion) stated. | Reported number of withdrawals | Single-arm study | Inclusion criteria broad, e.g. no requirements for certain treatment beyond Ig therapy from 0.2 to 0.6 g/kg dose for at least 6 months |
| Euctr 2007 ^41^ | Prospective single arm trial | N/A | No | Uncertain | Yes | No | Uncertain | Yes | N/A | Uncertain |
|  |  | Single-arm study | Doesn't state sampling methods | Doesn't state power calculation, just states target size 25 | Clear definitions used | Study not reported to be blinded | Diagnostic criteria not well reported | Reported discontinuations | Single-arm study | Diagnostic criteria not well reported |
| Fasth 2008 ^42^ | Prospective single-arm trial | No | No | Uncertain | No | No | Yes | Yes | N/A | No |
|  |  | Single-arm study | Doesn't state whether consecutive, likely not due to prior treatment requirements | No formal sample size calculation was done, though tests of significance were conducted | Definitions of infections (i.e. what is "mild" and "moderate") are not reported | Study not reported to be blinded | Clear eligibility criteria (both inclusion and exclusion) stated. | Reports that all patients completed the whole study period | Single-arm study | Specific prior Ig therapy (IVIG) was an inclusion criteria - authors note that as SCIG is standard in Sweden, this led to small sample size |
| Gardulf 2006 ^43^ | Prospective single arm trial | No | No | Uncertain | Yes | No | Yes | Yes | N/A | No |
|  |  | Single-arm study | Doesn't state whether consecutive. | Doesn't state power calculation | Clear definitions used | Study not reported to be blinded | Clear eligibility criteria (both inclusion and exclusion) and diagnostic criteria stated. | Reported number and reasons for patient discontinuation. | Single-arm study | Excluded patients with any history of infusion reaction |
| Gupta 2023 ^44^ | Prospective single-arm trial | No | No | Uncertain | Yes | No | Uncertain | Yes | N/A | No |
|  |  | Single-arm study | Doesn't state whether consecutive. | Doesn't state power calculation | Clear definitions used | Study not reported to be blinded | Detailed inclusion criteria reported, but no exclusion criteria | Reported number of withdrawals | Single-arm study | Specific Ig therapy (SCIG) required prior to inclusion |
| Gustafson 2008 ^45^ | Prospective single-arm trial | No | No | Uncertain | No | No | Uncertain | Yes | N/A | Uncertain |
|  |  | Single-arm study | Doesn't state whether consecutive. | Doesn't state power calculation | Definitions of infections are not reported | Study not reported to be blinded | In the sentence immediately after the inclusion criteria, authors report that "at the time of enrolment, all patients were already on subcutaneous treatment (100 mg per kg body weight and week) and all of them were self-administering at home" - from the wording it is not clear if this was an inclusion criteria or not. | Reports that all patients completed the whole study period | Single-arm study | Inclusion criteria are unclear, difficult to assess how generalisable the population is |
| Hagan 2010 ^46^ | Prospective single arm trial | No | No | Yes | Yes | No | Yes | No | N/A | No |
|  |  | Single-arm study | Doesn't state whether consecutive. | Sample size calculation required enrolment of 32 patients; 49 patients were enrolled and 38 included in primary analyses | Clear definitions used, e.g. FDA definition of SBI | Study not reported to be blinded (described as open label) | Clear eligibility criteria (both inclusion and exclusion) and diagnostic criteria stated. | Ig data absent for substantial number of patients, reasons not reported | Single-arm study | Excluded treatment-naïve patients and with pre-existing infections |
| Heimall 2016 ^18^ | Within-patient before/after study | No | Uncertain | Uncertain | Yes | No | Yes | Yes | NA | Yes |
|  |  | All patients crossed over in the same way from IVIG to SCIG | Doesn't state whether consecutive | Doesn't state power calculation | Clear definitions used | Study not reported to be blinded | Eligibility criteria stated | Reported those enrolled and dropping out | Within person comparison | Diagnostic criteria and PIDDs conditions not reported, though reported to be confirmed PIDDs and prior IVIG therapy details reported |
| Hoffmann, 2010 ^47^ | Prospective case series | No | No | Uncertain | Yes | No | Incomplete | No | Unclear | No |
|  |  | Single-arm study | Doesn't state whether consecutive. Specific inclusion criteria regarding previous treatment and trough IgG levels | Doesn't state power calculation | Clear definitions used for eligible outcomes | Study not reported to be blinded | Eligibility criteria were not well reported: limited inclusion criteria and no exclusion criteria. | Overall discontinuations are reported, but the discontinuations are not reported for various subgroups reported for different outcomes | Single-arm study but comparison of mean serum trough levels in subset of patients who had received IVIG before Vivaglobin treatment and patient characteristics not reported for subset. | Secondary immune disorders are included (<15%) |
| Jolles 2011 ^48^ | Prospective single arm trial | No | No | Uncertain | Yes | No | Yes | Yes | N/A | No |
|  |  | Single-arm study | Doesn't state whether consecutive. | Doesn't state power calculation - uses 30 patients as per EMA 2002 guide notes, no further explanation | Clear definitions used, e.g. FDA definition of SBI | Study not reported to be blinded (described as open label) | Clear eligibility criteria (both inclusion and exclusion) and diagnostic criteria stated. | Reported number and reasons for patient discontinuation. | Single-arm study | Excluded treatment-naïve patients and with pre-existing infections |
| Kallenberg 2007 ^19^ | Retrosp Before-after | No | No | No | Yes | No | Yes | Yes | NA | No |
|  |  | Before-after study | Doesn't state sampling methods | Power calculation stated 15–20 patients would be needed for a coefficient of variation of 50%, but only 14 patients were analysed. | Clear definitions used | Study not reported to be blinded | Diagnostic criteria used is not reported but PIDDs diagnoses are listed | Reported those enrolled and dropping out | Within person comparison | Only included patients with certain previous IVIG use |
| Kanegane 2014 ^49^ | Prospective single arm trial | No | No | Yes | Yes | No | Yes | No | N/A | No |
|  |  | Single-arm study | Doesn't state whether consecutive. Specific inclusion criteria regarding prior Ig therapy. | Sample size calculation required enrolment of 15 patients; 25 patients were enrolled | Clear definitions used, e.g. FDA criteria for SBI | Study not reported to be blinded (described as open label) | Clear eligibility criteria (both inclusion and exclusion) and diagnostic criteria stated. | 1 patient reported to have discontinued, data not available for efficacy outcomes for a further 3 patients and reason for this is not reported | Single-arm study | Only included patients with specific prior therapy regimen, IVIG only, which may not reflect modern treatment which includes SCIG |
| Keith 2022 ^50^ | Prospective single arm trial | No | No | Uncertain | Yes | No | Yes | Yes | N/A | No |
|  |  | Single-arm study | Doesn't state whether consecutive. Only patients who had received SCIG for >=3 months before were eligible. | Doesn't state power calculation | Clear definitions used | Study not reported to be blinded | Eligibility criteria stated | Reported those enrolled and dropping out | Single-arm study | Only included patients with success on SCIG (>=3 months previous use) |
| Knutsen 2015 ^51^ | Prospective case series | No | No | Uncertain | Yes | No | Uncertain | Unclear | N/A | Uncertain |
|  |  | Single-arm study | Doesn't state whether consecutive. | Doesn't state power calculation | Clear definitions used | Study not reported to be blinded | Limited inclusion criteria information is reported | No patient discontinuations are reported, though it is not stated clearly that no patients discontinued. | Single-arm study | Limited reporting of population characteristics |
| Kobayashi 2019 ^52^ | Prospective single arm trial | No | No | Uncertain | Yes | No | Yes | Yes | N/A | No |
|  |  | Single-arm study | Doesn't state whether consecutive. Specific inclusion criteria regarding previous treatment and trough IgG levels | Doesn't state power calculation | Clear definitions used | Study not reported to be blinded (described as open label) | Eligibility and diagnostic criteria reported | Reported discontinuations | Single-arm study | Only included patients with certain previous IVIG use. Authors noted (as a limitation) that there may have been some variation in the patient's condition |
| Krasovec 2007 ^53^ | Prospective single-arm trial | No | No | Uncertain | Yes | No | Uncertain | Yes | N/A | Uncertain |
|  |  | Single-arm study | Doesn't state whether consecutive. | Doesn't state power calculation | Clear definitions used, e.g. FDA definition of SBI | Study not reported to be blinded | With inclusion/exclusion criteria authors report that "all patients had been receiving regular IVIG infusions for ≥ 3 months prior to the study entry" - from the wording it is unclear if this was an inclusion criteria or incidental | Reported number and reasons for patient discontinuation. | Single-arm study | Inclusion criteria are unclear, difficult to assess how generalisable the population is |
| Kreuz 2010 ^54^ | Prospective single arm trial | No | No | Yes | Yes | No | Yes | Yes | NR | No |
|  |  | Single-arm study | Doesn't state whether consecutive patients enrolled . Only patients receiving established replacement therapy using a licensed IVIG were enrolled. | It was planned to enrol at least 50 patients for a 48-week treatment period to achieve adequate statistical power. | Guidance requirements used for acute serious bacterial infection, clear definitions used | Study reported to be open. | Eligibility criteria stated | Reported those enrolled and that no patients discontinued or died | Patient characteristics not reported separately for subgroups according to treatment regimen (3-week and 4-week) | Only included patients receiving established replacement therapy using a licensed IVIG. Authors noted that the patient cohort was heterogeneous. |
| Krivan ^55^ | Prospective single arm trial | No | No | Yes | Yes | No | Yes | Yes | Uncertain | No |
|  |  | Single-arm study | Doesn't state whether consecutive. Specific inclusion criteria regarding PIDD diagnosis and trough IgG levels for previously treated patients. | Sample size calculation required enrolment of 60 patients to ensure power of at least 80%; 62 patients were enrolled. | Clear definitions used. Food and Drug Administration guidance applied for SBIs. | Study reported to be open-label. | Clear eligibility criteria (both inclusion and exclusion) stated. | Reported number and reasons for patient discontinuation. | Subgroups related to adult/children and 3-/4-week dosing regimens. Dosing regimens do not appear to have been compared. | Only included patients with specific PIDD diagnosis and, for previously treated patients, a stable does of Ig and specified trough IgG levels. |
| Krivan 2022 ^56^ | Prospective single arm trial | No | No | Uncertain | Yes | No | Yes | Yes | N/A | No |
|  |  | Single-arm study | Doesn't state whether consecutive. Only patients who had received stable SCIG for >=6 months before were eligible (and >=3 months on specific preparation). | Doesn't state power calculation | Specific diagnostic criteria for SBIs were used as per Food and Drug Administration (FDA) guidance, clear definitions used | Study not reported to be blinded | Eligibility criteria stated | Reported those enrolled and dropping out | Single-arm study | Only included patients with stable IVIG (>=3 months previous use) |
| Latysheva 2020 ^57^ | Prospective single arm trial | No | No | Uncertain | Yes | No | Yes | Yes | N/A | No |
|  |  | Single-arm study | Doesn't state whether consecutive | No power calculation performed | Clear definitions used | Study not reported to be blinded | Eligibility criteria stated | Reported those enrolled and dropping out | Single-arm study | Only included patients with certain previous IVIG use |
| Matamoros 2005 ^10^ | RCT | Unclear | No | Uncertain | Yes | No | Uncertain | No | Yes | Uncertain |
|  |  | Study reported to be randomised but no details of methods used are reported. | Doesn't state whether consecutive. | Reported to be based on the recommendations from the European Agency for the Evaluation of the Medicinal Products and required 35 patients, but details are not reported. | Clear definitions used | Authors report study was not blinded | Limited reporting on inclusion and exclusion criteria | Authors report that 2 patients did not complete the study, reasons for discontinuation not reported | Statistical comparison of baseline characteristics performed, no significant differences | Inclusion criteria are unclear, difficult to assess how generalisable the population is |
| Melamed 2016 ^58^ | Prospective single-arm trial | No | No | Uncertain | Yes | No | Yes | Yes | N/A | No |
|  |  | Single-arm study | Doesn't state whether consecutive. | Doesn't state power calculation | Clear definitions used, e.g. FDA definition of SBI | Study not reported to be blinded | Clear eligibility criteria (both inclusion and exclusion) stated. | Reported number and reasons for patient discontinuation. | Single-arm study | Only included patients with specific prior therapy regimen, IVIG only which may not reflect modern treatment which includes SCIG |
| Moy 20120 ^59^ | Prospective single arm trial | No | No | Yes | Yes | No | Yes | Yes | N/A | No |
|  |  | Single-arm study | Doesn't state sampling methods | Study met power calculation | Clear definitions used | Study not reported to be blinded (described as open-label) | Eligibility criteria reported. Diagnostic criteria not clearly reported, though PIDDs diagnoses listed. | Reported discontinuations | Single-arm study | Only included patients with certain previous IVIG use |
| Nicolay 2005 ^60^ | Prospective single-arm trial | No | No | Uncertain | Yes | No | Uncertain | No | N/A | Yes |
|  |  | Single-arm study | Doesn't state whether consecutive. | Doesn't state power calculation | Clear definitions used, study evaluates LQI tool so scales and measures are reported in detail | Study not reported to be blinded | Limited inclusion criteria information is reported, e.g. exclusion criteria is not reported | Outcome data only reported for patients who were previously on IVIG, comparing treatment satisfaction on LQI to current SCIG; 47 previously received IVIG, but only 39 are reported for this outcome, discontinuations/lost to follow-up unclear. | Single-arm study | Inclusion criteria broad, e.g. no requirements for certain treatment/Ig levels beyond Ig therapy for at least 6 months |
| Nicolay 2006 ^61^ | Prospective single-arm trial | No | No | Uncertain | Yes | No | Uncertain | Yes | N/A | Uncertain |
|  |  | Single-arm study | Self-selected patients rather than a random sample | Doesn't state power calculation | Clear definitions used | Study not reported to be blinded | Criteria is not clearly reported, self-selected patients rather than a random sample | Reported number and reasons for patient discontinuation. | Single-arm study | Inclusion criteria are unclear, difficult to assess how generalisable the population is |
| Niebur 2015 ^20^ | Prosp Before-after | No | No | Uncertain | Yes | No | Yes | Yes | N/A | No |
|  |  | Single-arm study | Doesn't state whether consecutive. | Doesn't state power calculation | Clear definitions used, e.g. SBI as defined by FDA | Study not reported to be blinded | Clear eligibility criteria (both inclusion and exclusion) and diagnostic criteria stated. | Reports no discontinuations | Single-arm study | Inclusion criteria specify particular Ig therapy prior to inclusion |
| Ochs 2004 ^62^ | Prospective single-arm trial | No | No | Yes | Yes | No | Yes | Yes | N/A | No |
|  |  | Single-arm study | Doesn't state whether consecutive. | Sample size calculation required enrolment of 40 patients; 46 patients were enrolled | Outcome definitions clearly described | Study not reported to be blinded | Clear eligibility criteria | Reported number and reasons for patient discontinuation. | Single-arm study | Specific prior Ig therapy (IVIG) as inclusion criteria |
| Ochs 2006 ^63^ | Prospective single-arm trial | No | No | Uncertain | Yes | No | Yes | Yes | N/A | Yes |
|  |  | Single-arm study | Doesn't state whether consecutive. | Doesn't state power calculation | Clear definitions used, e.g. types on infections included in SBI and other infections is reported | Study not reported to be blinded | Clear eligibility criteria (both inclusion and exclusion) stated. | Reported number and reasons for patient discontinuation. | Single-arm study | Inclusion criteria broad, e.g. no requirements for certain treatment/Ig levels beyond Ig therapy for at least 4 months |
| Perez 2021 ^64^ | Prospective single arm trial | No | No | Yes | Yes | No | Yes | Yes | N/A | No |
|  |  | Single-arm study | Doesn't state whether consecutive. | Sample size calculation required enrolment of 40 patients; 49 patients were enrolled | Clear definitions used | Study not reported to be blinded | Clear eligibility criteria (both inclusion and exclusion) and diagnostic criteria stated. | Reported number and reasons for patient discontinuation. | Single-arm study | Only included patients with specific prior therapy regimen, excluded patients with ongoing SBIs |
| Roifman 2003 ^11^ | RCT | Yes | Uncertain | Yes | Yes | Uncertain | Yes | Yes | Yes | Yes |
|  |  | Randomization was achieved by a list of unique block random codes and supplied to each pharmacist | Reported that investigators screened charts of patients potentially eligible to participate and enrolled eligible patients, not reported whether potential participants were consecutively selected or sampled randomly. | Sample size calculated to require 76 patients in each arm, this sample size was achieved | Primary endpoint SBI rate according to FDA criteria, clear definitions used | Reported that the investigator, patient, infusionist and trial nurse were blinded to the preparation administered during any given drug infusion, though methods not reported. | Patients with PIDDs diagnosed according to WHO criteria | Withdrawals reported | Authors note uneven distribution of patients with Combined Immune Deficiency (CID) between the treatment groups, no statistical comparison reported though descriptive statistics suggest groups were similar. | Population diagnosed with PIDDs according to WHO criteria in hospital treatment setting |
| Santaella 2005 ^65^ | Prospective case series | No | No | Uncertain | Yes | No | Yes | Yes | N/A | Uncertain |
|  |  | Single-arm study | Doesn't state sampling methods | Doesn't state power calculation | Clear definitions used | Study not reported to be blinded | Eligibility criteria stated | Reported those enrolled and analysed | Single-arm study | Diagnostic criteria and patient characteristics not well reported |
| Santamaria 2022 ^66^ | Prospective single-arm trial | No | No | Yes | Yes | No | Yes | Yes | N/A | No |
|  |  | Single-arm study | Doesn't state whether consecutive. | Required sample was 40, 61 subjects analysed | Clear definitions used, e.g. FDA definition of SBI | Study not reported to be blinded | Clear eligibility criteria (both inclusion and exclusion) stated. | Reported number and reasons for patient discontinuation. | Single-arm study | Only included patients with specific prior therapy regimen |
| Sleasman 2019 ^21^ | Prospective before-after | No | No | Yes | Yes | No | Yes | Yes | N/A | No |
|  |  | Single-arm study | Doesn't state whether consecutive. Specific inclusion criteria regarding prior Ig therapy. | Sample size calculation required enrolment of 42 patients; 53 patients were enrolled. | Clear definitions used. | Study not reported to be blinded (described as open label) | Clear eligibility criteria (both inclusion and exclusion) stated. | Reported number and reasons for patient discontinuation. | Single-arm study | Some forms of PIDS (IgA deficiency) excluded |
| Stein 2016 ^68^ | Prospective single arm trial | No | No | Uncertain | Yes | No | Yes | Yes | N/A | No |
|  |  | Single-arm study | Doesn't state whether consecutive. Specific inclusion criteria regarding prior Ig therapy. | Doesn't state formal power calculation but does report number of evaluable patients required to be treated for 12 months to determine rate of SBI. Paediatric enrolment was reported to be lower than required by the FDA and European Medicines Agency for market approval | Clear definitions used. Food and Drug Administration guidance applied for SBIs. | Study not reported to be blinded | Clear eligibility criteria (both inclusion and exclusion) stated. | Reported number and reasons for patient discontinuation. | Single-arm study | Only included patients with specific prior therapy regimen |
| Stein 2009 ^67^ | Prospective single-arm trial | No | No | Uncertain | Yes | No | Yes | Yes | N/A | No |
|  |  | Single-arm study | Doesn't state whether consecutive. | Doesn't state power calculation | Outcome definitions clearly described, e.g. FDA definition of SBI | Study not reported to be blinded | Clear eligibility criteria | Reported number and reasons for patient discontinuation. | Single-arm study | Specific prior Ig therapy (IVIG) as inclusion criteria |
| Suez 2016 ^22^ | Prosp Before-after | No | No | Yes | Yes | No | Yes | Yes | N/A | No |
|  |  | Single-arm study | Doesn't state whether consecutive. Specific inclusion criteria regarding previous treatment and trough IgG levels | Study met power calculation | Clear definitions used | Study not reported to be blinded (described as open label) | Eligibility and diagnostic criteria reported | Reported discontinuations | Single-arm study | Only included patients with certain previous IVIG use |
| Tcheurekdjian 2006 ^69^ | Prospective case series | No | No | Uncertain | Yes | No | Yes | Yes, authors reported no withdrawals | N/A | Yes |
|  |  | Single-arm study | Not reported if patients were included consecutively | Doesn't state power calculation | Definition of adverse events reported | Study not reported to be blinded | Patients with PIDDs diagnosed according to WHO criteria | | Single-arm study | Population diagnosed with PIDDs according to WHO criteria in hospital treatment setting |
| Tuerlinckx 2014 ^70^ | Prospective single-arm trial | No | No | Uncertain | Yes | No | Uncertain | Yes | N/A | Uncertain |
|  |  | Single-arm study | Doesn't state whether consecutive. | Doesn't state power calculation | Clear definitions used, e.g. FDA definition of SBI | Study not reported to be blinded | Limited inclusion criteria information is reported | Reported number and reasons for patient discontinuation. | Single-arm study | Previous IVIG therapy details not reported |
| van der Meer 2011 ^71^ | Prospective single-arm trial | No | No | Uncertain | No | No | Yes | Yes | N/A | No |
|  |  | Single-arm study | Doesn't state whether consecutive. | Doesn't state power calculation | Definitions of some outcomes unclear, e.g. severe infections | Study not reported to be blinded | Clear eligibility criteria (both inclusion and exclusion) stated. | Reported number of withdrawals | Single-arm study | Patient characteristics poorly reported |
| Viallard 2017 ^23^ | Prospective single-arm trial | N/A | No | Yes | Yes | No | Yes | Yes | N/A | No |
|  |  | Single-arm study | Doesn't state whether consecutive. | Required sample size was 20, 21 analysed | Clear definitions used, e.g. FDA definition of SBI | Study not reported to be blinded | Clear eligibility criteria (both inclusion and exclusion) stated. | Reported number of withdrawals | Single-arm study | Only included patients with specific prior therapy regimen |
| Vultaggio 2015 ^72^ | Prospective case series (cohort study, retrospective IVIG therapy stage mixed brands NR separately and thus ineligible, extracted as single-arm study) | No | No | Uncertain | Yes | No | Uncertain | Yes | N/A | Uncertain |
|  |  | Single-arm study | Inclusion of patients was decided according to the possibility and willingness to begin using the SCIG therapy, as assessed by the local trialist. | Doesn't state power calculation - uses 30 patients as similar to sample sizes in prior trials | Clear definitions used, e.g. SBI as defined by FDA | Study not reported to be blinded | Inclusion of patients was decided according to the possibility and willingness to begin using the SCIG therapy, as assessed by the local trialist - few details on how this assessment was made | Reported number and reasons for patient discontinuation. | Single-arm study | Inclusion criteria are unclear, difficult to assess how generalisable the population is |
| Vultaggio 2018 ^73^ | Prospective case series | No | No | Uncertain | Yes | No | Yes | Yes | N/A | No |
|  |  | Single-arm study | Doesn't state whether consecutive. | Authors state that sample size was determined based on feasibility criteria, no further information | Clear definitions used, e.g. FDA definition of SBI | Study not reported to be blinded | Clear eligibility criteria (both inclusion and exclusion) stated. | Reported number and reasons for patient discontinuation. | Single-arm study | Only included patients with specific prior therapy regimen (Hizentra weekly) |
| Warnatz 2022 ^12^ | Crossover trial | Uncertain | No | Yes | Yes | No | Yes | Yes | Uncertain | No |
|  |  | Method of randomisation not reported | Doesn't state whether consecutive. | Required sample was 27, 29 analysed | Clear definitions used | Study not reported to be blinded | Clear eligibility criteria (both inclusion and exclusion) stated. | Reported number of withdrawals | Baseline characteristics are not reported by treatment arm | Specific Ig therapy (SCIG) required prior to inclusion |
| Wasserman ^25^ | Prosp Before-after | No | No | Uncertain | Yes | No | Yes | Yes | N/A | No |
|  |  | Single-arm study | Doesn't state whether consecutive. | Doesn't state power calculation | Clear definitions used | Study not reported to be blinded | Clear eligibility criteria (both inclusion and exclusion) stated. | Reported number of withdrawals | Single-arm study | Screening required for all participants to standardise them to previous Ig therapy (IVIG) |
| Wasserman 2011 ^26^ | Prosp Before-after | No | No | Uncertain | Yes | No | Yes | Yes | N/A | No |
|  |  | Single-arm study | Doesn't state whether consecutive. | Doesn't state power calculation | Clear definitions used, e.g. FDA definition of SBI | Study not reported to be blinded | Clear eligibility criteria (both inclusion and exclusion) stated. | Reported number of withdrawals | Single-arm study | Only included patients with specific prior therapy regimen |
| Wasserman 2012 ^24^ | Prosp Before-after | No | No | Yes | Yes | No | Uncertain | No | N/A | No |
|  |  | Single-arm study | Participants not included consecutively, around 45 carried over from previous trial | The power to reject the null hypothesis of ≥1 VASBIs per year at the 1% level of statistical significance against a 1-sided alternative was calculated to require minimum 76 patients; 89 patients were enrolled | Clear definitions used | Study not reported to be blinded | Limited reporting, any exclusion criteria are not reported | Reasons for discontinuation of 2 subjects among 89 enrolled prior to receiving study drug are not reported | Single-arm study | Substantial proportion of patients carried over from a previous trial (45 patients) |
| Wasserman 2012 ^74^ | Prospective single arm trial | No | No | Uncertain | Yes | No | Yes | Yes | N/A | Yes |
|  |  | Single-arm study | Not reported if patients were included consecutively | Doesn't state power calculation | Primary endpoint SBI rate according to FDA criteria, clear definitions used | Study not reported to be blinded | Eligibility criteria stated | Withdrawals reported | Single-arm study | Diagnostic criteria not reported but PIDDs conditions and prior IVIG therapy details reported |
| Wasserman 2029 ^75^ | Prospective single-arm trial | N/A | No | Uncertain | Yes | No | Yes | Yes | N/A | No |
|  |  | Single-arm study | Doesn't state whether consecutive. | Doesn't state power calculation | Clear definitions used | Study not reported to be blinded | Clear eligibility criteria (both inclusion and exclusion) stated. | Reported number of withdrawals | Single-arm study | Only included patients with specific prior therapy regimen (IVIG) |
| Wasserman 2017 ^13^ | Crossover trial | Unclear | No | Uncertain | Yes | No | Yes | Yes | NA | No |
|  |  | Method of randomisation not reported | Doesn't state whether consecutive. Specific inclusion criteria regarding previous treatment and trough IgG levels | Doesn't state power calculation | Clear definitions used | Study not reported to be blinded | Eligibility criteria stated | Reported those enrolled and dropping out | Within person comparison | Only included patients with certain previous IVIG use and baseline IgG levels |
| Wolf 2003 ^14^ | RCT | Unclear | No | Uncertain | Yes | Unclear | Unclear | No | No | No |
|  |  | Method of randomisation not reported | Doesn't state whether consecutive. Specific inclusion criteria regarding PIDD diagnosis and trough IgG levels for previously treated patients. | Doesn't state power calculation | Clear definitions used, e.g. FDA definitions used for SBIs. | Study reported to be blinded but methods NR | Exclusion criteria reported but inclusion criteria information is limited | Reasons for discontinuations is not reported | Patients randomized to Sandoglobulin were, on average, older than those on IVIG-N. Moreover, in the PIDD study, the IVIG-N group included a greater number of patients with recurrent chronic respiratory tract infections (n = 10) than the Sandoglobulin group (n = 5). | Only included patients with specific PIDD diagnosis and platelet count |

Abbreviations: CPMP - Committee for Proprietary Medicinal Products; EMA - European Medicines Agency; ESID - European Society for Immunodeficiencies; FDA - Federal Drug Administration; ID – identifier; IVIG - intravenous immunoglobulin; LQI - Life Quality Index; NA - not applicable; NR - not reported; PAGID - Pan-American Group for Immunodeficiencies; PIDD - primary immunodeficiency diseases; SBI - serious bacterial infection; RCT - randomised controlled trial; SCIG - subcutaneous immunoglobulin; WHO - World Health Organisation.

## Data Plots (hospitalizations, trough levels, infection and adverse event rate by trough levels, antibiotic use, school/work days missed and quality of life summary)

**Hospitalizations**

Eight studies reported the annualized rate of hospitalization with sufficient data for inclusion in inclusion in a visualization plot, assessing 6 immunoglobulin preparations. Two preparations were investigated in more than one study.

#### Figure E2: Rate of Hospitalisation Per Patient Year


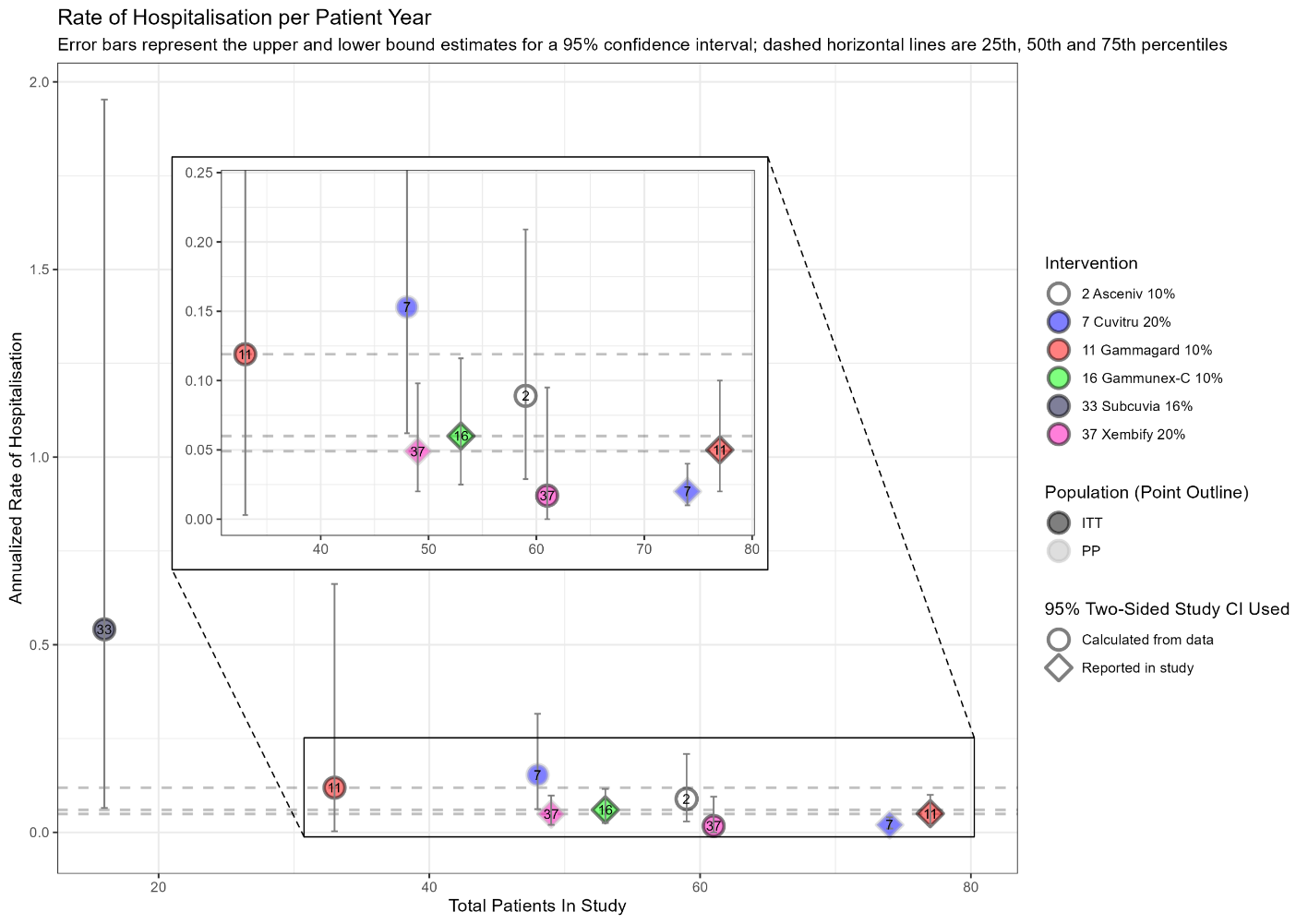


**Trough levels**

Seventeen studies reported mean trough levels with sufficient data for allow for our additional analysis (including a measure of variance) and inclusion in a visualization plot. Where reported, the mean trough level at the final infusion was used. If the mean level at final infusion was not reported, the mean trough level across the study duration was used. Study durations, infusion schedules and thus almost by definition the timepoint of final infusion varied across studies

Seventeen preparations were assessed across the 17 plotted studies, each of which was investigated in 1 study except Flebogamma 10% which was evaluated by 2 studies ^10, 30^. Trough levels ranged from 435.22 ^32^ to 1,253 mg/dL ^21^. Based on the naïve comparison of confidence intervals, no outliers were identified among the data points.

#### Figure E3: Mean Ig Trough Level


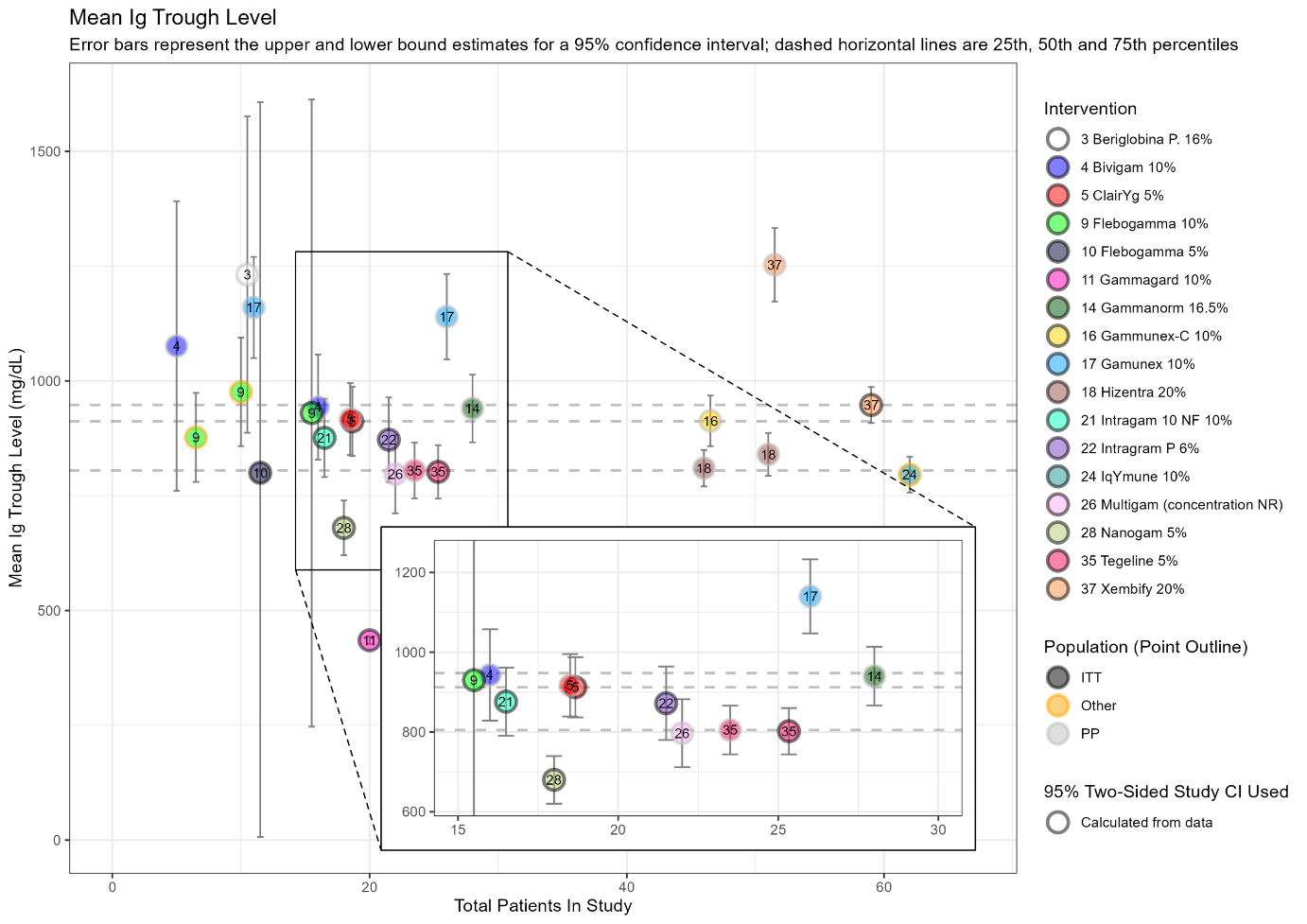


**Antibiotic use**

Antibiotic use was not reported in all studies in a manner that enabled aggregation for cross-comparison, in that some differentiated therapeutic and prophylactic use while others did not specify. Eight studies evaluating 8 Ig preparations reported the annualized rate of days of either therapeutic or overall antibiotic use (rates of specifically prophylactic antibiotic use are not included) 3 studies permitted the use of prophylactic antibiotics ^21^ ^40, 66^; the other 4 either didn’t permit their use or didn’t clearly report this. 3 preparations were assessed by more than one study: Gammagard 10% IVIG (n=3) ^17, 22^, Cuvitru 20% SCIG (n=2) ^17, 22^ and Xembify 20% (n=2) ^21,^ ^66^. The annualized rate of days of antibiotic use ranged from 1.69 (HyQvia 10% SCIG ^24^) to 63.2 (Gammagard 10% ^22^). Based on the naïve comparison of confidence intervals 1 outlier result was identified. 1 outlier result (a comparatively lower rate for HyQvia 10% SCIG, 1.69; 95% CI 1.29 to 2.16 ^24^) may be due to the particularly narrow confidence intervals reported by this study.

#### Figure E4: Rate of Days of Antibiotic Use per Patient Year


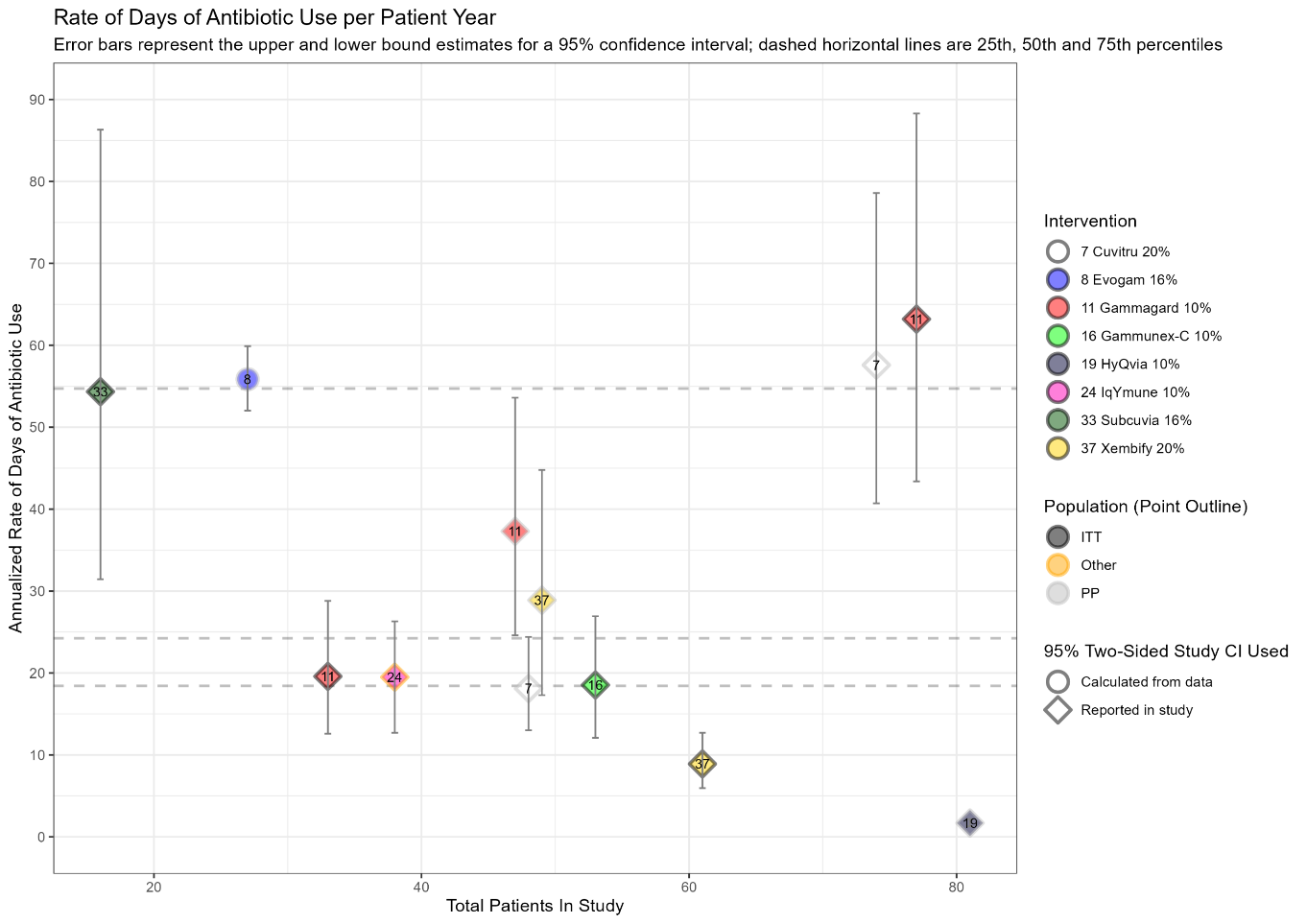


**School/work days missed**

School/work days missed was reported at different timepoints and using individual study-specific definitions. 8 studies evaluating 8 preparations reported the annualized rate of school/work days missed with 3 preparations assessed by more than one study: Gammagard 10% IVIG (n=3) ^17, 22^,Cuvitru 20% SCIG (n=2) ^17, 22^ and Xembify 20% (n=2) ^21,66^. 4 studies permitted the use of prophylactic antibiotics (the number of patients concerned was not reported) ^21, 26, 66, 74^; the other 4 either didn’t permit their use or didn’t clearly report this. The annualized rate ranged from 0.23 (HyQvia 10% IVIG ^24^) to 50.42 (Subcuvia 16% SCIG ^17^). It should be noted that these studies, like for the other categories, originated from a variety of countries that have in some cases distinct social norms around work and school attendance and policies. Based on the naïve comparison of confidence intervals, no outliers were identified among the data points.

#### Figure E5: Rate of School and Work Days Missed per Patient Year


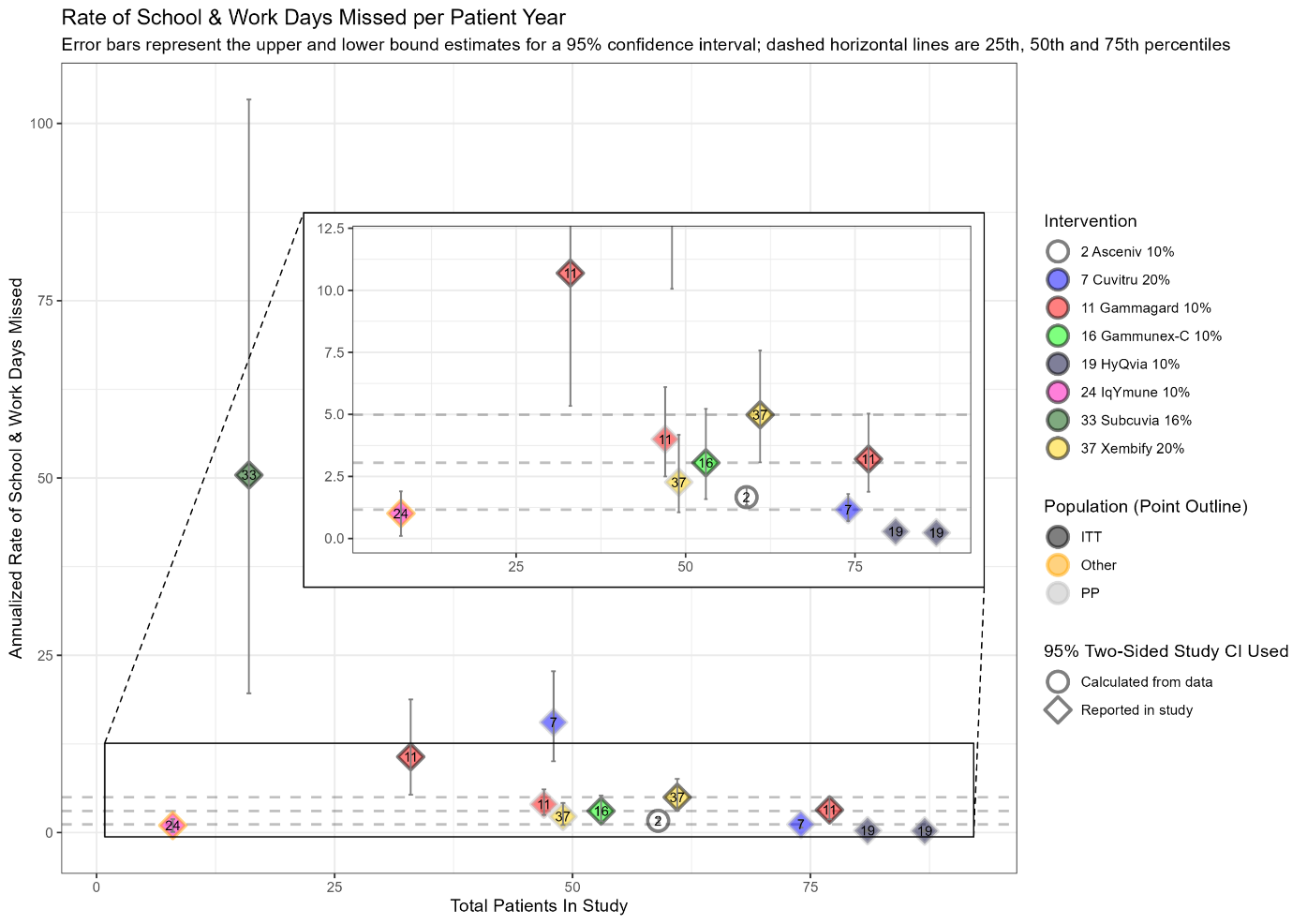


**Outcomes by trough levels**

An effort was made to plot individual studies to denote their mean Ig levels and outcome data simultaneously for exploratory purposes and in an effort to understand potential relationships between Ig level and outcomes. Sufficient data was available to produce a visualization plot for annualized infection rate and proportion of patients experiencing adverse events according to their mean Ig trough level. Only studies for which the outcomes were reported with a measure of precision (either reported with confidence intervals or with sufficient data for confidence intervals to be calculated) were included in the comparison visualization plots. The plots should be interpreted cautiously in the context of the limited comparative data that was available and potentially high heterogeneity and do not appear to show any strong relationships between the plotted outcomes. Given published references performing meta-regression analyses that demonstrated significance between dose, trough and infection ^2, 4^, we were surprised by this finding. Our best explanation for this would be the consistent high trough levels in the more recent prospective studies used to create this scoping review. This might have been a feature of time window utilized as older studies tended to have lower trough levels and higher rates of infections ^4^. Additionally, this was intentionally a scoping review and not a meta-analysis as denoted by method and title, and thus we were not empowered to perform a meta-regression analysis as has been performed by other authors. This lack of obvious visual relationships should not be taken as any statement regarding the previous meta-regression analyses and their conclusions.

Infection

6 studies (evaluating 7 different Ig preparations) reported sufficient data for inclusion in a data plot showing annualized infection rate by Ig trough level.1 potential outlier Ig level result was identified for XEMBIFY 20% (1,253mg/dL, 95% CI 1,172.862 to 1,333.138) ^21^. The cause of the higher mean trough levels in the XEMBIFY 20% study is uncertain, though it may be attributed to the use of dose adjustment from prior IVIG dosing resulting in higher mean Ig doses; with a dose adjustment factor (DAF) of 1.37 the mean XEMBIFY 20% dose was 179 mg/kg, whereas the mean dose for other SCIG studies in this plot was 125.5 mg/kg/week (IGSC 20%, DAF 1.00 ^66^) 492 mg/kg/month and 507mg/kg/month (Gammanorm 16.5, prior SCIG inclusion criteria thus no dose adjustment ^12^).

#### Figure E6: Mean Trough Level by Annualized Overall Infection Rate


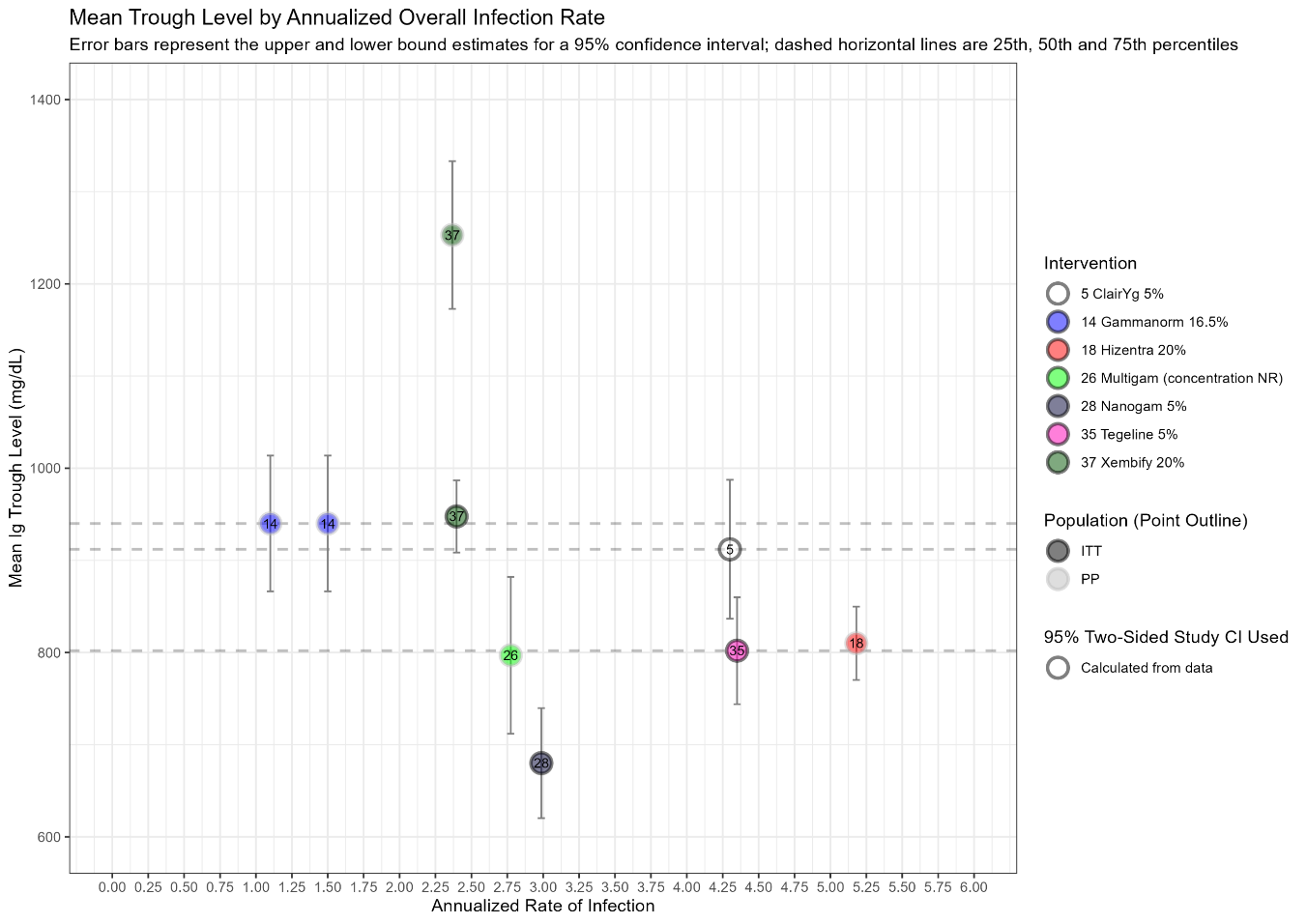


Adverse events

5 studies (evaluating 5 different Ig preparations) reported sufficient data for inclusion in a data visualization plot showing the proportion of patients experiencing adverse events by Ig trough level.

Based on the naïve comparison of confidence intervals potential outlier Ig level results were identified for XEMBIFY 20% (1,253 mg/dL, 95% CI 1,172.862 to 1,333.138) ^21^ and Nanogam 5% (680 mg/dL, 95% CI 620.325 to 739.675) ^71^. The cause of the higher mean trough levels in the XEMBIFY 20% study may be attributed to dose adjustment resulting in higher doses being administered as discussed above. The authors of the Nanogam 5% study reported that because dosing was based on participants’ prior therapy regimen a lower dose (ranging from 150 to 400 mg/kg every 2 to 5 weeks) was used compared to other studies of similar preparations, which could produce the lower mean trough levels ^71^. It should be noted that the outlying component here was the trough level and not the TRAE which was not outlying and that in the aggregate trough level plotting (Figure E3 above) these preparations were not outlying.

#### Figure E7: Mean Ig Trough Level by Proportion of Patients with Treatment Related AEs


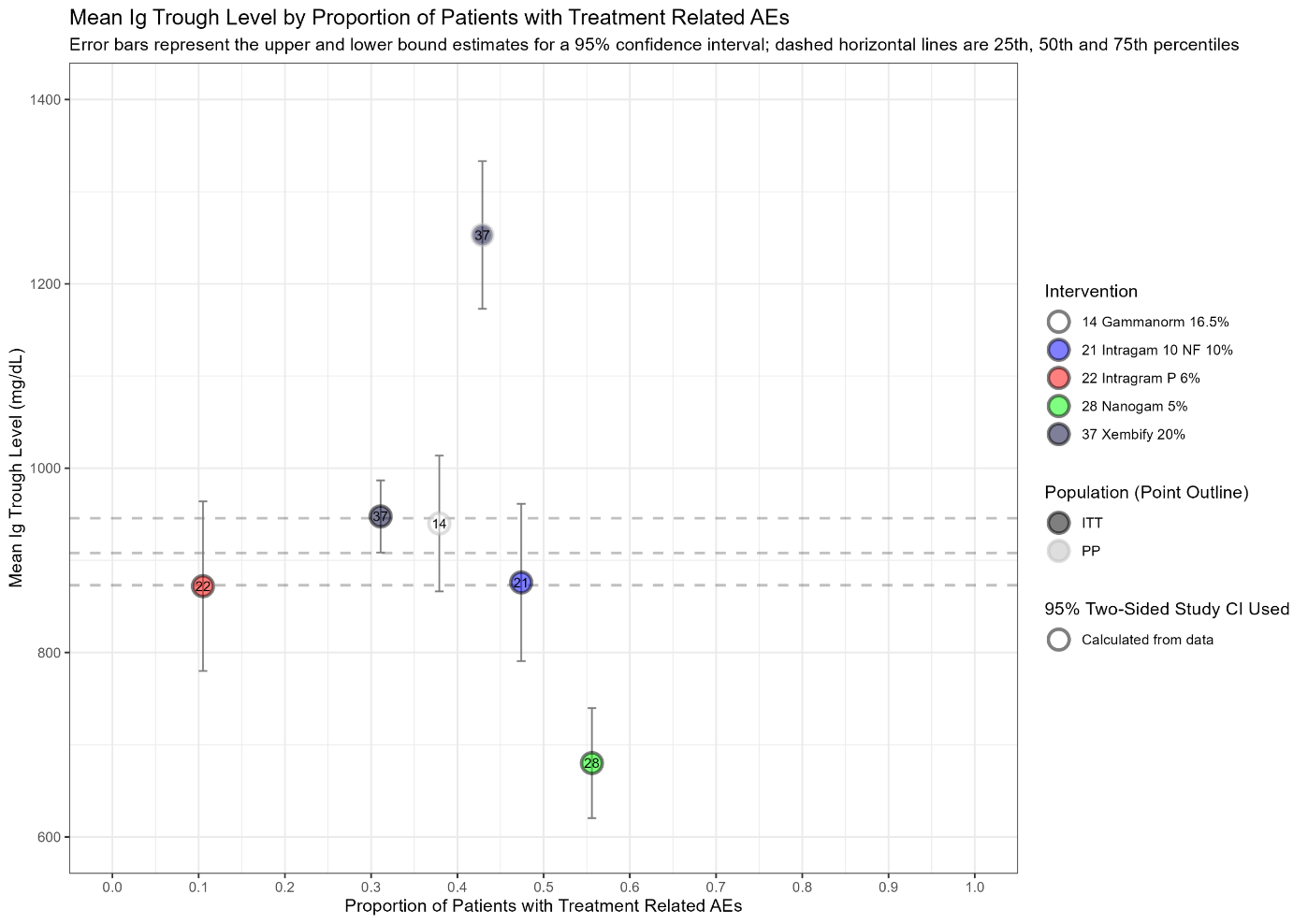


**Quality of life outcomes**

Health related quality-of-life (HRQoL) measures contained within outcomes reports of IgRT were limited and included a range of scales and applications across the individual studies. The SF-36 general health score was the most commonly reported HRQoL but there was not consistency in timepoints of assessment across individual studies thus not allowing for a broader visualization of experience.

Studies of change in SF-36 general health score were also not broadly available but were reported for Vivaglobin 16% SCIG. Three studies were available (with 6 to 12-month follow-up) ^34, 47, 61^. 2 studies reported a statistically significant improvement from baseline to follow-up ^47, 61^ and the remaining study did not test the change from baseline statistically ^34^. These were intended to assess an impact of a transition from intravenous to subcutaneous treatment and were not intended to differentiate any HRQoL impact of a particular preparation over another administered by a given route.

## References

1. Orange JS, Belohradsky BH, Berger M, Borte M, Hagan J, Jolles S*, et al.* Evaluation of correlation between dose and clinical outcomes in subcutaneous immunoglobulin replacement therapy. Clin Exp Immunol. 2012;169(2):172-81.

2. Lee JL, Mohamed Shah N, Makmor-Bakry M, Islahudin FH, Alias H, Noh LM, Mohd Saffian S. A systematic review and meta-regression analysis on the impact of increasing IgG trough level on infection rates in primary immunodeficiency patients on intravenous IgG therapy. J Clin Immunol. 2020;40(5):682-98.

3. Shrestha P, Karmacharya P, Wang Z, Donato A, Joshi AY. Impact of IVIG vs. SCIG on IgG trough level and infection incidence in primary immunodeficiency diseases: a systematic review and meta-analysis of clinical studies. World Allergy Organ J. 2019;12(10):100068.

4. Orange JS, Grossman WJ, Navickis RJ, Wilkes MM. Impact of trough IgG on pneumonia incidence in primary immunodeficiency: a meta-analysis of clinical studies. Clin Immunol. 2010;137(1):21-30.

5. Clarivate. EndNote 20. Clarivate; 2021. Available from: http://endnote.com/.

6. Anderson JT, Bonagura VR, Cowan J, Hsu C, Mustafa SS, Patel NC*, et al.* Safety and tolerability of subcutaneous IgPro20 at high infusion parameters in patients with primary immunodeficiency: findings from the pump-assisted administration cohorts of the HILO study. J Clin Immunol. 2021;41(2):458-69.

7. Bienvenu B, Cozon G, Mataix Y, Lachaud D, Alix A, Hoarau C*, et al.* Rapid push vs pump-infused subcutaneous immunoglobulin treatment: a randomized crossover study of quality of life in primary immunodeficiency patients. J Clin Immunol. 2018;38(4):503-12.

8. Chapel HM, Spickett GP, Ericson D, Engl W, Eibl MM, Bjorkander J. The comparison of the efficacy and safety of intravenous versus subcutaneous immunoglobulin replacement therapy. J Clin Immunol. 2000;20(2):94-100.

9. Desai SH, Chouksey A, Poll J, Berger M. A pilot study of equal doses of 10% IGIV given intravenously or subcutaneously. J Allergy Clin Immunol. 2009;124(4):854-6.

10. Matamoros N, De Gracia J, Hernandez F, Pons J, Alvarez A, Jimenez V. A prospective controlled crossover trial of a new presentation (10% vs. 5%) of a heat-treated intravenous immunoglobulin. Int Immunopharmacol. 2005;5(3):619-26.

11. Roifman CM, Schroeder H, Berger M, Sorensen R, Ballow M, Buckley RH*, et al.* Comparison of the efficacy of IGIV-C, 10% (caprylate/chromatography) and IGIV-SD, 10% as replacement therapy in primary immune deficiency. A randomized double-blind trial. Int Immunopharmacol. 2003;3(9):1325-33.

12. Warnatz K, Jolles S, Agostini C, Vianello F, Borte M, Bethune C*, et al.* Subcutaneous Gammanorm R by pump or rapid push infusion: Impact of the device on quality of life in adult patients with primary immunodeficiencies. Clin Immunol. 2022;236:108938.

13. Wasserman RL, Melamed IR, Stein MR, Jolles S, Norton M, Moy JN, Group GMXS. Evaluation of the safety, tolerability, and pharmacokinetics of Gammaplex R 10% versus Gammaplex R 5% in subjects with primary immunodeficiency. J Clin Immunol. 2017;37(3):301-10.

14. Wolf HH, Davies SV, Borte M, Caulier MT, Williams PE, Bernuth HV*, et al.* Efficacy, tolerability, safety and pharmacokinetics of a nanofiltered intravenous immunoglobulin: studies in patients with immune thrombocytopenic purpura and primary immunodeficiencies. Vox Sang. 2003;84(1):45-53.

15. Bjorkander J, Nikoskelainen J, Leibl H, Lanbeck P, Wallvik J, Lumio JT*, et al.* Prospective open-label study of pharmacokinetics, efficacy and safety of a new 10% liquid intravenous immunoglobulin in patients with hypo- or agammaglobulinemia. Vox Sang. 2006;90(4):286-93.

16. Bleasel K, Heddle R, Hissaria P, Stirling R, Stone C, Maher D. Pharmacokinetics and safety of Intragam 10 NF, the next generation 10% liquid intravenous immunoglobulin, in patients with primary antibody deficiencies. Intern Med J. 2012;42(3):252-9.

17. Borte M, Krivan G, Derfalvi B, Marodi L, Harrer T, Jolles S*, et al.* Efficacy, safety, tolerability and pharmacokinetics of a novel human immune globulin subcutaneous, 20%: a Phase 2/3 study in Europe in patients with primary immunodeficiencies. Clin Exp Immunol. 2017;187(1):146-59.

18. Heimall J, Chen J, Church JA, Griffin R, Melamed I, Kleiner GI. Pharmacokinetics, safety, and tolerability of subcutaneous immune globulin injection (human), 10 % caprylate/chromatography purified (GAMUNEX R-C) in pediatric patients with primary immunodeficiency disease. J Clin Immunol. 2016;36(6):600-9.

19. Kallenberg CGM. A 10% ready-to-use intravenous human immunoglobulin offers potential economic advantages over a lyophilized product in the treatment of primary immunodeficiency. Clin Exp Immunol. 2007;150(3):437-41.

20. Niebur HB, Duff CM, Shear GF, Nguyen D, Alberdi TK, Dorsey MJ, Sleasman JW. Efficacy and tolerability of 16% subcutaneous immunoglobulin compared with 20% subcutaneous immunoglobulin in primary antibody deficiency. Clin Exp Immunol. 2015;181(3):441-50.

21. Sleasman JW, Lumry WR, Hussain I, Wedner HJ, Harris JB, Courtney KL*, et al.* Immune globulin subcutaneous, human - klhw 20% for primary humoral immunodeficiency: an open-label, Phase III study. Immunotherapy. 2019;11(16):1371-86.

22. Suez D, Stein M, Gupta S, Hussain I, Melamed I, Paris K*, et al.* Efficacy, safety, and pharmacokinetics of a novel human immune globulin subcutaneous, 20 % in patients with primary immunodeficiency diseases in North America. J Clin Immunol. 2016;36(7):700-12.

23. Viallard JF, Brion JP, Malphettes M, Durieu I, Gardembas M, Schleinitz N*, et al.* A multicentre, prospective, non-randomized, sequential, open-label trial to demonstrate the bioequivalence between intravenous immunoglobulin new generation (IGNG) and standard IV immunoglobulin (IVIG) in adult patients with primary immunodeficiency (PID). Rev Med Interne. 2017;38(9):578-84.

24. Wasserman RL, Melamed I, Stein MR, Gupta S, Puck J, Engl W*, et al.* Recombinant human hyaluronidase-facilitated subcutaneous infusion of human immunoglobulins for primary immunodeficiency. J Allergy Clin Immunol. 2012;130(4):951-7.e11.

25. Wasserman RL, Irani AM, Tracy J, Tsoukas C, Stark D, Levy R*, et al.* Pharmacokinetics and safety of subcutaneous immune globulin (human), 10% caprylate/chromatography purified in patients with primary immunodeficiency disease. Clin Exp Immunol. 2010;161(3):518-26.

26. Wasserman RL, Melamed I, Kobrynski L, Strausbaugh SD, Stein MR, Sharkhawy M*, et al.* Efficacy, safety, and pharmacokinetics of a 10% liquid immune globulin preparation (GAMMAGARD LIQUID, 10%) administered subcutaneously in subjects with primary immunodeficiency disease. J Clin Immunol. 2011;31(3):323-31.

27. Ballow M. Clinical experience with Flebogamma 5% DIF: a new generation of intravenous immunoglobulins in patients with primary immunodeficiency disease. Clin Exp Immunol. 2009;157(Suppl 1):22-5.

28. Ballow M, Pinciaro PJ, Craig T, Kleiner G, Moy J, Ochs HD*, et al.* Flebogamma( R) 5 % DIF intravenous immunoglobulin for replacement therapy in children with primary immunodeficiency diseases. J Clin Immunol. 2016;36(6):583-9.

29. Berger M, Pinciaro PJ, Flebogamma I. Safety, efficacy, and pharmacokinetics of Flebogamma 5% [immune globulin intravenous (human)] for replacement therapy in primary immunodeficiency diseases. J Clin Immunol. 2004;24(4):389-96.

30. Berger M, Pinciaro PJ, Althaus A, Ballow M, Chouksey A, Moy J*, et al.* Efficacy, pharmacokinetics, safety, and tolerability of Flebogamma 10% DIF, a high-purity human intravenous immunoglobulin, in primary immunodeficiency. J Clin Immunol. 2010;30(2):321-9.

31. Berger M, Murphy E, Riley P, Bergman GE, Investigators VT. Improved quality of life, immunoglobulin G levels, and infection rates in patients with primary immunodeficiency diseases during self-treatment with subcutaneous immunoglobulin G. South Med J. 2010;103(9):856-63.

32. Bezrodnik L, Gomez Raccio A, Belardinelli G, Regairaz L, Diaz Ballve D, Seminario G*, et al.* Comparative study of subcutaneous versus intravenous IgG replacement therapy in pediatric patients with primary immunodeficiency diseases: a multicenter study in Argentina. J Clin Immunol. 2013;33(7):1216-22.

33. Blazek B, Misbah SA, Soler-Palacin P, McCoy B, Leibl H, Engl W*, et al.* Human immunoglobulin (KIOVIG R/GAMMAGARD LIQUID R) for immunodeficiency and autoimmune diseases: an observational cohort study. Immunotherapy. 2015;7(7):753-63.

34. Borte M, Quinti I, Soresina A, Fernandez-Cruz E, Ritchie B, Schmidt DS, McCusker C. Efficacy and safety of subcutaneous vivaglobin R replacement therapy in previously untreated patients with primary immunodeficiency: a prospective, multicenter study. J Clin Immunol. 2011;31(6):952-61.

35. Borte M, Melamed IR, Pulka G, Pyringer B, Knutsen AP, Ochs HD*, et al.* Efficacy and safety of human intravenous immunoglobulin 10% (Panzyga R) in patients with primary immunodeficiency diseases: a two-stage, multicenter, prospective, open-label study. J Clin Immunol. 2017;37(6):603-12.

36. Canessa C, Iacopelli J, Pecoraro A, Spadaro G, Matucci A, Milito C*, et al.* Shift from intravenous or 16% subcutaneous replacement therapy to 20% subcutaneous immunoglobulin in patients with primary antibody deficiencies. Int J Immunopathol Pharmacol. 2017;30(1):73-82.

37. Church JA, Leibl H, Stein MR, Melamed IR, Rubinstein A, Schneider LC*, et al.* Efficacy, safety and tolerability of a new 10% liquid intravenous immune globulin [IGIV 10%] in patients with primary immunodeficiency. J Clin Immunol. 2006;26(4):388-95.

38. Dash C, Gascoigne E, Gillanders K, Gooi H. Experience with subgam, a subcutaneously administered human normal immunoglobulin (ClinicalTrials.gov--NCT02247141). PLoS ONE. 2015;10(7):e0131565.

39. Debes A, Bauer M, Kremer S. Tolerability and safety of the intravenous immunoglobulin Octagam: a 10-year prospective observational study. Pharmacoepidemiol Drug Saf. 2007;16(9):1038-47.

40. Empson MB, Tang MLK, Pearce LKC, Rozen L, Gold MS, Katelaris CH*, et al.* Efficacy, safety and pharmacokinetics of a novel subcutaneous immunoglobulin, Evogam R, in primary immunodeficiency. J Clin Immunol. 2012;32(5):897-906.

41. LFB SA. Long-term safety and efficacy study of igng, a new liquid preparation of human normal immunoglobulin for intravenous use, administered in current practice to primary immunodeficient patients. Identifier: EUCTR2007-001410-17-FR. In: EU Clinical Trials Register [internet]. Amsterdam: European Medicines Agency: 2007. Available from https://trialsearch.who.int/Trial2.aspx?TrialID=EUCTR2007-001410-17-FR.

42. Fasth A, Nystrom J. Quality of life and health-care resource utilization among children with primary immunodeficiency receiving home treatment with subcutaneous human immunoglobulin. J Clin Immunol. 2008;28(4):370-8.

43. Gardulf A, Nicolay U, Asensio O, Bernatowska E, Bock A, Carvalho BC*, et al.* Rapid subcutaneous IgG replacement therapy is effective and safe in children and adults with primary immunodeficiencies--a prospective, multi-national study. J Clin Immunol. 2006;26(2):177-85.

44. Gupta S, DeAngelo J, Melamed I, Walter JE, Kobayashi AL, Bridges T*, et al.* Subcutaneous immunoglobulin 16.5% (Cutaquig®) in primary immunodeficiency disease: Safety, tolerability, efficacy, and patient experience with enhanced infusion regimens. J Clin Immunol. 2023;43(6):1414-25.

45. Gustafson R, Gardulf A, Hansen S, Leibl H, Engl W, Linden M*, et al.* Rapid subcutaneous immunoglobulin administration every second week results in high and stable serum immunoglobulin G levels in patients with primary antibody deficiencies. Clin Exp Immunol. 2008;152(2):274-9.

46. Hagan JB, Fasano MB, Spector S, Wasserman RL, Melamed I, Rojavin MA*, et al.* Efficacy and safety of a new 20% immunoglobulin preparation for subcutaneous administration, IgPro20, in patients with primary immunodeficiency. J Clin Immunol. 2010;30(5):734-45.

47. Hoffmann F, Grimbacher B, Thiel J, Peter HH, Belohradsky BH, Vivaglobin Study G. Home-based subcutaneous immunoglobulin G replacement therapy under real-life conditions in children and adults with antibody deficiency. Eur J Med Res. 2010;15(6):238-45.

48. Jolles S, Bernatowska E, de Gracia J, Borte M, Cristea V, Peter HH*, et al.* Efficacy and safety of Hizentra(R) in patients with primary immunodeficiency after a dose-equivalent switch from intravenous or subcutaneous replacement therapy. Clin Immunol. 2011;141(1):90-102.

49. Kanegane H, Imai K, Yamada M, Takada H, Ariga T, Bexon M*, et al.* Efficacy and safety of IgPro20, a subcutaneous immunoglobulin, in Japanese patients with primary immunodeficiency diseases. J Clin Immunol. 2014;34(2):204-11.

50. Keith PK, Cowan J, Kanani A, Kim H, Lacuesta G, Lee JK*, et al.* Transitioning subcutaneous immunoglobulin 20% therapies in patients with primary and secondary immunodeficiencies: Canadian real-world study. Allergy Asthma Clin Immunol. 2022;18(1):70.

51. Knutsen AP, Leiva LE, Caruthers C, Rodrigues J, Sorensen RU. Streptococcus pneumoniae antibody titres in patients with primary antibody deficiency receiving intravenous immunoglobulin (IVIG) compared to subcutaneous immunoglobulin (SCIG). Clin Exp Immunol. 2015;182(1):51-6.

52. Kobayashi RH, Gupta S, Melamed I, Mandujano JF, Kobayashi AL, Ritchie B*, et al.* Clinical efficacy, safety and tolerability of a new subcutaneous immunoglobulin 16.5% (Octanorm [Cutaquig(R)]) in the treatment of patients with primary immunodeficiencies. Front Immunol. 2019;10:40.

53. Krasovec S, Ornani A, Oleastro M, Rosenzweig S, Roy A, Perez L*, et al.* Efficacy and tolerability of an argentine intravenous immunoglobulin in pediatric patients with primary immunodeficiency diseases. J Clin Immunol. 2007;27(2):227-32.

54. Kreuz W, Erdos M, Rossi P, Bernatowska E, Espanol T, Marodi L. A multi-centre study of efficacy and safety of Intratect R, a novel intravenous immunoglobulin preparation. Clin Exp Immunol. 2010;161(3):512-7.

55. Krivan G, Chernyshova L, Kostyuchenko L, Lange A, Nyul Z, Derfalvi B*, et al.* A multicentre study on the efficacy, safety and pharmacokinetics of IqYmune R, a highly purified 10% liquid intravenous immunoglobulin, in patients with primary immune deficiency. J Clin Immunol. 2017;37(6):539-47.

56. Krivan G, Borte M, Harris JB, Lumry WR, Aigner S, Lentze S, Staiger C. Efficacy, safety and pharmacokinetics of a new 10% normal human immunoglobulin for intravenous infusion, BT595, in children and adults with primary immunodeficiency disease. Vox Sang. 2022;117(10):1153-62.

57. Latysheva E, Rodina Y, Sizyakina L, Totolian A, Tuzankina I. Efficacy and safety of octanorm (cutaquig R) in adults with primary immunodeficiencies with predominant antibody deficiency: a prospective, open-label study. Immunotherapy. 2020;12(5):299-309.

58. Melamed IR, Gupta S, Stratford Bobbitt M, Hyland N, Moy JN. Efficacy and safety of Gammaplex( R) 5% in children and adolescents with primary immunodeficiency diseases. Clin Exp Immunol. 2016;184(2):228-36.

59. Moy JN, Scharenberg AM, Stein MR, Suez D, Roberts RL, Levy RJ*, et al.* Efficacy and safety of a new immunoglobulin G product, Gammaplex (R), in primary immunodeficiency diseases. Clin Exp Immunol. 2010;162(3):510-5.

60. Nicolay U, Haag S, Eichmann F, Herget S, Spruck D, Gardulf A. Measuring treatment satisfaction in patients with primary immunodeficiency diseases receiving lifelong immunoglobulin replacement therapy. Qual Life Res. 2005;14(7):1683-91.

61. Nicolay U, Kiessling P, Berger M, Gupta S, Yel L, Roifman CM*, et al.* Health-related quality of life and treatment satisfaction in North American patients with primary immunedeficiency diseases receiving subcutaneous IgG self-infusions at home. J Clin Immunol. 2006;26(1):65-72.

62. Ochs HD, Pinciaro PJ. Octagam 5%, an intravenous IgG product, is efficacious and well tolerated in subjects with primary immunodeficiency diseases. J Clin Immunol. 2004;24(3):309-14.

63. Ochs HD, Gupta S, Kiessling P, Nicolay U, Berger M, Subcutaneous Ig GSG. Safety and efficacy of self-administered subcutaneous immunoglobulin in patients with primary immunodeficiency diseases. J Clin Immunol. 2006;26(3):265-73.

64. Perez EE, Hebert J, Ellis AK, Alpan O, Lumry WR, Shapiro R*, et al.* Efficacy, safety and tolerability of a new 10% intravenous immunoglobulin for the treatment of primary immunodeficiencies. Front Immunol. 2021;12:707463.

65. Santaella ML, Font I, Disdier O. Common variable immunodeficiency: experience in Puerto Rico. P R Health Sci J. 2005;24(1):7-10.

66. Santamaria M, Neth O, Douglass JA, Krivan G, Kobbe R, Bernatowska E*, et al.* A multi-center, open-label, single-arm trial to evaluate the efficacy, pharmacokinetics, and safety and tolerability of IGSC 20% in subjects with primary immunodeficiency. J Clin Immunol. 2022;42(3):500-11.

67. Stein MR, Nelson RP, Church JA, Wasserman RL, Borte M, Vermylen C*, et al.* Safety and efficacy of Privigen, a novel 10% liquid immunoglobulin preparation for intravenous use, in patients with primary immunodeficiencies. J Clin Immunol. 2009;29(1):137-44.

68. Stein M, Nemet A, Kumar S, et al. Efficacy, safety, and tolerability of Kedrion 10% IVIG in primary immunodeficiency. LymphoSign Journal. 2016;3(3):99-109.

69. Tcheurekdjian H, Martin J, Kobayashi R, Wasserman R, Hostoffer R. Intrainfusion and postinfusion adverse events related to intravenous immunoglobulin therapy in immunodeficiency states. Allergy Asthma Proc. 2006;27(6):532-6.

70. Tuerlinckx D, Florkin B, Ferster A, De Schutter I, Chantrain C, Haerynck F*, et al.* Pneumococcal antibody levels in children with PID receiving immunoglobulin. Pediatrics. 2014;133(1):e154-62.

71. van der Meer JWM, van Beem RT, Robak T, Deptala A, Strengers PFW. Efficacy and safety of a nanofiltered liquid intravenous immunoglobulin product in patients with primary immunodeficiency and idiopathic thrombocytopenic purpura. Vox Sang. 2011;101(2):138-46.

72. Vultaggio A, Azzari C, Milito C, Finocchi A, Toppino C, Spadaro G*, et al.* Subcutaneous immunoglobulin replacement therapy in patients with primary immunodeficiency in routine clinical practice: the VISPO prospective multicenter study. Clin Drug Invest. 2015;35(3):179-85.

73. Vultaggio A, Azzari C, Ricci S, Martire B, Palladino V, Gallo V*, et al.* Biweekly Hizentra R in primary immunodeficiency: a multicenter, observational cohort study (IBIS). J Clin Immunol. 2018;38(5):602-09.

74. Wasserman RL, Church JA, Stein M, Moy J, White M, Strausbaugh S*, et al.* Safety, efficacy and pharmacokinetics of a new 10% liquid intravenous immunoglobulin (IVIG) in patients with primary immunodeficiency. J Clin Immunol. 2012;32(4):663-9.

75. Wasserman RL, Lumry W, Harris J, 3rd, Levy R, Stein M, Forbes L*, et al.* Efficacy, safety, and pharmacokinetics of a new 10 % liquid intravenous immunoglobulin containing high titer neutralizing antibody to RSV and other respiratory viruses in subjects with primary immunodeficiency disease. J Clin Immunol. 2016;36(6):590-9.

76. Dinnes J CC, Huang S, Major K, Milne R. The effectiveness and costeffectiveness of temozolomide for the treatment of recurrent malignant glioma: a rapid and systematic review. Health Technology Assessment. 2001;5(13):1-73.
